# Disruptive effects of phthalates and their substitutes on adrenal steroidogenesis

**DOI:** 10.3389/fendo.2025.1734184

**Published:** 2026-01-14

**Authors:** Benedikt Pötzl, Max Kurlbaum, Sabine Kendl, Lydia Kürzinger, Sabine Herterich, Simon Kloock, Martin Fassnacht, Ulrich Dischinger

**Affiliations:** 1 Division of Endocrinology and Diabetology, Department of Internal Medicine I, University Hospital of Würzburg, Würzburg, Germany; 2 Core Unit Clinical Mass Spectrometry, University Hospital, University of Würzburg, Würzburg, Germany; 3 Central Laboratory, University Hospital of Würzburg, Würzburg, Germany

**Keywords:** adrenal, DEHP, DINCH, endocrine disruptor, mineralocorticoid, phthalate, plasticizer, steroidogenesis

## Abstract

**Introduction:**

Phthalates are ubiquitous plasticizers known for their endocrine-disrupting properties, notably affecting reproductive and cardiovascular health. Emerging substitutes such as DEHT and DINCH are increasingly use, but may turn out to be “regrettable substitutes” with similar toxicological concerns. Though the effects of phthalates and substitutes on adrenal steroidogenesis and related endocrine systems (e.g., renin-angiotensin-aldosterone system, hypothalamic-pituitary axis) remain poorly understood.

**Methods:**

In this study, steroidogenic NCI-H295R adrenocortical cells were exposed for 72 hours to phthalates (DEHP, DiBP, DiNP), substitutes (DEHA, DEHT, DINCH), and a cumulative mixture at concentrations ranging from 1 nM to 1 mM. DMSO vehicle controls were included in all experiments. Cytotoxicity was assessed using standard cell viability assays, while steroid secretion was quantified by LC–MS/MS, covering 15 adrenal steroids. Relative enzymatic activities were estimated from steroid ratios. mRNA expression of key molecules involved in adrenocortical steroidogenesis was analyzed by RT-qPCR.

**Results:**

Cortisol, 21-deoxycortisol, corticosterone, and aldosterone were significantly increased after treatment with DEHP, DiNP, DEHT, DINCH, and their combinatory mixture at non-cytotoxic doses (e.g., corticosterone 6.51-fold increase at 5 µM DEHP). Phthalates and substitutes dysregulated steroidogenic enzyme activity, notably inhibiting HSD11B2’s conversion of cortisol to cortisone below 25% in relation to controls. Combinatory exposure led to an increased mRNA expression of CYP11B1 (11.8-fold at 10 µM) and CYP11B2 (44.1-fold at 10 µM) as well as other steroidogenic enzymes (e.g., CYP21A2, HSD3B2) and key adrenocortical receptors (e.g., MC2R, AGTR1) when compared to untreated controls.

**Discussion:**

This in vitro study provides novel evidence on phthalate- and substitute-induced endocrine disruption of adrenal steroidogenesis, favouring mineralo- and glucocorticoid secretion, potentially linking these substances to secondary hypertension. Notably, emerging substitute substances (e.g., DEHT, DINCH) showed similar effects of adrenal disruption, compared to classical phthalates.

## Graphical Abstract

**Figure:**
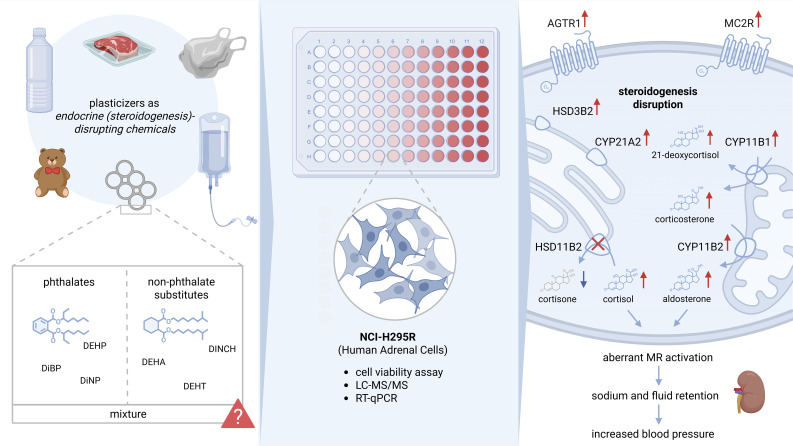
Integrated *in vitro* workflow to evaluate plasticizer–induced alterations in adrenal steroidogenesis. This figure was created using Biorender (Toronto, Canada).

## Introduction

Plastic pollution is a critical environmental concern, exceeding planetary boundary levels and threatening stable and healthy living conditions for both humans and wildlife (1). Plastics are synthetic polymers stabilized with various additives to confer properties like flexibility, durability, and resistance to heat and radiation. Among these, plasticizers account for 10–70% of the material by weight. Phthalate diesters, including di-(2-ethylhexyl)phthalate (*DEHP*), or di-isobutylphthalate (*DiBP*), represent up to 85% of global plasticizer production (2). Over 90% of plasticizers are used to produce polyvinyl chloride (*PVC*), found in food packaging, building materials, cosmetics, or toys (3), and medical equipment, like infusion bags (4), drug formulations (5), or face masks (6). As phthalates are not covalently bound in these materials, they leach via volatilization, migration, or abrasion during production, use, and disposal (3).

Humans are mainly exposed to phthalates via ingestion, especially through ultra-processed food and its packaging (7, 8). But also inhalation of indoor dust (9), dermal incorporation from cosmetic and personal care products (10), and medical equipment are proven ways of exposure. Consequently, phthalates and their respective monoester metabolites have been detected in blood, urine, breast milk, saliva, and amniotic fluid, and were ubiquitously found in air, water, or sediment (11).

Systematic research in the last decades revealed the endocrine-disrupting properties of phthalates in multiple association studies (12, 13). In addition to metabolic disorders and endocrine-related cancer entities, phthalates were associated with the impairment of the reproductive system. Increased rates of cryptorchidism, hypospadias, testicular cancer, and reduced sperm quality have led to the concept of testicular dysgenesis syndrome, linking phthalates with steroid disruption (14). Moreover, phthalates might constitute a major global cardiovascular health hazard, as an association between DEHP exposure and 13.5% of all cardiovascular deaths in 55 to 64-year-old patients was reported (15).

The adrenal cortex, as a source of different steroids, plays an essential role in stress response, fluid balance, and blood pressure regulation, making it a critical, clinically relevant, and yet understudied target for endocrine disruption (16). Studies show inconsistent results regarding the effects of phthalates on adrenal function, with reports of both increased (17–20) and decreased (19, 21) levels of cortisol and cortisol-cortisone ratios. In addition, prenatal DEHP exposure has been linked to disrupted androgen (e.g., DHEA, androstenedione) (21) and gestagen secretion (22). Supporting these observations, *in vitro* adrenal cell culture experiments demonstrated phthalate-induced changes in the expression and activity of steroidogenic enzymes (23–25). Particularly, aldosterone and cortisol synthesis - as well as the corresponding catalyzing enzymes CYP11B1 and CYP11B2 - have emerged as prominent targets of phthalate action (26, 27). Beyond direct adrenal effects, animal studies suggest that phthalates disrupt the hypothalamic–pituitary–adrenal (*HPA*) axis, increase adrenocorticotropic hormone (*ACTH*) levels, modulate glucocorticoid-receptor expression, and disturb hypothalamic corticotropin-releasing hormone (*CRH*) homeostasis (28–30). Moreover, phthalates may interfere with the renin–angiotensin–aldosterone system (*RAAS*), by enhancing mineralocorticoid receptor (*MR*) activation or altering downstream signaling pathways (26, 31, 32).

In response to substantial health concerns, most high-income countries have introduced regulations based on probable intake levels for phthalates. The European Chemicals Agency (*ECHA*) lists five phthalates (DiBP, DBP, BBP, DEHP, DCHP) as substances of very high concern (*SVHC*) for their “endocrine disrupting properties”, requiring industry notification before use (33). In addition, several phthalates are included in the REACH (*Registration, Evaluation, Authorization and Restriction of Chemicals)* authorization list, restricting their use in specific applications (34). Consecutively, these tighter regulations have driven the development of alternatives to hazardous phthalates, including non-phthalate plasticizers such as adipates, benzoates, terephthalates, citrates, sebacates, cyclohexane dicarboxylic acids, and bio-based compounds (35). Among these, diisononyl cyclohexane-1,2-dicarboxylate (*DINCH*), di(2-ethylhexyl-) adipate (*DEHA*), or di(2-ethylhexyl) terephthalate (*DEHT*) have emerged as leading substitutes (**Table 1**).

**Table 1:** Overview of selected relevant phthalates and phthalate alternatives included in this study.

**Table 1**	Chemical properties (structure, formula, molecular weight, main monoester metabolites)	Main uses, exposure routes	EU production/import volume (ECHA)	Regulatory status
DEHP; di-(2-ethylhexyl)phthalate	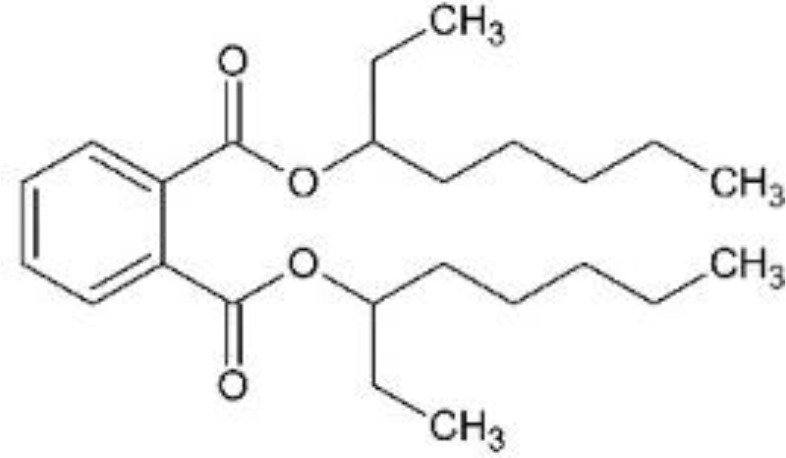 − C_24_H_38_O_4_; 390.56 g/mol − MEHP, MEHHP, MEOHP	− Plasticizer in PVC (medical tubing, flooring, cables, food packaging) − High migration potential from plastics into food, IV fluids, dust − widespread detection in biomonitoring samples globally (37)	− Listed as high-production volume (*HPV*) chemical (65); 10,000 to 100,000 tons per year in EU (66) − Declining market trends (in EU)	− Tolerable daily intake (*TDI*) (EFSA): 0.05 mg/kg bodyweight/day (39) − Substance of very high concern (*SVHC*) in the European Union (33); under risk evaluation in the Toxic Substances Control Act (*TSCA*) in the US (46) − REACH Restriction (Annex XVII) and Authorization (Annex XIV) (36); required authorization before use
DiBP; di-isobutylphthalate	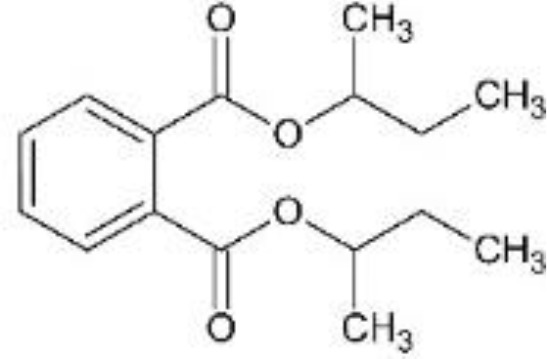 − C_16_H_22_O_4_; 278.35 g/mol − MiBP, OH-MiBP	− Plasticizer in plastics, adhesives, coatings − widespread detection in biomonitoring samples globally (67)	− Listed as HPV chemical (65); >1,000 tons per year in EU (66) − Declining market trends	− TDI (EFSA): 0.01 mg/kg bodyweight/day (39) − SVHC (33); TSCA (68, 68) − REACH Restriction (Annex XVII) and Authorization (Annex XIV) (36)
DiNP; di-isononylphthalate	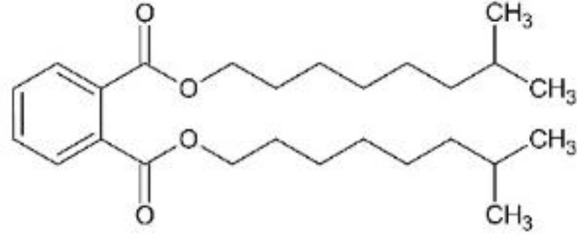 − C_26_H_42_O_4_; 418.62 g/mol − MINP, OH-MINP	− Plasticizer in PVC (medical tubing, flooring, cables, food packaging) − Increasing detection frequency in biomonitoring (36)	− Listed as HPV chemical (65); 100,000 to 1,000,000 tons per year in EU (66) − Increasing market trends, replacing DEHP	− TDI (EFSA): 0.15 mg/kg bodyweight/day (69) − REACH Restriction (Annex XVII); product-related restrictions (e.g., cosmetics) (36)
DEHA; di-(2-ethylhexyl) adipate	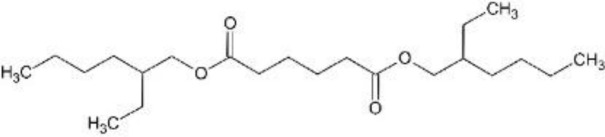 − C_22_H_42_O_4_; 370.57 g/mol − MEHA	− “Phthalate-free” plasticizer (especially food packaging, medical devices) (43) − Limited evidence for endocrine activity	− Listed as HPV chemical (65); 10,000 – 100,000 tons per year in EU (66)	− Permitted with limits (EFSA, FDA) − Included in Community Rolling Action Plan (CoRAP) (36)
DEHT; di-(2-ethylhexyl)terephthalate	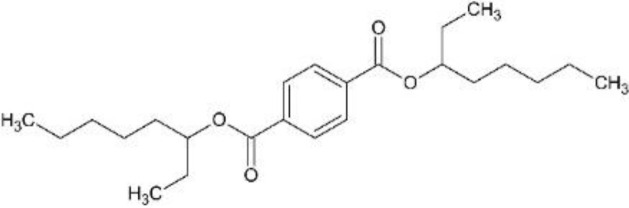 − C_24_H_38_O_4_; 390.56 g/mol − MEHT	− “Phthalate-free” plasticizer alternative (especially children’s products; food packaging, medical devices) (43) − Lower migration rate than DEHP (70) − Increasing detection frequency in biomonitoring (36, 67, 71)	− Listed as HPV chemical (65); 100,000 – 1,000,000 tons per year in EU (66) − Increasing market trends; major phthalate substitute	− TDI (EFSA): 1mg/kg bodyweight/day (72) − No current restriction (EU/US) (36)
DINCH; di-isononyl-1,2-cyclohexanedicarboxylic acid	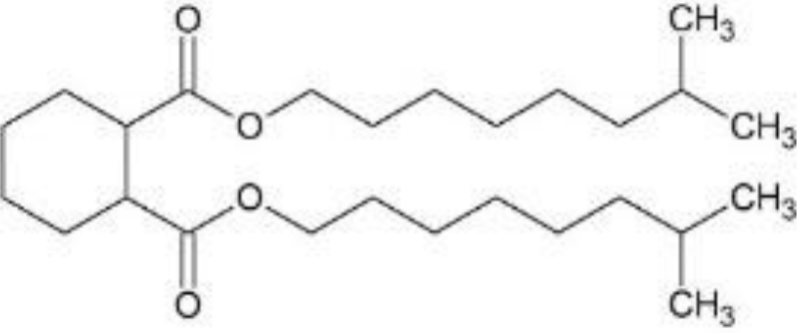 − C_26_H_48_O_4_; 424.67 g/mol − MINCH, OH-MINCH	− “Phthalate-free” plasticizer (especially children’s products; food packaging, medical devices) (73) − Lower migration rate than DEHP (70); − Increasing detection frequency in biomonitoring (36, 67, 71) − Mixed evidence for endocrine disruption (73)	− >10,000 tons/year in EU (66) − Increasing market trends; major phthalate substitute	− No current restriction (EU/US) (36)

Explanation of abbreviations is found in the **Supplementary Table S1**. High-production volume chemicals, following OECD definition, are produced more than 1,000 metric tons per producer/importer per year in at least one member country/region (65). ECHA substance information refers to the production or import of substance volume in the European Economic Area (66).

This trend is already evident in environmental biomonitoring, which shows a shift from restricted, formerly widely used phthalates (e.g., DEHP) to other phthalates (e.g., DiBP, di-isononylphthalate (*DiNP*)) and non-phthalate plasticizers (e.g., DEHT, DINCH). Notably, total detectable plasticizer levels remained stable, while detection rates of high-molecular and non-phthalate plasticizers, such as DEHT and DINCH, increased globally in human urine samples (36). In light of these shifts in use and environmental distribution of substances, there is a concern that non-phthalate plasticizers may represent ‘regrettable substitutes’ with similar patterns of distribution, environmental fate, and potentially harmful toxicological properties (35). While individual exposure strongly depends on sociodemographic factors (e.g., age, sex, geographical location, socioeconomic status) as well as study design (e.g., biological matrix, analytical method), geometric means of urinary metabolite concentrations in European children have been reported at 33.6 µg/L (P95: 127 µg/L) for DEHP, 8.31 µg/L (P95: 43.1 µg/L) for DiNP.

Regarding the adrenal steroidogenesis disruptive effects of these substitutes have been noted *in vitro* recently: DEHA, DINCH, DEHT, and DINP, led to an increase in estradiol secretion (25, 37–39) and a high potential to interact with steroid receptors (e.g., androgen- or estrogen-receptor) (37, 40). Nevertheless, their effect on adrenal mineralo- and glucocorticoids has not been systematically investigated. Moreover, humans are typically exposed to mixtures of plasticizers, which may exert additive or synergistic toxic effects. Consequently, toxicological assessments should shift towards mixture-based approaches that consider additive effects and interactions. This is particularly relevant for regulatory frameworks aiming to protect vulnerable populations, such as children or newborns. Indeed, recent biomonitoring studies indicate that 17% of European children and adolescents exceed the safe cumulative exposure threshold for five commonly used phthalates, even though individual compound levels remain below established safety limits (41).

In light of these aspects, the present study employed the adrenocortical carcinoma cell line NCI-H295R to evaluate the multifaceted effects of prominent phthalates (DEHP, DiBP, DiNP) and some emerging substitute plasticizers (DEHA, DEHT, DINCH), as well as their mixture on adrenal steroidogenesis, with focus on mineralocorticoid and glucocorticoid secretion. This cell line expresses all steroidogenic enzymes and secretes physiologically relevant steroids, making it a suitable screening tool for detecting steroidogenesis disruption (42) and a standard model in protocols for the admission of newly introduced chemicals (43).

## Materials and methods

### Chemicals

Di-(2-ethylhexyl)phthalate (bis(2-ethylhexyl) benzene-1,2-dicarboxylate; DEHP), di-isobutylphthalate (bis(2-methylpropyl) benzene-1,2-dicarboxylate; DiBP), di-isononylphthalate (bis(7-methyloctyl) benzene-1,2-dicarboxylate; DiNP), di-(2-ethylhexyl) adipate (bis(2-ethylhexyl) hexanedioate; DEHA), and di-(2-ethylhexyl)terephthalate (bis(2-ethylhexyl) benzene-1,4-dicarboxylate; DEHT; DEHTP) were obtained from Sigma-Aldrich, St. Louis, Missouri, USA. Di-isononyl-1,2-cyclohexanedicarboxylic acid (bis(7-methyloctyl)-cyclohexane-1,2-dicarboxylate; Hexamoll^®^ DINCH; DINCH) was obtained from BASF, Ludwigshafen am Rhein, Germany. All chemicals were dissolved in dimethyl sulfoxide (*DMSO*) (Sigma-Aldrich, St. Louis, Missouri, USA), and stored at -20°C. For mixture exposures, an equimolar six-component mixture was prepared by combining each compound at identical molar proportions (DEHP: DiBP: DiNP: DEHA: DEHT: DINCH = 1: 1: 1: 1: 1: 1). The final mixture concentration applied to cells reflects the sum of all six components.

### Cell culture

NCI-H295R cells were cultured in Dulbecco’s modified Eagle’s medium/F-12 medium (Gibco, Burlington, Ontario, USA), including 10% fetal bovine serum (Sigma-Aldrich, StLouis, Missouri, USA), and 1% insulin-transferrin-selenium (Sigma-Aldrich, St. Louis, Missouri, USA). Cells were kept at 37°C and 5% CO_2_ in 175 cm^2^ flasks (Greiner bio-one, Frickenhausen, Germany), medium was renewed every second day.

Once a week, given sufficient cell density assessed by microscopic control, cells were harvested using phosphate-buffered saline (Sigma-Aldrich, St. Louis, Missouri, USA), and EDTA-trypsin (Sigma-Aldrich, St. Louis, Missouri, USA). For cell counting the Countess II FL (Life Technologies, Carlsbad, California, USA) was used, and if not needed for cell treatments, remaining cells were split into new cell flasks, sticking to a 1:3 ratio.

### Cell treatment

NCI-H295R cells) were disseminated in 96-well black plates (Corning Incorporated, Corning, New York, USA) with optically clear flat bottoms. A seeding density of 5x10^4^/well was used in a total volume of 100 µL of complete medium. After 24 h of incubation at 37°C and 5% CO_2,_ cells were treated with the vehicle DMSO (1%), DEHP, DiBP, DiNP, DEHA, DEHT, or DINCH, of 0.001, 0.05, 0.1, 0.25, 0.5, 1, 10, 25, 100, 250, and 1000 µM. For the combinatory treatment, the six-component-mixture was used in the same concentrations as in the single-substance treatments. Working solutions were obtained using the DMSO-dissolved stocks and diluted in complete medium, reaching a final DMSO concentration of 1%. In all experiments, concentrations are reported and interpreted on a nominal basis. A total volume of 100 µL was added to each well. Cells were exposed to the treatment for 72 h. After the end of the incubation, 150 µL supernatant of eight identically treated wells was collected, pooled, centrifuged at 800 rpm at 23°C, and stored at -20°C for later steroid quantification. Each treatment was performed in three independent experiments. Concentration selection and overall experimental framework were based on OECD Test Guideline 456 with minor adaptations to fit the specific objectives and constraints of the present study (43).

### Cell viability assay

Remaining cells were analyzed for cell viability after treatment using CellTiter-Glo^®^ Luminescent assay (Promega, Madison, Wisconsin, USA) following the manufacturer’s protocol. Cells were incubated with CellTiter-Glo^®^ reagent for 10 min, and luminescence was determined with 1420 multilabel counter Victor^3^ (Perkin Elmer, Waltham, Massachusetts, USA). Each experiment was performed in three independent runs in 8-plicates.

### Steroid hormone analysis

To analyze the steroid metabolome, liquid chromatography tandem mass spectrometry (*LC-MS/MS*) using a Sciex 6500+ QTRAP (SCIEX, Framingham, USA) MS-system linked with an Agilent 1290 HPLC-system (G4226A, autosampler, infinityBinPump, G1316C column-oven, G1330B thermostat; Santa Clara, USA) was utilized. Using the MassChrom-Steroids in Serum Plasma^®^ IVDR conform kit (Chromsystems^®^, Gräfelfing, Germany) (44) 15 steroids (aldosterone, androstenedione, corticosterone, cortisol, cortisone, dehydroepiandrosterone (*DHEA*), dehydroandrosterone-sulfate (*DHEAS*), 11-deoxycorticosterone, 11-deoxycortisol, 21-deoxycortisol, dihydrotestosterone (*DHT*), estradiol, 17-hydroxyprogesterone (*17-OHP*), progesterone, and testosterone) were quantified. After off-line solid phase extraction of 500 µl medium supernatant, 15 µL were used for analysis. With Analyst Software (1.6.3), concentrations were calculated via six-point calibration and 1/x weighing. Commercial quality controls and periodic participations in ring trails ensured the correctness of the described measurements. Absolute steroid values and limits of quantification per analyte are provided in **Supplementary Tables S6, S7**.

### RNA extraction and cDNA synthesis

NCI-H295R cells were seeded in 12-well plates (Corning Incorporated, Corning, NY, USA) at a density of 1 × 10^6^ cells/well and exposed to 1% DMSO or the combinatory mixture of all six test substances, each applied at concentrations of 0.25, 0.5, 1, 2.5, 5, 10, and 25μµM. After 72μhours of exposure, cells were harvested using PBS and EDTA-trypsin, centrifuged, and washed with PBS. Total RNA was extracted using the Maxwell^®^ RSC simplyRNA Tissue Kit (Promega, Madison, WI, USA) according to the manufacturer’s instructions and stored at −80°C until further use.

A total of 1 µg RNA per sample was reverse transcribed using the QuantiTect^®^ Reverse Transcription kit (Qiagen, Venlo, Netherlands) in a final volume of 40 µL according to manufacturer’s protocol. cDNA was diluted by 1:2 in nuclease-free water and stored at -20°C for further analysis. NRT controls were included to confirm the absence of genomic DNA contamination.

### Duplex quantitative real-time PCR

Duplex real-time PCR was conducted to simultaneously quantify β-actin (*ACTB*) as a housekeeping gene and various genes of interest (*StAR, CYP11B1, CYP11B2, CYP17A1, CYP21A2, HSD3B2, AGTR1, MC2R, SF-1*). Primer/probe interactions (FAM- and CY5-labeled) were evaluated in both single and duplex reactions to confirm specificity and amplification efficiency. Primer sequences and TaqMan Assay IDs are provided in the **Supplementary Tables S2**. Reactions were performed using a C1000 Touch Thermal Cycler and CFX96 Real Time System (Bio-Rad, Hercules, CA, USA) in a final volume of 20 µL, containing 6µL nuclease-free water, 10 µL TaqMan^®^ Gene Expression Master Mix (Thermo Fisher Scientific, Waltham, MA, USA), 1 µL of CY5-labeled *Actb* primer/probe mix, 1 µL of FAM-labeled gene-specific primer/probe mix (Thermo Fisher Scientific) and 2 µL of diluted cDNA per reaction. Amplification was conducted under the following conditions: initial hold at 50°C for 2 minutes, followed by enzyme activation and initial denaturation at 95°C for 10 minutes. Then, 50 amplification cycles, each consisting of denaturation at 95°C for 15 seconds, and annealing/extension at 60°C for 1 minute, during which fluorescence data were collected.

mRNA expression was quantified using the comparative Ct (2^−ΔΔCt^) method. Each condition was analyzed in three independent biological replicates and three technical. Results are provided in **Supplementary Table S5**.

### Statistical analysis

Statistical analysis was performed with GraphPad Prism Software (version 9.01 for Windows, GraphPad Software, Inc.). Data regarding cell viability assays, steroid quantification, calculated substrate-product ratios, and mRNA expression levels in response to chemical treatment were calculated as fold changes compared to vehicle-treated controls. All data are expressed as mean ± standard deviation (SD) unless otherwise indicated. For statistical analysis, one-way ANOVA followed by Dunnett’s *post hoc* test was performed using vehicle-treated controls as reference. A *p*-value <0.05 was considered statistically significant.

To assess potential alterations in enzyme-dependent steroidogenic steps, substrate-to-product ratios were calculated for each conversion and normalized to vehicle controls using the following formula, indicating relative activity:


$$\frac{substrate/product}{substrate\;(1\%DMSO)/product\;(1\%DMSO)}$$

Deviations indicate alterations in steroidogenic enzyme activity. Specifically, values >1 suggest an inhibition of the respective enzymatic activity, while values <1 indicate a potential enhancement.

Chemical structures were designed in ChemSketch (FreeWare) 2024.1.4 (ACD/Labs, Toronto, Canada).

## Results

### Cell viability

**Figure 1:**
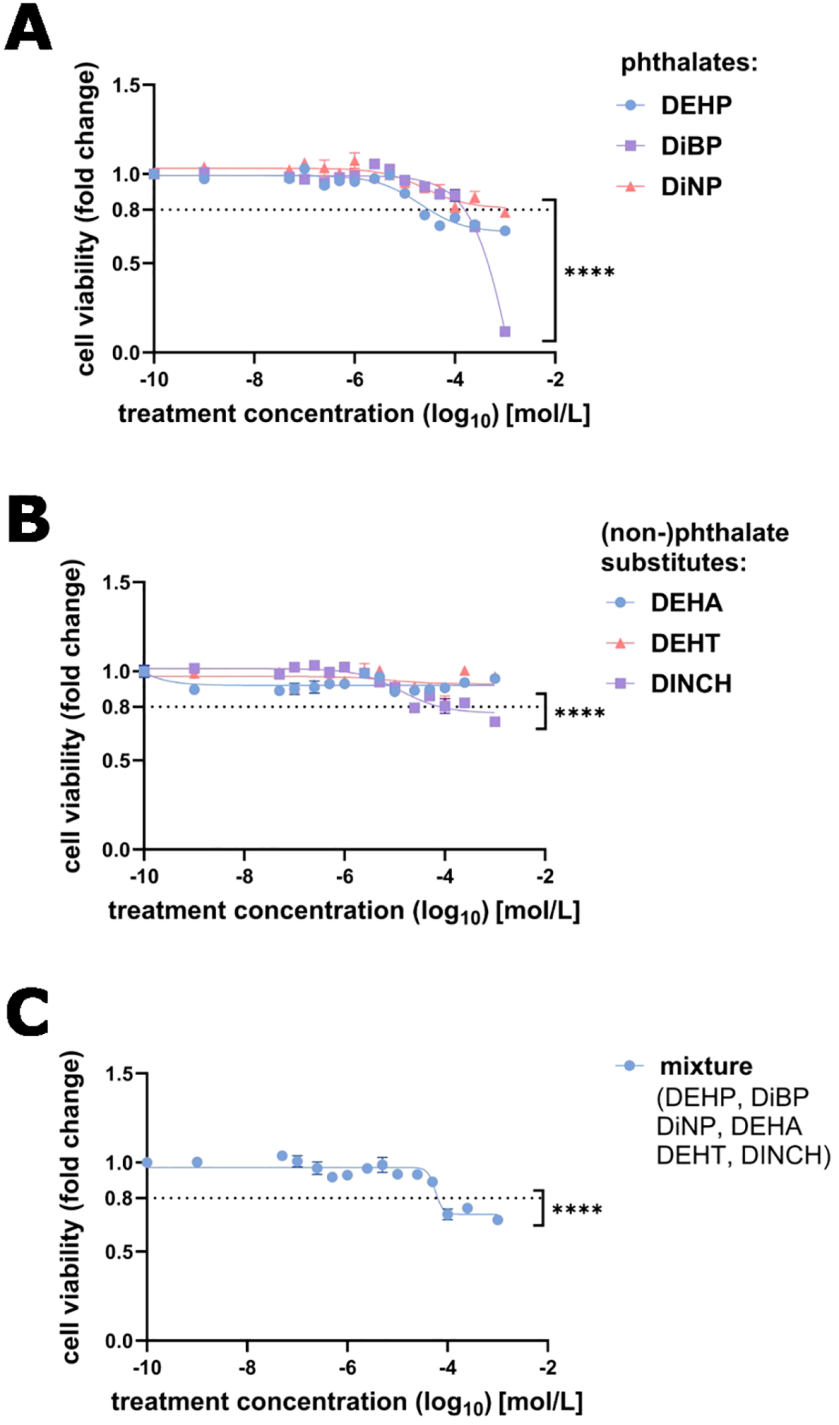
Cell viability of NCI-H295R cells after 72 h exposure with phthalates (DEHP, DiBP, or DiNP) **(A)**, non-phthalate substitutes (DEHA, DEHT, or DINCH) **(B)**, or the combined mixture **(C)**, in relation to vehicle controls. Cell viability (mean ± SEM, n = 3) was expressed as fold change. Values below 80% viability suggested treatment-specific effects. Statistical significance compared to vehicle control: **** p≤ 0.0001.

Cell viability was not affected by vehicle (1% DMSO) and low-dose treatment (<100 µM) of DEHP, DiBP, DiNP, DEHA, DEHT, DINCH, and the combined mixture after 72 hours of treatment (**Figure 1**). However, cell viability decreased significantly below 80% at concentrations greater than 100 µM of DEHP, DiBP, DiNP, and the mixture. The calculated median lethal concentration (*LC_50_*) for DiBP was 1.91 mM. Since none of the other treatments reduced viability below 50%, no representative LC_50_ values were determined for these compounds.

### Steroidomics

#### DEHP

**Figure 2:**
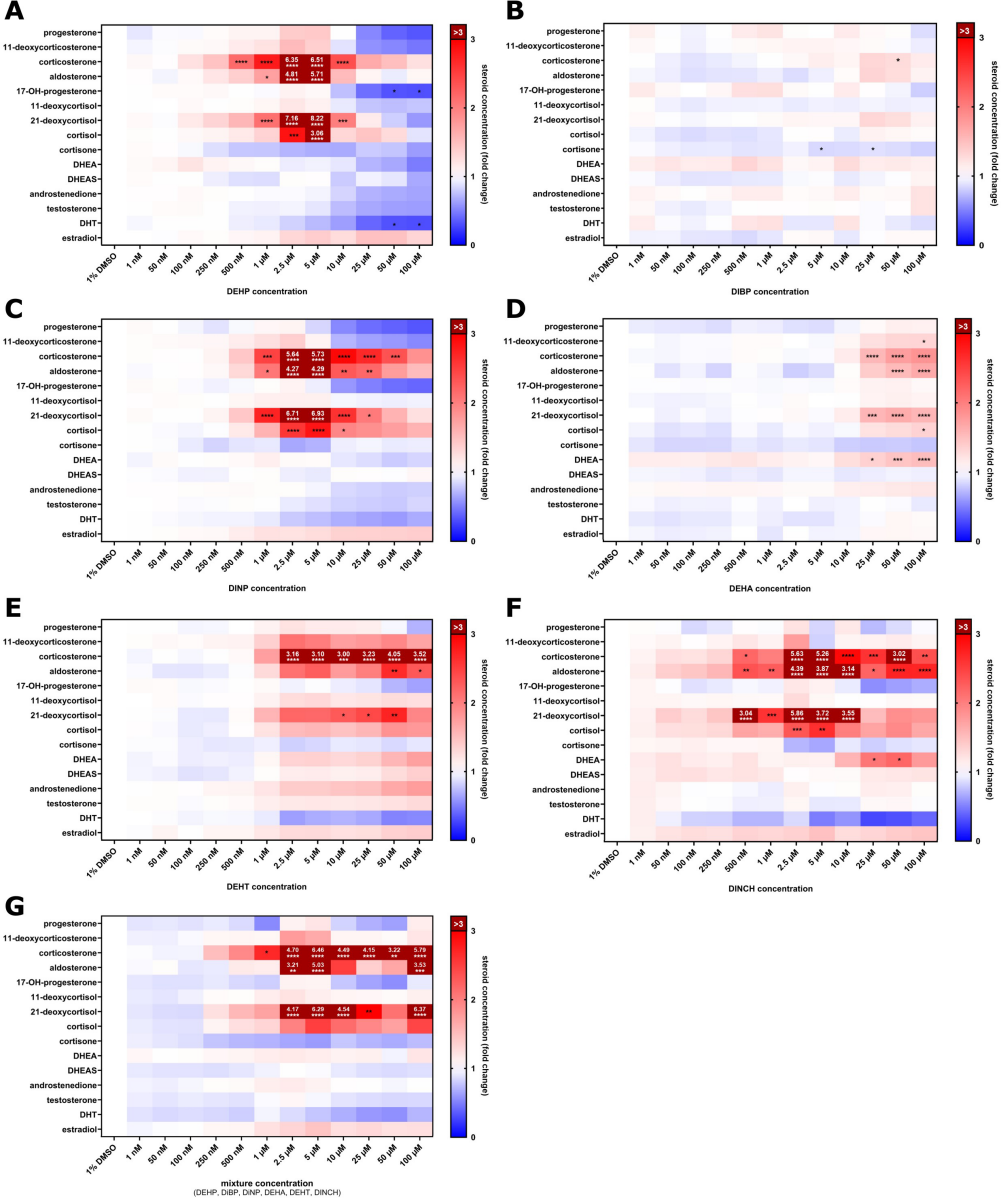
Fold changes in steroid concentrations after 72-hour treatment of NCI-H295 cells with DEHP **(A)**, DiBP **(B)**, DiNP **(C)**, DEHA **(D)**, DEHT **(E)**, DINCH **(F)**, or mixture treatment **(G)**, relative to vehicle-treated cells (1% DMSO). Blue indicates a decrease, and red indicates an increase in steroid concentration. Values exceeding a 3-fold increase are shown in dark red with the respective numeric values. Data represent means from three independent experiments (n = 3). Statistical significance compared to vehicle control: *p≤ 0.05, **p≤ 0.01, ***p≤ 0.001, ****p≤ 0.0001. Full data available in the **Supplementary Table S3**.

DEHP exposure resulted in significant alterations in the steroid profile, with biphasic dose-dependent responses observed for several hormones (**Figure 2A**; full data available in the **Supplementary Table S3**). Corticosterone, aldosterone, 21-deoxycortisol, and cortisol were elevated by 6.51 ± 1.03 (*p ≤* 0.0001), 5.71 ± 0.77 (*p ≤* 0.0001), 8.22 ± 1.01 (*p ≤* 0.0001), and 3.06 ± 0.27 (*p ≤* 0.0001) at 5 µM, respectively. Results after treatment at concentrations >5 µM reapproached the order of control values. Estradiol secretion continuously increased with DEHP concentration to a maximum of 1.48 ± 0.05 at 25 µM (*p* = 0.3171), while several steroids were found to be lowered in comparison to control, e.g., DHT (0.40 ± 0.07 at 25 µM; *p* = 0.1148), progesterone (0.53 ± 0.04 at 25 µM; *p* = 0.3383), or cortisone (0.66 ± 0.12 at 10 µM; *p* = 0.7568).

#### DiBP

*E*xposure to DiBP had only mild effects on steroid excretion (**Figure 2B**). Corticosterone and aldosterone levels were elevated with the highest observed values at 25 µM (1.31 ± 0.11; *p* = 0.1191; 1.33 ± 0.07; *p* = 0.0743), while most other steroids remained unchanged.

#### DiNP

Treatment with DiNP predominantly increased secretion of corticosterone (highest observed levels: 5.73 ± 1.12 at 5 µM; *p ≤* 0.0001), aldosterone (4.29 ± 0.77 at 5 µM; *p ≤* 0.0001), 21-deoxycortisol (6.93 ± 1.32 at 5 µM; *p ≤* 0.0001), and cortisol (2.83 ± 0.39 at 5 µM; *p ≤* 0.0001) (**Figure 2C**). The highest effect was measured at a treatment dose of 5 µM. Higher doses resulted in decreased steroid secretion, e.g., progesterone levels of 0.38 ± 0.14 at 50 µM (*p* = 0.4092).

#### DEHA

Levels of aldosterone and corticosterone increased with the highest observed values of 1.65 ± 0.39 (*p ≤* 0.0001), and 1.84 ± 0.28 (*p ≤* 0.0001) at 100 µM, along with 21-deoxycortisol (1.64 ± 0.39 at 100 µM; *p ≤* 0.0001) (**Figure 2D**). DHEA levels were enhanced to maximum values of 1.54 ± 0.08 at 100 µM (*p ≤* 0.0001).

#### DEHT

DEHT treatment resulted in significantly altered levels of 11-deoxycorticosterone (highest observed levels 2.11 ± 0.16 at 2.5 µM; *p* = 0.1208), corticosterone (3.16 ± 0.30 at 2.5 µM; *p ≤* 0.0001), aldosterone (2.67 ± 0.70 at 50 µM; *p ≤* 0.01), 21-deoxycortisol (2.72 ± 1.76 at 50 µM; *p ≤* 0.01), and cortisol (1.84 ± 0.55 at 50 µM; *p* = 0.3976) (**Figure 2E**). DHT levels decreased in comparison to DMSO-treated controls, with the lowest observed fold change of 0.52 ± 0.15 at 50 µM (*p* = 0.9322).

#### DINCH

Secretion of corticosterone (highest observed fold change: 5.63 ± 0.39 at 2.5 µM; *p ≤* 0.0001), aldosterone (4.39 ± 0.87 at 2.5 µM; *p ≤* 0.0001), 21-deoxycortisol (5.86 ± 0.38 at 2.5 µM; *p ≤* 0.0001), cortisol (2.63 ± 0.06 at 5 µM; *p ≤* 0.001), and estradiol (1.48 ± 0.07 at 5 µM; *p* = 0.8019) were elevated following DINCH treatment (**Figure 2F**). DHT concentrations were lowered up to levels of 0.28 ± 0.06 at 25 µM (*p* = 0.3378) treatment dose.

#### Combinatory mixture

The combination of DEHP, DiBP, DiNP, DEHA, DEHT, and DINCH treatments (“mix”) resulted in pronounced alterations of corticosterone (highest observed fold change: 6.46 ± 0.28 at 5 µM; *p ≤* 0.0001), aldosterone (5.03 ± 0.52 at 5 µM; *p ≤* 0.0001), 21-deoxycortisol (6.29 ± 0.49 at 5 µM; *p ≤* 0.0001), cortisol (2.50 ± 0.07 at 5 µM; *p* = 0.0785), and estradiol (1.37 ± 0.10 at 5 µM; *p* = 0.9912) (**Figure 2G**). Concentrations of cortisone were markedly lowered to a minimum fold change of 0.61 ± 0.04 at 5 µM (*p* = 0.9975). DHT levels decreased to the lowest value of 0.56 ± 0.38 at 50 µM (*p* = 0.9935). Concentrations of other steroid hormones did not show major changes.

### Enzyme activity

The most notable alterations were observed in enzymatic steps catalyzed by steroid 11β-hydroxylase (CYP11B1): the conversion of 11-deoxycorticosterone to corticosterone was significantly enhanced, as indicated by reduced substrate-to-product ratios, e.g., 0.22 ± 0.02, 0.25 ± 0.02, and 0.16 ± 0.01 at 5μµM treatment with DEHP, DiNP, and DINCH (each *p ≤* 0.0001; full data available in the **Supplementary Table S4**). Similarly, the conversion of 17-hydroxyprogesterone to 21-deoxycortisol (catalyzed by CYP11B1) occurred more frequently in treated cells compared to vehicle controls, with the highest observed changes at 5μµM DEHP, DEHT, and the mixture (0.13 ± 0.01, 0.46 ± 0.04, 0.17 ± 0.01; each *p ≤* 0.01).

Regarding CYP17A1 activity, a significant increase in 17,20-lyase-mediated conversion of 17-hydroxyprogesterone to androstenedione was detected, particularly following DEHP and DEHT exposure (e.g., 0.68 ± 0.02 at 2.5 µM; *p ≤* 0.0001), while the 17α-hydroxylase activity of the same enzyme was unaffected.

The ratio referring to the hydroxylation of 17-hydroxyprogesterone to 11-deoxycortisol (CYP21A2) decreased under different exposures (e.g., 0.56 ± 0.06 at 25μµM DEHP; *p ≤* 0.001; 0.61 ± 0.07 at 10μµM DiNP; *p ≤* 0.001; 0.75 ± 0.01 at 2.5μµM DEHT; *p ≤* 0.0001), whereas the subsequent conversion of 21-deoxycortisol to cortisol was impaired, as shown by increased product-to-substrate ratios.

The relative activity of aromatase (CYP19A1), producing estradiol from testosterone, has been slightly increased by DEHP (e.g., 0.58 ± 0.05 at 10 µM; *p* = 0.1286) and DiNP (e.g., 0.61 ± 0.02 at 10 µM; *p* < 0.0001).

In addition, the ratio of cortisol to its inactive metabolite cortisone - reflecting HSD11B2 activity- was consistently elevated across all treatments, suggesting reduced enzymatic function. The strongest changes were observed at 5μµM DEHP (4.48 ± 0.29; *p ≤* 0.0001), DiNP (4.04 ± 0.26; *p ≤* 0.0001), and the mixture (4.12 ± 0.16; *p ≤* 0.01). Furthermore, 5α-reductase (3-oxo-5α-steroid 4-dehydrogenase, *SRD5*) activity, responsible for the conversion of testosterone to the more potent dihydrotestosterone, appeared increasingly inhibited with higher concentrations of DEHP, DEHT, DINCH, and the mixture, e.g., 3.92 ± 0.90 at 25 µM of DINCH (*p ≤* 0.0001).

No consistent changes were seen in the relative activity of CYP11B2, HSD3B2, SULT2A1, or HSD17B3.

### Quantitative real-time PCR

**Figure 3:**
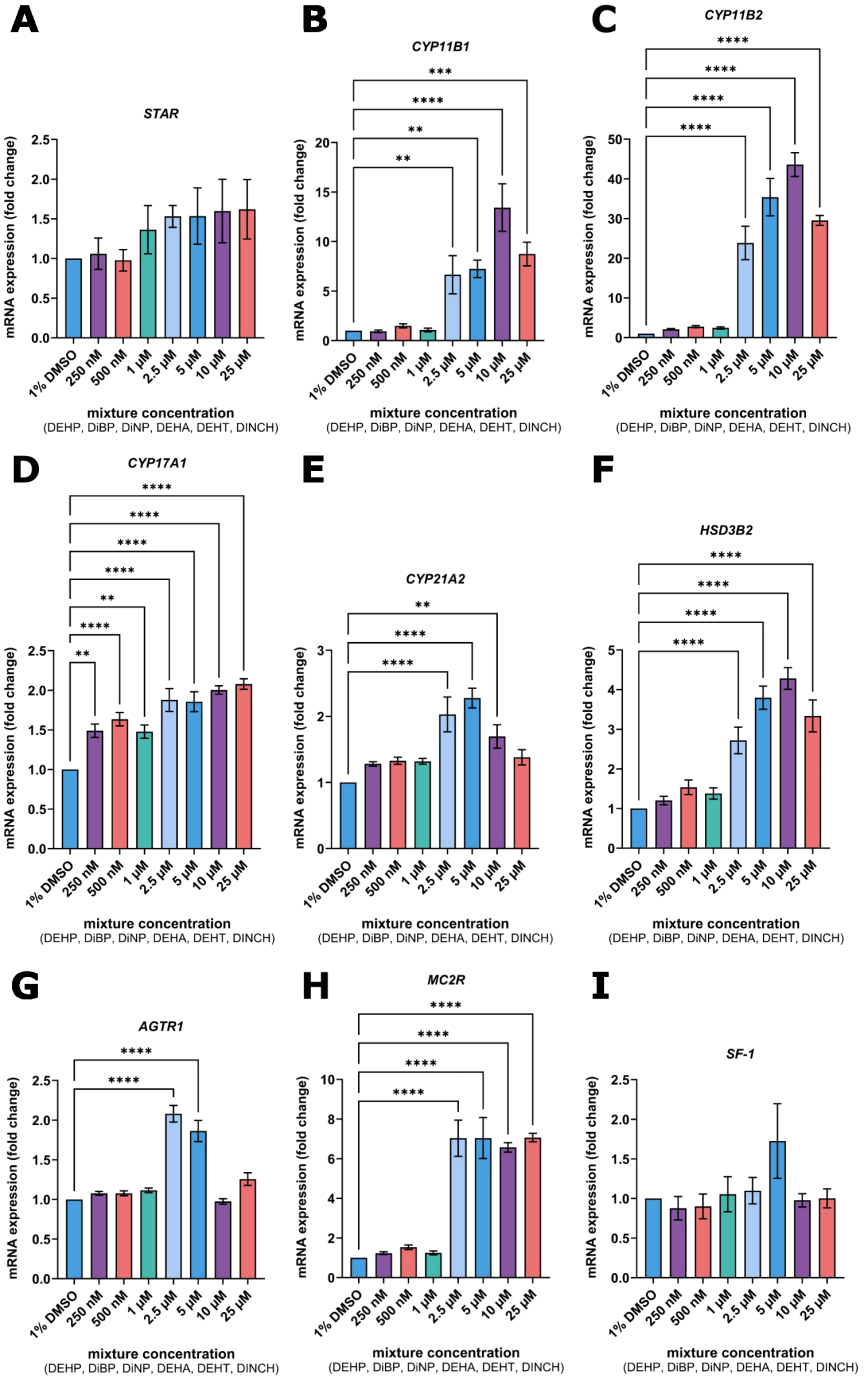
mRNA expression of *STAR***(A)**, *CYP11B1***(B)**, *CYP11B2***(C)**, *CYP17A1***(D)**, *CYP21A2***(E)**, *HSD3B2***(F)**, *AGTR1***(G)**, *MC2R***(H)**, and *SF-1***(I)** in NCI-H295R cells after 72-hour exposure to combinatory treatment (equimolar mixture of DEHP, DiBP, DiNP, DEHA, DEHT, DINCH) at concentrations ranging from 250 nM to 25 µM. Expression levels are shown as fold changes in relation to vehicle control (1% DMSO). Bars represent mean ± standard error of the mean (*SEM*) of n = 3 independent experiments. Statistical significance compared to vehicle control: **p≤ 0.01, ***p≤ 0.001, ****p≤ 0.0001. Full data available in the **Supplementary Table S5**.

Gene expression of essential mediators of steroidogenesis in response to the mixture of DEHP, DiBP, DiNP, DEHA, DEHT, and DINCH was quantified via RT-qPCR (**Figure 3**; full data available in the **Supplementary Table S5**).

Respective treatment resulted in a modest, concentration-dependent elevation of *StAR* mRNA expression compared with the 1% DMSO control (**Figure 3A**). Concentrations ≥ 2.5 µM produced a slight upregulation, e.g.1.53 ± 0.30 at 2.5 µM (*p* = 0.6134). mRNA levels of *CYP11B1* and *CYP11B2* were strongly elevated by treatment with the mixture compared to vehicle control (**Figures 3B, C**). For instance, treatment with 10 µM resulted in 11.82 ± 4.40-fold changes in *CYP11B1* and 44.12 ± 9.77-fold changes in *CYP11B2* expression (both *p ≤* 0.0001). Also, expression of *HSD3B2* was increased by treatment to a maximum fold change at 10 µM (4.26 ± 0.87; *p ≤* 0.0001) (**Figure 3F**). Expression of *CYP17A1* was even affected at nanomolar concentrations, e.g., 1.63 ± 0.24 at 500 nM (*p ≤* 0.0001), with maximum values at 25 µM (2.10 ± 0.18; *p ≤* 0.0001) (**Figure 3D**). mRNA levels of *CYP21A2* were found to be increased by treatment, peaking at 5 µM (2.28 ± 0.42; *p ≤* 0.0001) (**Figure 3E**).

Expression of angiotensin II receptor 1 (*AGTR1*) was significantly elevated by treatment with 2.5 µM (2.08 ± 0.30; *p ≤* 0.0001) and 5 µM (1.86 ± 0.38; *p ≤* 0.0001) (**Figure 3G**). Meanwhile, melanocortin 2 receptor (*MC2R*) expression was markedly increased by doses >2.5 µM, with the maximum observed fold change at 25 µM (7.06 ± 0.48; *p ≤* 0.0001) (**Figure 3H**). mRNA levels of SF-1, however, were not significantly altered by the treatment (**Figure 3I**).

## Discussion

**Figure 4:**
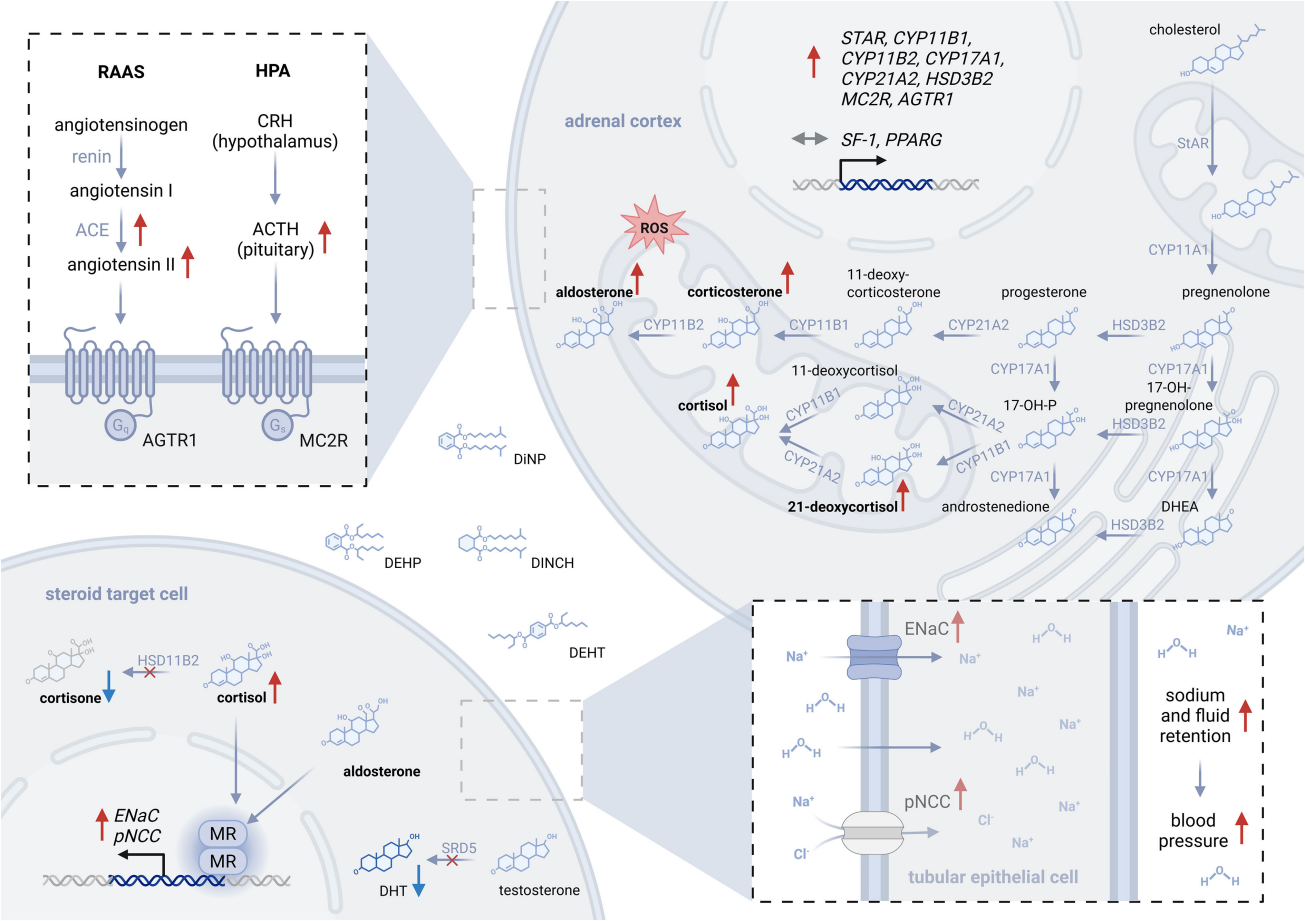
Disruption of adrenal steroidogenesis by phthalates and their substitutes. This schematic summarizes proposed mechanisms by which phthalates and their substitutes may disrupt adrenal steroidogenesis. Activation of both the HPA axis (29, 30, 46) and the RAAS system (52–54) have been reported previously, promoting an activation of AGTR1, MC2R, and consecutively the adrenal steroidogenesis. In the present study, key genes involved in steroid biosynthesis and endocrine regulation—STAR, CYP11B1, CYP17A1, CYP21A2, MC2R, and AGTR1—were upregulated following exposure, as well as the steroid secretion of key adrenal steroids – corticosterone, cortisol, and aldosterone. Relevant inhibition of the HSD11B2, involved in selective activation of MR (31, 48), further increases cortisol availability. 5α-reductase inhibition results in decreased levels of active DHT. These findings align with previous reports and support the hypothesis of aberrant downstream MR activation by cortisol and aldosterone (31). Increased expression of pNCC and ENaC in tubular membranes points to alterations in renal sodium and water retention (31, 32, 50), which may contribute to increased blood pressure risk. Red and blue arrows indicate an increase or decrease in secretion, gene or protein expression, or relative enzymatic activity.

The present *in vitro* study with H295R adrenocortical cells shows that phthalates and their substitutes interfere with steroidogenesis, altering the secretion of key human steroids (**Figure 4**). Concordant results from all experiments suggest an activation of mineralo- and glucocorticoid production, significantly elevating levels of corticosterone, cortisol, 21-deoxycortisol, and aldosterone. For several compounds—including DINCH, DEHP, DEHT, DiNP, and their mixture—these steroid concentrations are increased by >3-fold compared to controls. Substantial changes in surrogates of the relative activity of essential steroidogenic enzymes, including CYP11B1, CYP17A1, and CYP21A2, were observed. Phthalates and their substitutes also strongly induced mRNA expression of *CYP11B2* (up to 44-fold), *CYP11B1* (up to 11-fold), alongside significant elevations of *STAR*, *CYP17A1, CYP21A2*, and *HSD3B2.* As adrenocortical function is regulated by two central endocrine systems—the hypothalamic-pituitary-adrenal (HPA-) axis and the renin-angiotensin-aldosterone system (*RAAS*)—the corresponding adrenal receptors were also examined. While ACTH binds to the melanocortin 2 receptor (*MC2R*), promoting the expression of genes such as *StAR*, *CYP11B1*, *CYP17A1*, and *MC2R* itself, angiotensin II signals through the angiotensin II type 1 receptor (AGTR1), increasing *CYP11B2* and *HSD3B2* expression (45). In this study, *AGTR1* and *MC2R* were upregulated, indicating activation of the related signaling cascades, while *StAR*, the rate-limiting step in steroidogenesis, was also induced. By contrast, *SF-1*, a central transcription factor for steroidogenic genes, was not significantly altered.

### Glucocorticoid pathway and the hypothalamic-pituitary axis

Epidemiological studies have shown a correlation between elevated cortisol in human samples (e.g., hair, urine) and phthalate exposure, particularly in infants, adolescents, and predominantly in females (17–19). These substances have been previously described as neuroendocrine disruptors affecting all components of the HPA-axis, including dysregulation of CRH, ACTH, cortisol, carrier proteins, peripheral steroid receptors, and deactivating enzymes (29, 30, 46). Behavioral effects have been consistently demonstrated in phthalate-treated rodent models, where HPA-axis dysregulation coincided with pronounced anxiety-like phenotypes and impaired stress response (28, 29).

In the present study, treatment with phthalates or non-phthalate substitutes increased cortisol levels while reducing its derivative cortisone. Also, the strong increase in 21-deoxycortisol, an intermediate of an alternative cortisol synthesis route, and the increase of *CYP11B1* expression by 20-fold further support the activation of the glucocorticoid pathway. Consistent with the observed changes in HSD11B2 activity, previous reports indicate that phthalates can inhibit hydroxysteroid dehydrogenases (21, 47), and specifically HSD11B2 in renal, ovarian, and testicular tissue (48, 49), increasing cortisol-cortisone ratio (21, 31). HSD11B2 is a key enzyme in aldosterone and cortisol target tissues (e.g., kidney, gastrointestinal tract), converting cortisol to cortisone to prevent inappropriate mineralocorticoid receptor (*MR*) activation. Impaired cortisol deactivation through phthalates and their substitutes therefore results in an accumulation of active cortisol and potentially consequent aberrant MR activation. *In vivo*, such MR activation by cortisol driven by phthalate-induced HSD11B2 inhibition has been associated with hypertensive phenotypes (26), providing a plausible mechanistic link between the present *in vitro* findings and reported cardiovascular outcomes.

### RAAS and risk of hypertension

The present results indicate that with increased secretion of mineralocorticoids and/or by inhibition of HSD11B1/2, more active ligands on the MR are present following phthalate and substitute treatment. In epidemiological association studies, DEHP concentrations were, in fact, higher in hypertensive than normotensive infants (26). Furthermore, removing DEHP from medical equipment (e.g., IV fluids) reduced the incidence of hypertension in patients of neonatal care units, whereas reintroduction of DEHP-containing products led to an increase in cases with hypertension comparable to pre-interventional levels (50). Epidemiological evidence suggests that differences between high-molecular-weight phthalates, such as DEHP, DiNP, DiDP, and low-molecular-weight phthalates exist, as the latter have no significant effect on blood pressure [e.g., DiBP (51)], which might be reflected in the present study by their small impact on adrenal mineralocorticoids.

The hypertensive effect of DEHP was repeatedly confirmed in *in vivo* exposure studies, as well as epidemiological cohort and cross-sectional studies that measured urinary DEHP metabolites and found significantly higher blood pressure levels in exposed populations (26, 51–54). Moreover, enhanced expression of RAAS components, including renin (52), angiotensin-converting enzyme (*ACE)*, angiotensin II, and AGTR1 (53, 54) has been reported. Similar effects have been detected for DiNP exposure (54). Furthermore, urine levels of DEHP were associated with altered fluid homeostasis (32), increased sodium retention, and elevated expression of renal sodium channels, including epithelial sodium channel (*ENaC*), and phosphorylated sodium chloride cotransporter (*pNCC*), being indicators of MR activation (31). Additionally, prenatal exposure studies show an inhibitory effect of DEHP on the expression of MR, while MR-associated genes (e.g., ENaC) were increased in kidney tissue (49).

This study is the first to reveal that the adrenal compounds of the RAAS system, namely *AGTR1*, steroidogenic enzymes (*StAR, HSD3B2, CYP11B1, CYP11B2*), and the respective mineralocorticoids are affected by phthalates and their substitutes. Therefore, the reported direct effects on adrenal steroidogenesis, including aldosterone secretion, might explain the underlying cause of plasticizer-induced hypertension.

Additionally, phthalates may act as selective peroxisome proliferator-activated receptor gamma (*PPARγ*) modulators in steroidogenic tissue (55), as previously suggested for monoethylhexyl phthalate (*MEHP*), including selective coactivator recruitment (e.g., PPARγ coactivator 1-alpha) and potentially enhancing *CYP11B1* and *CYP11B2* expression, as well as aldosterone secretion (56).

Further cellular stress-mediated mechanisms such as interference with cAMP/PKA/SF-1 signaling (45), oxidative stress, or mitochondrial dysfunction may contribute to enzyme induction and altered hormone production. Phthalates have been linked to oxidative stress, which is often discussed in connection with disrupted steroidogenesis. However, available evidence indicates that phthalates induce significant oxidative stress only at concentrations exceeding 100 µM in steroidogenic cell models (23, 57–61). For instance, while DBP has been shown to induce oxidative stress concomitant with steroid alterations in H295R cells, these effects occurred only at concentrations exceeding those used in the present study (23). Therefore, while oxidative stress is a plausible mechanism, it is unlikely to account for the steroidogenic changes observed here at lower, sub-cytotoxic phthalate concentrations.

Phthalate exposure resulted in biphasic, non-monotonic dose–response curves with maximal effects between 1 and 10 µM. Non-monotonicity is well documented for several phthalates and can arise from receptor selectivity, receptor deregulation or desensitization, incipient cytotoxicity, autocrine feedback, pathway-level feedback inhibition, or dose-dependent activation of adaptive stress responses (62). In adrenal steroidogenesis specifically, low-dose phthalate exposure may enhance upstream signaling or enzyme activity (e.g., increased MC2R/ACTH responsiveness or upregulation of CYP11B1/CYP11B2), whereas higher concentrations may induce compensatory downregulation, reduced substrate availability, or early cytoprotective stress responses that attenuate steroid output. These mechanisms are consistent with the peak effects observed at 2.5–5 µM and the subsequent decline at higher concentrations.

### Sex steroids

The anti-androgenic effect of phthalates, especially DEHP, and their involvement in the development of reproductive impairment have already been described (14). Epidemiological data showed an inverse correlation of phthalate metabolites with androstenedione, DHEA, and testosterone, and a positive correlation with estradiol levels (63). The presented results confirm and extend this knowledge by showing that prominent adrenal androgens, DHEA and androstenedione, are impaired by phthalates, with the most pronounced changes observed following DEHP treatment. Additionally, relative 5α-reductase activity was significantly inhibited by DEHP, DEHT, and DINCH, reaching only ~25% of control levels. This inhibition results in decreased DHT production and may thereby represent a potential mechanism underlying the pathogenesis of phthalate-associated hypospadias (64). In contrast, *non*-phthalate plasticizers, namely DEHA, DEHT, and DINCH, resulted in elevated levels of androstenedione and DHEA, suggesting an androgenic effect on H295R cells.

To simulate realistic co-exposure conditions, cells were treated with an equimolar mixture of all six compounds. This combined treatment altered key steroid hormones (e.g., progesterone, corticosterone, 21-deoxycortisol, cortisol) - even at doses where individual substances showed no effect, indicating supra-additive disruption. This observation aligns with European biomonitoring data, where mixture risk assessments revealed health risks in 17% of individuals that would have been missed by single-compound evaluations (41). Thus, mixture effects should be routinely considered in EDC testing to better reflect real-world combined exposures.

These results underscore the steroidogenesis- and thus endocrine-disrupting potential of both classical phthalates and commonly used substitutes (e.g., terephthalates, cyclohexanoates). Further, the urgent need to reconsider toxicological assessment approaches and to adapt *in vitro* test settings, including the use of expanded steroid panels, is highlighted. If confirmed *in vivo*, these findings could have important implications for the understanding and management of steroid-linked disorders and environmental adrenal disruption, warranting further evaluation.

## Limitations

The chosen study design has several limitations. First, the *in vitro* screening lacks the possibility to replicate physiological stimulatory or inhibitory feedback mechanisms regulating steroid secretion. Second, biological tissues are exposed to potentially disrupting chemicals over extended periods, while laboratory testing is limited to acute one-time exposures, neglecting processes such as ingestion, absorption, accumulation, storage, and excretion. Third, gene expression analysis was performed only for the mixture treatment to capture the most relevant changes. A further limitation is that only a single adrenocortical carcinoma cell line was employed in this work. NCI-H295R cells, which are the only available human *in vitro* model capable of synthesizing the full spectrum of clinically relevant adrenal steroids and expressing the complete panel of steroidogenic enzymes, were used. Other adrenal-derived cell lines lack these properties, though NCI-H295R is widely recognized as the preferred model for endocrine-disruptor screening and is recommended by regulatory guidelines for evaluating effects on steroidogenesis (42, 43).

## Conclusion

The present study provides compelling evidence that phthalates and their emerging substitutes disrupt adrenal steroidogenesis, notably enhancing the synthesis of cortisol, aldosterone, and related intermediates at non-cytotoxic concentrations. Transcriptional changes in key steroidogenic enzymes, particularly CYP11B1 and CYP11B2, together with alterations in regulatory receptors, may provide a mechanistic basis for the adverse outcomes observed *in vivo*, especially secondary hypertension in humans. Importantly, the data show that substitute plasticizers, such as DEHT and DINCH, induce endocrine effects comparable to or exceeding those of restricted phthalates, challenging the notion of their reduced toxicity. Moreover, additive effects were observed when a mixture of these substances was applied. Given the widespread human exposure to complex plasticizer mixtures, the current findings highlight the need for mixture-based risk assessments and regulatory strategies that reflect real-world exposure scenarios. Future studies should further explore the systemic consequences of adrenal disruption *in vivo* and assess long-term cardiovascular and metabolic risks across developmental windows.

## Data Availability

The datasets presented in this study can be found in online repositories. The names of the repository/repositories and accession number(s) can be found in the article/**Supplementary Material**.
