# Supplementary material for: Disruptive effects of phthalates and their substitutes on adrenal steroidogenesis
Source: Front Endocrinol (Lausanne). 2026 Jan 14;16:1734184. doi: 10.3389/fendo.2025.1734184 (PMC12848149; doi:10.3389/fendo.2025.1734184)
Supplement: Supplementary file 1 [file DataSheet1.docx]

**Supplementary Material S1.** List of abbreviations used in the manuscript.

17-OHP – 17-hydroxyprogesterone

ACE – angiotensin-converting enzyme

ACTB – beta-actin

ACTH – adrenocorticotropic hormone

AGTR1 – angiotensin II receptor type 1

AR – androgen receptor

BBP – butyl benzyl phthalate

CRH – corticotropin-releasing hormone

CYP11B1 – cytochrome P450 family 11 subfamily B member 1 (steroid 11β-hydroxylase)

CYP11B2 – cytochrome P450 family 11 subfamily B member 2 (aldosterone synthase)

CYP17A1 – cytochrome P450 family 17 subfamily A member 1 (17α-hydroxylase/17,20-lyase)

CYP21A2 – cytochrome P450 family 21 subfamily A member 2 (21-hydroxylase)

DBP – dibutyl phthalate

DCHP – dicyclohexyl phthalate

DEHA – di(2-ethylhexyl) adipate

DEHP – di(2-ethylhexyl) phthalate

DEHT – di(2-ethylhexyl) terephthalate

DHEA – dehydroepiandrosterone

DHEAS – dehydroepiandrosterone sulfate

DHT – dihydrotestosterone

DiBP – diisobutyl phthalate

DINCH – 1,2-cyclohexane dicarboxylic acid diisononyl ester (*diisononyl cyclohexane-1,2-dicarboxylate*)

DiNP – diisononyl phthalate

DMSO – dimethyl sulfoxide

ECHA – European Chemicals Agency

EFSA – European Food Safety Authority

ENaC – epithelial sodium channel

ER – estrogen receptor

FBS – fetal bovine serum

FDA – U.S. Food and Drug Administration

HPA – hypothalamic–pituitary–adrenal (axis)

HPV – high production volume

HSD11B2 – 11β-hydroxysteroid dehydrogenase type 2

HSD3B2 – 3β-hydroxysteroid dehydrogenase type 2

ITS – insulin–transferrin–selenium supplement (not IST)

MC2R – melanocortin 2 receptor (ACTH receptor)

MEHA – mono(2-ethylhexyl) adipate (metabolite of DEHA)

MEHHP – mono(2-ethyl-5-hydroxyhexyl) phthalate (metabolite of DEHP)

MEHP – mono(2-ethylhexyl) phthalate (primary metabolite of DEHP)

MEHT – mono(2-ethylhexyl) terephthalate (metabolite of DEHT)

MEOHP – mono(2-ethyl-5-oxohexyl) phthalate (metabolite of DEHP)

MiBP – mono-isobutyl phthalate

MINCH – monoisononyl cyclohexane-1,2-dicarboxylate (metabolite of DINCH)

MiNP – monoisononyl phthalate

MR – mineralocorticoid receptor

OECD – Organisation for Economic Co-operation and Development

OH-MiBP – hydroxy-mono-isobutyl phthalate (oxidized metabolite)

OH-MINCH – hydroxy-monoisononyl cyclohexane-1,2-dicarboxylate

OH-MiNP – hydroxy-monoisononyl phthalate

PBS – phosphate-buffered saline

PKA – protein kinase A

pNCC – phosphorylated sodium chloride cotransporter

PPARγ – peroxisome proliferator–activated receptor gamma

PVC – polyvinyl chloride

RAAS – renin–angiotensin–aldosterone system

REACH – Registration, Evaluation, Authorisation and Restriction of Chemicals (EU regulation)

SF-1 – steroidogenic factor-1 (NR5A1)

SPPARM – selective PPAR modulator

STAR – steroidogenic acute regulatory protein

SVHC – substance of very high concern

TSCA – Toxic Substances Control Act (U.S.)
