# Supplementary material for: Disruptive effects of phthalates and their substitutes on adrenal steroidogenesis
Source: Front Endocrinol (Lausanne). 2026 Jan 14;16:1734184. doi: 10.3389/fendo.2025.1734184 (PMC12848149; doi:10.3389/fendo.2025.1734184)
Supplement: Supplementary file 2 [file DataSheet2.docx]

**Supplementary Material S2.** List of used primer-probe sequences in gene expression analysis.

| **Housekeeping gene** | **Primer sequence** |
| --- | --- |
| ACTB | ACTB-F: AATGAAGATCAAGATCATTGCTCCT ACTB-R: ACATCTGCTGGAAGGTGGAC ACTB-IN: AGCGCAAGTACTCCGTGTGGATC |
| **Gene of interest** | **TaqMan Gene Expression Assay-ID** |
| CYP11B1 | Hs01596404_m1 |
| CYP11B2 | Hs01597732_m1 |
| CYP17A1 | Hs01124136_m1 |
| CYP21A2 | Hs00365736_m1 |
| HSD3B2 | Hs00605123_m1 |
| StAR | Hs00986559_g1 |
| SF-1 | Hs00124206_m1 |
| MC2R | Hs00300820_s1 |
| AGTR1 | Hs05043708_s1 |
