# Supplementary material for: Disruptive effects of phthalates and their substitutes on adrenal steroidogenesis
Source: Front Endocrinol (Lausanne). 2026 Jan 14;16:1734184. doi: 10.3389/fendo.2025.1734184 (PMC12848149; doi:10.3389/fendo.2025.1734184)
Supplement: Supplementary file 3 [file DataSheet3.docx]

**Supplementary Material S3.** Steroid concentrations after treatment, measured by LCMS/MS. Data are presented as fold-changes compared to vehicle-treated controls (n = 3; mean ± SD**).** Respective *p*-values are presented for each data point, while statistically significant values are marked in bold and grey background.

| **DEHP** | **1 nM** | **50 nM** | **100 nM** | **250 nM** | **500 nM** | **1 µM** | **2.5 µM** | **5 µM** | **10 µM** | **25 µM** | **50 µM** | **100 µM** |
| --- | --- | --- | --- | --- | --- | --- | --- | --- | --- | --- | --- | --- |
| **progesterone** | 0.91±0.02  (*p*>0.9999) | 0.98±0.07  (*p*>0.9999) | 0.97±0.01  (*p*>0.9999) | 1.03±0.06  (*p*>0.9999) | 1.08±0.07  (*p*>0.9999) | 1.09±0.08  (*p*>0.9999) | 1.39±0.07  (*p*=0.5740) | 1.42±0.05  (*p*=0.4964) | 1.01±0.22  (*p*>0.9999) | 0.53±0.04  (*p*=0.3383) | 0.39±0.07  (*p*=0.0994) | 0.33±0.06  (*p*=0.0556) |
| **11-deoxycorticosterone** | 0.96±0.15  (*p*>0.9999) | 0.99±0.10  (*p*>0.9999) | 1.03±0.07  (*p*>0.9999) | 1.05±0.06  (*p*>0.9999) | 1.15±0.11  (*p*=0.9995) | 1.22±0.12  (*p*=0.9840) | 1.53±0.11  (*p*=0.2178) | 1.38±0.08  (*p*=0.6056) | 0.89±0.05  (*p*>0.9999) | 0.57±0.03  (*p*=0.4510) | 0.53±0.04  (*p*=0.3417) | 0.46±0.01  (*p*=0.2009) |
| **corticosterone** | 1.05±0.06  (*p*>0.9999) | 1.01±0.05  (*p*>0.9999) | 1.23±0.07  (*p*=0.9737) | 1.46±0.35  (*p*=0.3626) | **2.16±0.99**  **(*p*<0.0001)** | **2.79±1.46**  **(*p*<0.0001)** | **6.35±1.08**  **(*p*<0.0001)** | **6.51±1.03**  **(*p*<0.0001)** | **2.37±1.04**  **(*p*<0.0001)** | 1.65±0.32  (*p*=0.0646) | 1.50±0.38  (*p*=0.2831) | 1.24±0.32  (*p*=0.9691) |
| **aldosterone** | 1.01±0.02  (*p*>0.9999) | 0.95±0.02  (*p*>0.9999) | 1.04±0.04  (*p*>0.9999) | 1.20±0.13  (*p*=0.9937) | 1.38±0.28  (*p*=0.6286) | **1.74±0.65**  **(*p*=0.0240)** | **4.87±0.51**  **(*p*<0.0001)** | **5.71±0.77**  **(*p*<0.0001)** | 1.53±0.57  (*p*=0.2138) | 1.08±0.08  (*p*>0.9999) | 0.93±0.11  (*p*>0.9999) | 1.07±0.22  (*p*>0.9999) |
| **17-OH-progesterone** | 1.00±0.02  (*p*>0.9999) | 1.03±0.02  (*p*>0.9999) | 1.03±0.00  (*p*>0.9999) | 1.05±0.03  (*p*>0.9999) | 1.00±0.07  (*p*>0.9999) | 0.98±0.13  (*p*>0.9999) | 1.10±0.06  (*p*>0.9999) | 1.05±0.04  (*p*>0.9999) | 0.75±0.17  (*p*=0.9570) | 0.43±0.05  (*p*=0.1483) | **0.32±0.05**  **(*p*=0.0488)** | **0.31±0.10**  **(*p*=0.0441)** |
| **11-deoxycortisol** | 1.00±0.07  (*p*>0.9999) | 1.00±0.06  (*p*>0.9999) | 1.04±0.06  (*p*>0.9999) | 1.01±0.05  (*p*>0.9999) | 1.02±0.08  (*p*>0.9999) | 1.07±0.07  (*p*>0.9999) | 1.18±0.05  (*p*=0.9967) | 1.11±0.04  (*p*>0.9999) | 0.92±0.08  (*p*>0.9999) | 0.77±0.02  (*p*=0.9740) | 0.74±0.03  (*p*=0.9468) | 0.77±0.10  (*p*=0.9760) |
| **21-deoxycortisol** | 1.05±0.11  (*p*>0.9999) | 0.99±0.14  (*p*>0.9999) | 1.17±0.00  (*p*=0.9987) | 1.33±0.17  (*p*=0.7791) | 1.57±0.24  (*p*=0.1561) | **2.11±0.55**  **(*p*<0.0001)** | **7.16±0.98**  **(*p*<0.0001)** | **8.22±1.01**  **(*p*<0.0001)** | **2.00±0.56**  **(*p*=0.0005)** | 1.16±0.20  (*p*=0.9994) | 0.81±0.14  (*p*=0.9954) | 0.60±0.12  (*p*=0.5458) |
| **cortisol** | 1.02±0.03  (*p*>0.9999) | 1.00±0.01  (*p*>0.9999) | 1.05±0.03  (*p*>0.9999) | 1.08±0.06  (*p*>0.9999) | 1.17±0.09  (*p*=0.9983) | 1.34±0.09  (*p*=0.7341) | **2.86±0.21**  **(*p*<0.0001)** | **3.06±0.27**  **(*p*<0.0001)** | 1.31±0.12  (*p*=0.8290) | 1.45±0.14  (*p*=0.4096) | 1.28±0.07  (*p*=0.9138) | 0.89±0.03  (*p*>0.9999) |
| **cortisone** | 1.03±0.01  (*p*>0.9999) | 1.01±0.05  (*p*>0.9999) | 0.97±0.07  (*p*>0.9999) | 0.87±0.09  (*p*=0.9999) | 0.78±0.21  (*p*=0.9823) | 0.78±0.19  (*p*=0.9806) | 0.71±0.04  (*p*=0.8774) | 0.68±0.03  (*p*=0.8138) | 0.66±0.12  (*p*=0.7568) | 0.79±0.04  (*p*=0.9906) | 0.77±0.04  (*p*=0.9741) | 0.61±0.11  (*p*=0.5837) |
| **DHEA** | 1.04±0.11  (*p*>0.9999) | 1.07±0.14  (*p*>0.9999) | 1.21±0.13  (*p*=0.9879) | 1.09±0.15  (*p*>0.9999) | 1.05±0.15  (*p*>0.9999) | 1.02±0.13  (*p*>0.9999) | 0.93±0.04  (*p*>0.9999) | 0.95±0.05  (*p*>0.9999) | 0.90±0.13  (*p*>0.9999) | 0.70±0.05  (*p*=0.8725) | 0.66±0.04  (*p*=0.7416) | 0.49±0.08  (*p*=0.2459) |
| **DHEAS** | 1.03±0.06  (*p*>0.9999) | 0.99±0.04  (*p*>0.9999) | 0.99±0.04  (*p*>0.9999) | 0.93±0.05  (*p*>0.9999) | 0.87±0.14  (*p*>0.9999) | 0.87±0.11  (*p*=0.9999) | 1.02±0.06  (*p*>0.9999) | 1.01±0.05  (*p*>0.9999) | 0.79±0.11  (*p*=0.9897) | 0.94±0.05  (*p*>0.9999) | 0.90±0.05  (*p*>0.9999) | 0.66±0.13  (*p*=0.7515) |
| **androstenedione** | 1.01±0.05  (*p*>0.9999) | 1.01±0.04  (*p*>0.9999) | 1.04±0.01  (*p*>0.9999) | 1.02±0.10  (*p*>0.9999) | 0.99±0.10  (*p*>0.9999) | 0.98±0.09  (*p*>0.9999) | 0.98±0.01  (*p*>0.9999) | 0.95±0.00  (*p*>0.9999) | 0.83±0.08  (*p*=0.9983) | 0.70±0.05  (*p*=0.8557) | 0.66±0.04  (*p*=0.7575) | 0.64±0.06  (*p*=0.6929) |
| **testosterone** | 1.00±0.04  (*p*>0.9999) | 1.02±0.03  (*p*>0.9999) | 1.04±0.01  (*p*>0.9999) | 0.99±0.07  (*p*>0.9999) | 0.95±0.09  (*p*>0.9999) | 0.94±0.06  (*p*>0.9999) | 0.96±0.02  (*p*>0.9999) | 0.90±0.02  (*p*>0.9999) | 0.74±0.08  (*p*=0.9361) | 0.66±0.05  (*p*=0.7331) | 0.62±0.06  (*p*=0.6265) | 0.61±0.12  (*p*=0.5980) |
| **DHT** | 0.96±0.01  (*p*>0.9999) | 1.01±0.01  (*p*>0.9999) | 1.00±0.01  (*p*>0.9999) | 0.99±0.03  (*p*>0.9999) | 0.93±0.07  (*p*>0.9999) | 0.91±0.07  (*p*>0.9999) | 0.82±0.02  (*p*=0.9973) | 0.73±0.02  (*p*=0.9228) | 0.62±0.16  (*p*=0.6090) | 0.40±0.07  (*p*=0.1148) | **0.32±0.06**  **(*p*=0.0482)** | **0.30±0.13**  **(*p*=0.0396)** |
| **estradiol** | 1.00±0.07  (*p*>0.9999) | 0.99±0.04  (*p*>0.9999) | 1.04±0.02  (*p*>0.9999) | 1.02±0.09  (*p*>0.9999) | 1.06±0.09  (*p*>0.9999) | 1.09±0.09  (*p*>0.9999) | 1.31±0.02  (*p*=0.8307) | 1.39±0.04  (*p*=0.5814) | 1.26±0.11  (*p*=0.9337) | 1.48±0.05  (*p*=0.3171) | 1.47±0.08  (*p*=0.3378) | 1.32±0.12  (*p*=0.8013) |

| **DiBP** | **1 nM** | **50 nM** | **100 nM** | **250 nM** | **500 nM** | **1 µM** | **2.5 µM** | **5 µM** | **10 µM** | **25 µM** | **50 µM** | **100 µM** |
| --- | --- | --- | --- | --- | --- | --- | --- | --- | --- | --- | --- | --- |
| **progesterone** | 1.12±0.15  (*p*=0.8614) | 0.99±0.14  (*p*>0.9999) | 0.95±0.16  (*p*=0.9977) | 1.00±0.18  (*p*>0.9999) | 1.16±0.26  (*p*=0.9423) | 1.21±0.29  (*p*=0.8847) | 1.02±0.02  (*p*=0.7285) | 1.00±0.04  (*p*>0.9999) | 1.16±0.36  (*p*=0.9868) | 1.04±0.05  (*p*=0.8132) | 1.02±0.08  (*p*=0.9989) | 0.86±0.16  (*p*=0.8122) |
| **11-deoxycorticosterone** | 1.05±0.07  (*p*=0.8723) | 0.99±0.11  (*p*>0.9999) | 0.94±0.16  (*p*=0.9935) | 0.99±0.13  (*p*>0.9999) | 1.03±0.13  (*p*=0.9996) | 1.07±0.12  (*p*=0.9587) | **1.09±0.01**  **(*p*=0.0120)** | **1.09±0.01**  **(*p*=0.0357)** | 1.06±0.20  (*p*=0.9987) | 1.04±0.03  (*p*=0.6192) | 0.98±0.05  (*p*=0.9865) | 0.93±0.11  (*p*=0.9178) |
| **corticosterone** | 1.00±0.04  (*p*>0.9999) | 0.93±0.16  (*p*=0.9898) | 0.89±0.21  (*p*=0.9660) | 0.92±0.16  (*p*=0.9715) | 0.96±0.07  (*p*=0.9071) | 1.00±0.06  (*p*>0.9999) | 1.05±0.05  (*p*=0.7418) | 1.13±0.04  (*p*=0.1535) | 1.07±0.13  (*p*=0.9542) | 1.31±0.11  (*p*=0.1991) | **1.27±0.04**  **(*p*=0.0352)** | 1.15±0.04  (*p*=0.0914) |
| **aldosterone** | 0.96±0.04  (*p*=0.7614) | 0.93±0.19  (*p*=0.9949) | 0.87±0.23  (*p*=0.9543) | 0.89±0.19  (*p*=0.9488) | 0.90±0.06  (*p*=0.4443) | 0.93±0.12  (*p*=0.9550) | 0.87±0.09  (*p*=0.5173) | 0.93±0.09  (*p*=0.8830) | 1.02±0.17  (*p*>0.9999) | 1.33±0.07  (*p*=0.0743) | 1.26±0.07  (*p*=0.1171) | 1.10±0.03  (*p*=0.1868) |
| **17-OH-progesterone** | 1.16±0.14  (*p*=0.6438) | 1.05±0.15  (*p*=0.9964) | 1.00±0.15  (*p*>0.9999) | 1.06±0.14  (*p*=0.9896) | 1.24±0.27  (*p*=0.7841) | 1.25±0.28  (*p*=0.7906) | 1.04±0.03  (*p*=0.6561) | 1.01±0.03  (*p*>0.9999) | 1.16±0.27  (*p*=0.9509) | 1.01±0.06  (*p*>0.9999) | 0.96±0.13  (*p*=0.9971) | 0.81±0.17  (*p*=0.6582) |
| **11-deoxycortisol** | 1.02±0.05  (*p*=0.9956) | 0.94±0.12  (*p*=0.9838) | 0.91±0.17  (*p*=0.9559) | 0.94±0.15  (*p*=0.9947) | 0.95±0.11  (*p*=0.9835) | 0.95±0.10  (*p*=0.9839) | 0.92±0.04  (*p*=0.3555) | 0.94±0.08  (*p*=0.8993) | 0.93±0.15  (*p*=0.9813) | 0.95±0.03  (*p*=0.4195) | 0.93±0.06  (*p*=0.6448) | 0.94±0.15  (*p*=0.9948) |
| **21-deoxycortisol** | 1.04±0.10  (*p*=0.9882) | 0.99±0.15  (*p*>0.9999) | 0.96±0.17  (*p*>0.9999) | 0.99±0.15  (*p*>0.9999) | 1.01±0.07  (*p*>0.9999) | 1.03±0.08  (*p*=0.9872) | 1.08±0.04  (*p*=0.3532) | 1.06±0.02  (*p*=0.1894) | 1.06±0.11  (*p*=0.9716) | 1.31±0.16  (*p*=0.3336) | 1.25±0.12  (*p*=0.3123) | 1.11±0.33  (*p*=0.9981) |
| **cortisol** | 0.95±0.02  (*p*=0.1808) | 0.89±0.14  (*p*=0.8860) | 0.86±0.19  (*p*=0.8615) | 0.88±0.15  (*p*=0.8644) | 0.87±0.04  (*p*=0.1419) | 0.90±0.06  (*p*=0.4155) | 1.01±0.07  (*p*>0.9999) | 1.01±0.03  (*p*=0.9955) | 0.92±0.09  (*p*=0.8105) | 1.10±0.11  (*p*=0.7705) | 1.07±0.08  (*p*=0.7811) | 1.03±0.07  (*p*=0.9897) |
| **cortisone** | 0.98±0.01  (*p*=0.3509) | 0.94±0.09  (*p*=0.8886) | 0.91±0.12  (*p*=0.8899) | 0.92±0.12  (*p*=0.9149) | 0.89±0.03  (*p*=0.1278) | 0.90±0.04  (*p*=0.2006) | 0.95±0.05  (*p*=0.6734) | **0.85±0.02**  **(*p*=0.0247)** | 0.85±0.05  (*p*=0.1732) | **0.88±0.01**  **(*p*=0.0141)** | 0.86±0.08  (*p*=0.3604) | 0.82±0.14  (*p*=0.5601) |
| **DHEA** | 1.13±0.11  (*p*=0.6521) | 1.20±0.09  (*p*=0.2815) | 1.14±0.08  (*p*=0.4191) | **1.15±0.02**  **(*p*=0.0205)** | 1.33±0.14  (*p*=0.2539) | 1.23±0.13  (*p*=0.3944) | 1.05±0.07  (*p*=0.8882) | 1.09±0.03  (*p*=0.1529) | 1.27±0.13  (*p*=0.3040) | 1.14±0.02  (*p*=0.0529) | 1.16±0.07  (*p*=0.2594) | 1.12±0.21  (*p*=0.9545) |
| **DHEAS** | 0.98±0.03  (*p*=0.9020) | 0.93±0.07  (*p*=0.7513) | 0.89±0.14  (*p*=0.8422) | 0.90±0.11  (*p*=0.7650) | 0.91±0.09  (*p*=0.7385) | 0.91±0.05  (*p*=0.3920) | 0.97±0.04  (*p*=0.9032) | 0.99±0.11  (*p*>0.9999) | 0.91±0.06  (*p*=0.5481) | 0.95±0.02  (*p*=0.3207) | 0.99±0.09  (*p*>0.9999) | 1.09±0.18  (*p*=0.9794) |
| **androstenedione** | 1.09±0.06  (*p*=0.5351) | 1.04±0.06  (*p*=0.8674) | 1.01±0.06  (*p*>0.9999) | 1.05±0.06  (*p*=0.8118) | 1.14±0.16  (*p*=0.7921) | 1.15±0.17  (*p*=0.8129) | 1.03±0.02  (*p*=0.6231) | **1.05±0.01**  **(*p*=0.0341)** | 1.15±0.18  (*p*=0.8345) | 1.04±0.03  (*p*=0.4949) | 1.05±0.02  (*p*=0.3134) | 1.22±0.19  (*p*=0.6295) |
| **testosterone** | 1.05±0.07  (*p*=0.8977) | 0.94±0.11  (*p*=0.9634) | 0.91±0.15  (*p*=0.9528) | 0.95±0.14  (*p*=0.9973) | 1.01±0.14  (*p*>0.9999) | 1.01±0.12  (*p*>0.9999) | 1.01±0.04  (*p*=0.9988) | 1.02±0.01  (*p*=0.3327) | 1.01±0.16  (*p*>0.9999) | 0.98±0.03  (*p*=0.8688) | 1.01±0.09  (*p*>0.9999) | 1.22±0.32  (*p*=0.8945) |
| **DHT** | 1.13±0.16  (*p*=0.8150) | 0.98±0.19  (*p*>0.9999) | 0.95±0.21  (*p*=0.9997) | 0.99±0.22  (*p*>0.9999) | 1.20±0.35  (*p*=0.9525) | 1.23±0.41  (*p*=0.9535) | 0.91±0.03  (*p*=0.1790) | **0.90±0.01**  **(*p*=0.0282)** | 1.20±0.46  (*p*=0.9857) | 0.98±0.04  (*p*=0.9772) | 0.92±0.07  (*p*=0.6482) | 0.87±0.21  (*p*=0.9326) |
| **estradiol** | 0.95±0.01  (*p*=0.0741) | 0.95±0.06  (*p*=0.8418) | 0.90±0.10  (*p*=0.7338) | 0.89±0.13  (*p*=0.8335) | 0.85±0.06  (*p*=0.2155) | 0.90±0.07  (*p*=0.5666) | 1.03±0.06  (*p*=0.9804) | 1.05±0.01  (*p*=0.1164) | 0.91±0.07  (*p*=0.5849) | 0.99±0.02  (*p*=0.9449 | 1.04±0.06  (*p*=0.8992) | 1.04±0.15  (*p*=0.9993) |

| **DiNP** | **1 nM** | **50 nM** | **100 nM** | **250 nM** | **500 nM** | **1 µM** | **2.5 µM** | **5 µM** | **10 µM** | **25 µM** | **50 µM** | **100 µM** |
| --- | --- | --- | --- | --- | --- | --- | --- | --- | --- | --- | --- | --- |
| **progesterone** | 1.03±0.04  (*p*>0.9999) | 0.99±0.03  (*p*>0.9999) | 0.95±0.01  (*p*>0.9999) | 0.89±0.02  (*p*>0.9999) | 0.97±0.06  (*p*>0.9999) | 1.06±0.20  (*p*>0.9999) | 1.08±0.07  (*p*>0.9999) | 0.84±0.04  (*p*>0.9999) | 0.53±0.09  (*p*=0.7369) | 0.42±0.10  (*p*=0.4980) | 0.38±0.14  (*p*=0.4092) | 0.34±0.17  (*p*=0.3253) |
| **11-deoxycorticosterone** | 1.02±0.04  (*p*>0.9999) | 1.04±0.04  (*p*>0.9999) | 1.05±0.00  (*p*>0.9999) | 1.06±0.01  (*p*>0.9999) | 1.14±0.04  (*p*>0.9999) | 1.24±0.22  (*p*=0.9968) | 1.37±0.12  (*p*=0.9148) | 1.09±0.08  (*p*>0.9999) | 0.70±0.03  (*p*=0.9808) | 0.63±0.07  (*p*=0.9181) | 0.59±0.02  (*p*=0.8609) | 0.53±0.05  (*p*=0.7474) |
| **corticosterone** | 1.02±0.02  (*p*>0.9999) | 1.01±0.04  (p>0.9999) | 1.01±0.05  (*p*>0.9999) | 1.09±0.03  (*p*>0.9999) | 1.44±0.27  (*p*=0.8062) | **2.47±1.20**  **(*p*=0.0002)** | **5.64±0.96**  **(*p*<0.0001)** | **5.73±1.12**  **(*p*<0.0001)** | **2.93±2.02**  **(*p*<0.0001)** | **2.68±1.73**  **(*p*<0.0001)** | **2.35±1.30**  **(*p*=0.0009)** | 1.86±0.70  (*p*=0.0939) |
| **aldosterone** | 0.97±0.02  (*p*>0.9999) | 1.00±0.03  (*p*>0.9999) | 1.01±0.04  (*p*>0.9999) | 1.05±0.02  (*p*>0.9999) | 1.31±0.15  (*p*=0.9767) | **2.07±0.14**  **(*p*=0.0180)** | **4.27±0.66**  **(*p*<0.0001)** | **4.29±0.77**  **(*p*<0.0001)** | **2.30±0.55**  **(*p*=0.0017)** | **2.18±0.48**  **(*p*=0.0059)** | 1.76±0.13  (*p*=0.1860) | 1.52±0.19  (*p*=0.6254) |
| **17-OH-progesterone** | 1.04±0.04  (*p*>0.9999) | 1.02±0.06  (*p*>0.9999) | 0.98±0.03  (*p*>0.9999) | 0.94±0.03  (*p*>0.9999) | 0.97±0.04  (*p*>0.9999) | 1.02±0.17  (*p*>0.9999) | 1.09±0.08  (*p*>0.9999) | 0.93±0.06  (*p*>0.9999) | 0.59±0.05  (*p*=0.8553) | 0.50±0.08  (*p*=0.6746) | 0.43±0.12  (*p*=0.5186) | 0.40±0.15  (*p*=0.4487) |
| **11-deoxycortisol** | 1.01±0.03  (*p*>0.9999) | 1.03±0.05  (*p*>0.9999) | 1.02±0.02  (*p*>0.9999) | 1.00±0.03  (*p*>0.9999) | 1.07±0.04  (*p*>0.9999) | 1.12±0.02  (*p*>0.9999) | 1.12±0.04  (*p*>0.9999) | 1.11±0.04  (*p*>0.9999) | 0.96±0.03  (*p*>0.9999) | 0.94±0.02  (*p*>0.9999) | 0.93±0.03  (*p*>0.9999) | 0.95±0.04  (*p*>0.9999) |
| **21-deoxycortisol** | 1.04±0.01  (*p*>0.9999) | 1.00±0.08  (*p*>0.9999) | 1.00±0.06  (*p*>0.9999) | 1.08±0.04  (*p*>0.9999) | 1.44±0.17  (*p*=0.8094) | **2.72±0.99**  **(*p*<0.0001)** | **6.71±1.07**  **(*p*<0.0001)** | **6.93±1.32**  **(*p*<0.0001)** | **2.60±1.22**  **(*p*<0.0001)** | **2.03±0.85**  **(*p*=0.0239)** | 1.59±0.30  (*p*=0.4599) | 1.24±0.13  (*p*=0.9970) |
| **cortisol** | 1.00±0.04  (*p*>0.9999) | 0.98±0.06  (*p*>0.9999) | 0.96±0.04  (*p*>0.9999) | 0.99±0.01  (*p*>0.9999) | 1.17±0.03  (*p*=0.9999) | 1.59±0.22  (*p*=0.4679) | **2.58±0.23**  **(*p*<0.0001)** | **2.83±0.39**  **(*p*<0.0001)** | **1.95±0.65**  **(*p*=0.0491)** | 1.87±0.50  (*p*=0.0897) | 1.76±0.37  (*p*=0.1893) | 1.59±0.22  (*p*=0.4645) |
| **cortisone** | 1.00±0.08  (*p*>0.9999) | 0.99±0.04  (*p*>0.9999) | 0.92±0.04  (*p*>0.9999) | 0.83±0.02  (*p*=0.9999) | 0.89±0.04  (*p*>0.9999) | 0.91±0.06  (*p*>0.9999) | 0.66±0.02  (*p*=0.9470) | 0.70±0.06  (*p*=0.9786) | 0.93±0.07  (*p*>0.9999) | 0.96±0.11  (*p*>0.9999) | 0.92±0.08  (*p*>0.9999) | 0.93±0.09  (*p*>0.9999) |
| **DHEA** | 0.96±0.03  (*p*>0.9999) | 1.04±0.11  (*p*>0.9999) | 1.00±0.05  (*p*>0.9999) | 1.02±0.04  (*p*>0.9999) | 1.05±0.12  (*p*>0.9999) | 1.12±0.11  (*p*>0.9999) | 1.04±0.04  (*p*>0.9999) | 1.03±0.05  (*p*>0.9999) | 0.90±0.02  (*p*>0.9999) | 0.87±0.07  (*p*>0.9999) | 0.79±0.04  (*p*=0.9992) | 0.84±0.07  (*p*>0.9999) |
| **DHEAS** | 0.99±0.06  (*p*>0.9999) | 0.98±0.06  (*p*>0.9999) | 0.94±0.03  (*p*>0.9999) | 0.95±0.02  (*p*>0.9999) | 1.00±0.02  (*p*>0.9999) | 1.02±0.02  (*p*>0.9999) | 0.93±0.09  (*p*>0.9999) | 0.92±0.07  (*p*>0.9999) | 1.02±0.01  (*p*>0.9999) | 1.01±0.06  (*p*>0.9999) | 0.99±0.04  (*p*>0.9999) | 1.06±0.03  (*p*>0.9999) |
| **androstenedione** | 1.01±0.03  (*p*>0.9999) | 1.01±0.07  (*p*>0.9999) | 1.02±0.04  (*p*>0.9999) | 1.03±0.05  (*p*>0.9999) | 1.04±0.03  (*p*>0.9999) | 1.02±0.08  (*p*>0.9999) | 1.02±0.02  (*p*>0.9999) | 0.96±0.02  (*p*>0.9999) | 0.85±0.02  (*p*>0.9999) | 0.83±0.02  (*p*=0.9998) | 0.80±0.02  (*p*=0.9993) | 0.81±0.07  (*p*=0.9997) |
| **testosterone** | 1.00±0.05  (*p*>0.9999) | 1.01±0.08  (*p*>0.9999) | 0.98±0.04  (*p*>0.9999) | 0.99±0.05  (*p*>0.9999) | 1.02±0.03  (*p*>0.9999) | 0.99±0.04  (*p*>0.9999) | 0.92±0.03  (*p*>0.9999) | 0.88±0.04  (*p*>0.9999) | 0.82±0.03  (*p*=0.9998) | 0.80±0.02  (*p*=0.9995) | 0.79±0.04  (*p*=0.9989) | 0.84±0.04  (*p*>0.9999) |
| **DHT** | 1.00±0.08  (*p*>0.9999) | 0.98±0.06  (*p*>0.9999) | 0.96±0.05  (*p*>0.9999) | 0.95±0.08  (*p*>0.9999) | 0.95±0.05  (*p*>0.9999) | 0.92±0.01  (*p*>0.9999) | 0.79±0.03  (*p*=0.9990) | 0.75±0.03  (*p*=0.9950) | 0.68±0.06  (*p*=0.9706) | 0.64±0.10  (*p*=0.9308) | 0.61±0.12  (*p*=0.8821) | 0.67±0.12  (*p*=0.9596) |
| **estradiol** | 1.01±0.03  (*p*>0.9999) | 1.02±0.03  (*p*>0.9999) | 1.04±0.02  (*p*>0.9999) | 1.05±0.02  (*p*>0.9999) | 1.15±0.01  (*p*>0.9999) | 1.23±0.03  (*p*=0.9974) | 1.23±0.07  (*p*=0.9980) | 1.27±0.07  (*p*=0.9919) | 1.35±0.02  (*p*=0.9409) | 1.35±0.06  (*p*=0.9359) | 1.36±0.06  (*p*=0.9239) | 1.39±0.08  (*p*=0.8935) |

| **DEHA** | **1 nM** | **50 nM** | **100 nM** | **250 nM** | **500 nM** | **1 µM** | **2.5 µM** | **5 µM** | **10 µM** | **25 µM** | **50 µM** | **100 µM** |
| --- | --- | --- | --- | --- | --- | --- | --- | --- | --- | --- | --- | --- |
| **progesterone** | 0.94±0.04  (*p*=0.9996) | 0.91±0.03  (*p*=0.9815) | 0.89±0.06  (*p*=0.9547) | 0.88±0.06  (*p*=0.9144) | 0.91±0.05  (*p*=0.9799) | 0.90±0.04  (*p*=0.9686) | 0.87±0.02  (*p*=0.8406) | 0.86±0.02  (*p*=0.8024) | 0.95±0.02  (*p*>0.9999) | 1.03±0.06  (*p*>0.9999) | 1.08±0.08  (*p*=0.9924) | 1.08±0.07  (*p*=0.9967) |
| **11-deoxycorticosterone** | 1.00±0.02  (*p*>0.9999) | 0.96±0.04  (*p*>0.9999) | 0.96±0.04  (*p*>0.9999) | 0.98±0.06  (*p*>0.9999) | 1.02±0.05  (*p*>0.9999) | 1.02±0.06  (*p*>0.9999) | 0.99±0.01  (*p*>0.9999) | 0.99±0.03  (*p*>0.9999) | 1.09±0.04  (*p*=0.9890) | 1.20±0.06  (*p*=0.3554) | 1.29±0.10  (*p*=0.0559) | **1.32±0.10**  **(*p*=0.0251)** |
| **corticosterone** | 0.99±0.03  (*p*>0.9999) | 0.93±0.09  (*p*=0.9990) | 0.92±0.09  (*p*=0.9966) | 0.97±0.13  (*p*>0.9999) | 1.00±0.11  (*p*>0.9999) | 1.03±0.12  (*p*>0.9999) | 0.96±0.03  (*p*>0.9999) | 1.00±0.04  (*p*>0.9999) | 1.21±0.08  (*p*=0.3442) | **1.49±0.16**  **(*p*<0.0001)** | **1.69±0.27**  **(*p*<0.0001)** | **1.84±0.28**  **(*p*<0.0001)** |
| **aldosterone** | 0.96±0.05  (*p*>0.9999) | 0.87±0.16  (*p*=0.8532) | 0.86±0.16  (*p*=0.7905) | 0.89±0.24  (*p*=0.9439) | 0.92±0.17  (*p*=0.9941) | 0.95±0.16  (*p*>0.9999) | 0.88±0.15  (*p*=0.9214) | 0.93±0.10  (*p*=0.9984) | 1.05±0.07  (*p*>0.9999) | 1.29±0.20  (*p*=0.0686) | **1.49±0.38**  **(*p*<0.0001)** | **1.65±0.39**  **(*p*<0.0001)** |
| **17-OH-progesterone** | 0.99±0.03  (*p*>0.9999) | 0.98±0.06  (*p*>0.9999) | 0.95±0.06  (*p*>0.9999) | 0.94±0.06  (*p*=0.9998) | 0.98±0.07  (*p*>0.9999) | 0.98±0.05  (*p*>0.9999) | 0.98±0.01  (*p*>0.9999) | 0.97±0.03  (*p*>0.9999) | 1.06±0.04  (*p*=0.9998) | 1.07±0.07  (*p*=0.9984) | 1.09±0.10  (*p*=0.9878) | 1.07±0.09  (*p*=0.9976) |
| **11-deoxycortisol** | 0.99±0.04  (*p*>0.9999) | 0.94±0.08  (*p*=0.9998) | 0.93±0.08  (*p*=0.9982) | 0.94±0.09  (*p*=0.9995) | 0.97±0.08  (*p*>0.9999) | 0.98±0.07  (*p*>0.9999) | 1.01±0.04  (*p*>0.9999) | 1.00±0.02  (*p*>0.9999) | 1.01±0.03  (*p*>0.9999) | 1.04±0.09  (*p*>0.9999) | 1.07±0.13  (*p*=0.9989) | 1.09±0.12  (*p*=0.9814) |
| **21-deoxycortisol** | 1.00±0.04  (*p*>0.9999) | 0.90±0.14  (*p*=0.9665) | 0.87±0.15  (*p*=0.8610) | 0.96±0.23  (*p*>0.9999) | 0.98±0.17  (*p*>0.9999) | 1.04±0.20  (*p*>0.9999) | 1.01±0.07  (*p*>0.9999) | 0.98±0.03  (*p*>0.9999) | 1.16±0.08  (*p*=0.6356) | **1.42±0.25**  **(*p*=0.0010)** | **1.57±0.36**  **(*p*<0.0001)** | **1.64±0.39**  **(*p*<0.0001)** |
| **cortisol** | 0.95±0.04  (*p*>0.9999) | 0.89±0.14  (*p*=0.9407) | 0.86±0.13  (*p*=0.7605) | 0.88±0.16  (*p*=0.8823) | 0.91±0.13  (*p*=0.9905) | 0.93±0.12  (*p*=0.9987) | 0.95±0.07  (*p*=0.9999) | 0.96±0.04  (*p*>0.9999) | 0.99±0.06  (*p*>0.9999) | 1.11±0.15  (*p*=0.9409) | 1.20±0.23  (*p*=0.3731) | 1.32±0.22  **(*p*=0.0303)** |
| **cortisone** | 0.91±0.04  (*p*=0.9820) | 0.88±0.12  (*p*=0.9232) | 0.85±0.10  (*p*=0.7356) | 0.85±0.11  (*p*=0.7296) | 0.87±0.08  (*p*=0.8463) | 0.87±0.06  (*p*=0.8785) | 0.92±0.05  (*p*=0.9915) | 0.92±0.05  (*p*=0.9935) | 0.80±0.02  (*p*=0.4108) | 0.76±0.08  (*p*=0.2014) | 0.75±0.08  (*p*=0.1663) | 0.77±0.05  (*p*=0.2207) |
| **DHEA** | 1.14±0.02  (*p*=0.8005) | 1.14±0.06  (*p*=0.8097) | 1.14±0.03  (*p*=0.7988) | 1.17±0.07  (*p*=0.6078) | 1.21±0.04  (*p*=0.3489) | 1.19±0.07  (*p*=0.4649) | 1.10±0.05  (*p*=0.9733) | 1.06±0.02  (*p*=0.9996) | 1.29±0.06  (*p*=0.0625) | **1.34±0.03**  **(*p*=0.0157)** | **1.43±0.07**  **(*p*=0.0009)** | **1.54±0.08**  **(*p*<0.0001)** |
| **DHEAS** | 0.95±0.02  (*p*>0.9999) | 0.96±0.07  (*p*>0.9999) | 0.91±0.05  (*p*=0.9857) | 0.90±0.06  (*p*=0.9758) | 0.93±0.07  (*p*=0.9991) | 0.93±0.06  (*p*=0.9983) | 0.93±0.12  (*p*=0.9988) | 0.92±0.08  (*p*=0.9944) | 0.92±0.04  (*p*=0.9942) | 0.92±0.06  (*p*=0.9958) | 0.95±0.08  (*p*>0.9999) | 0.97±0.06  (*p*>0.9999) |
| **androstenedione** | 1.05±0.02  (*p*>0.9999) | 1.08±0.02  (*p*=0.9954) | 1.05±0.01  (*p*>0.9999) | 1.06±0.04  (*p*=0.9993) | 1.09±0.02  (*p*=0.9906) | 1.06±0.01  (*p*=0.9994) | 1.05±0.03  (*p*=0.9999) | 1.04±0.00  (*p*>0.9999) | 1.14±0.03  (*p*=0.8076) | 1.15±0.02  (*p*=0.7585) | 1.19±0.04  (*p*=0.4197) | 1.22±0.05  (*p*=0.2781) |
| **testosterone** | 0.97±0.03  (*p*>0.9999) | 0.96±0.12  (*p*>0.9999) | 0.91±0.08  (*p*=0.9895) | 0.91±0.08  (*p*=0.9803) | 0.95±0.10  (*p*=0.9999) | 0.96±0.07  (*p*>0.9999) | 0.99±0.04  (*p*>0.9999) | 0.96±0.03  (*p*>0.9999) | 0.96±0.04  (*p*>0.9999) | 0.94±0.09  (*p*=0.9996) | 0.94±0.13  (*p*=0.9998) | 0.93±0.11  (*p*=0.9982) |
| **DHT** | 0.93±0.04  (*p*=0.9991) | 0.95±0.11  (*p*>0.9999) | 0.91±0.06  (*p*=0.9903) | 0.89±0.04  (*p*=0.9460) | 0.92±0.06  (*p*=0.9959) | 0.92±0.02  (*p*=0.9966) | 0.89±0.02  (*p*=0.9292) | 0.90±0.02  (*p*=0.9578) | 0.95±0.01  (*p*>0.9999) | 0.96±0.06  (*p*>0.9999) | 1.00±0.09  (*p*>0.9999) | 1.02±0.07  (*p*>0.9999) |
| **estradiol** | 0.98±0.04  (*p*>0.9999) | 0.94±0.16  (*p*=0.9997) | 0.89±0.13  (*p*=0.9467) | 0.92±0.17  (*p*=0.9951) | 0.92±0.15  (*p*=0.9914) | 0.92±0.15  (*p*=0.9921) | 0.98±0.05  (*p*>0.9999) | 0.97±0.05  (*p*>0.9999) | 0.93±0.06  (*p*=0.9979) | 0.97±0.15  (*p*>0.9999) | 0.99±0.20  (*p*>0.9999) | 1.03±0.18  (*p*>0.9999) |

| **DEHT** | **1 nM** | **50 nM** | **100 nM** | **250 nM** | **500 nM** | **1 µM** | **2.5 µM** | **5 µM** | **10 µM** | **25 µM** | **50 µM** | **100 µM** |
| --- | --- | --- | --- | --- | --- | --- | --- | --- | --- | --- | --- | --- |
| **progesterone** | 1.00±0.04  (*p*>0.9999) | 1.00±0.01  (*p*>0.9999) | 0.96±0.04  (*p*>0.9999) | 0.97±0.02  (*p*>0.9999) | 1.00±0.04  (*p*>0.9999) | 1.10±0.14  (*p*>0.9999) | 1.22±0.13  (*p*>0.9999) | 1.15±0.10  (*p*>0.9999) | 1.15±0.21  (*p*>0.9999) | 1.13±0.23  (*p*>0.9999) | 0.97±0.14  (*p*>0.9999) | 0.70±0.09  (*p*=0.9983) |
| **11-deoxycorticosterone** | 1.02±0.04  (*p*>0.9999) | 1.05±0.01  (*p*>0.9999) | 1.03±0.08  (*p*>0.9999) | 1.08±0.06  (*p*>0.9999) | 1.1±0.05  (*p*>0.9999) | 1.37±0.27  (*p*=0.9903) | 2.11±0.16  (*p*=0.1208) | 2.02±0.08  (*p*=0.1939) | 1.75±0.33  (*p*=0.5421) | 1.80±0.36  (*p*=0.4555) | 1.94±0.15  (*p*=0.2747) | 1.71±0.02  (*p*=0.6071) |
| **corticosterone** | 0.96±0.05  (*p*>0.9999) | 1.01±0.02  (*p*>0.9999) | 0.97±0.09  (*p*>0.9999) | 1.00±0.11  (*p*>0.9999) | 1.04±0.04  (*p*>0.9999) | 1.75±0.88  (*p*=0.5485) | **3.16±0.30**  **(*p*<0.0001)** | **3.10±0.49**  **(*p*<0.0001)** | **3.00±2.25**  **(*p*=0.0002)** | **3.23±2.54**  **(*p*<0.0001)** | **4.05±3.00**  **(*p*<0.0001)** | **3.52±2.40**  **(*p*<0.0001)** |
| **aldosterone** | 0.98±0.06  (*p*>0.9999) | 0.90±0.14  (*p*>0.9999) | 0.90±0.03  (*p*>0.9999) | 0.92±0.05  (*p*>0.9999) | 0.99±0.06  (*p*>0.9999) | 1.29±0.16  (*p*=0.9988) | 2.11±0.16  (*p*=0.1213) | 2.26±0.61  (*p*=0.0558) | 1.78±0.52  (*p*=0.4979) | 2.01±0.73  (*p*=0.2034) | **2.67±0.70**  **(*p*=0.0033)** | **2.31±0.36**  **(*p*=0.0416)** |
| **17-OH-progesterone** | 1.03±0.03  (*p*>0.9999) | 1.03±0.05  (*p*>0.9999) | 0.97±0.03  (*p*>0.9999) | 0.98±0.01  (*p*>0.9999) | 1.00±0.01  (*p*>0.9999) | 1.04±0.08  (*p*>0.9999) | 0.95±0.03  (*p*>0.9999) | 0.99±0.02  (*p*>0.9999) | 0.93±0.09  (*p*>0.9999) | 0.90±0.11  (*p*>0.9999) | 0.71±0.06  (*p*=0.9987) | 0.64±0.09  (*p*=0.9909) |
| **11-deoxycortisol** | 1.00±0.02  (*p*>0.9999) | 1.03±0.03  (*p*>0.9999) | 0.98±0.02  (*p*>0.9999) | 1.01±0.02  (*p*>0.9999) | 1.01±0.01  (*p*>0.9999) | 1.10±0.07  (*p*>0.9999) | 1.27±0.05  (*p*=0.9994) | 1.30±0.07  (*p*=0.9984) | 1.19±0.04  (*p*>0.9999) | 1.21±0.03  (*p*>0.9999) | 1.27±0.04  (*p*=0.9994) | 1.25±0.06  (*p*=0.9998) |
| **21-deoxycortisol** | 1.01±0.05  (*p*>0.9999) | 0.99±0.05  (*p*>0.9999) | 0.91±0.04  (*p*>0.9999) | 0.91±0.05  (*p*>0.9999) | 1.03±0.10  (*p*>0.9999) | 1.58±0.68  (*p*=0.8219) | 2.18±0.17  (*p*=0.0836) | 2.18±0.21  (*p*=0.0867) | **2.29±1.48**  **(*p*=0.0451)** | **2.43±1.67**  **(*p*=0.0191)** | **2.72±1.76**  **(*p*=0.0023)** | 1.94±0.90  (*p*=0.2690) |
| **cortisol** | 1.01±0.02  (*p*>0.9999) | 0.98±0.04  (*p*>0.9999) | 0.92±0.03  (*p*>0.9999) | 0.94±0.01  (*p*>0.9999) | 1.01±0.01  (*p*>0.9999) | 1.22±0.17  (*p*>0.9999) | 1.53±0.07  (*p*=0.8825) | 1.57±0.15  (*p*=0.8317) | 1.48±0.37  (*p*=0.9357) | 1.57±0.45  (*p*=0.8342) | 1.84±0.55  (*p*=0.3976) | 1.79±0.56  (*p*=0.4694) |
| **cortisone** | 0.99±0.01  (*p*>0.9999) | 0.99±0.05  (*p*>0.9999) | 0.93±0.04  (*p*>0.9999) | 0.90±0.03  (*p*>0.9999) | 0.91±0.04  (*p*>0.9999) | 0.93±0.06  (*p*>0.9999) | 0.82±0.03  (*p*>0.9999) | 0.84±0.03  (*p*>0.9999) | 0.91±0.08  (*p*>0.9999) | 0.90±0.09  (*p*>0.9999) | 0.85±0.02  (*p*>0.9999) | 0.91±0.07  (*p*>0.9999) |
| **DHEA** | 0.96±0.04  (*p*>0.9999) | 0.98±0.02  (*p*>0.9999) | 0.93±0.03  (*p*>0.9999) | 0.94±0.04  (*p*>0.9999) | 0.93±0.05  (*p*>0.9999) | 1.07±0.10  (*p*>0.9999) | 1.34±0.07  (*p*=0.9944) | 1.36±0.04  (*p*=0.9917) | 1.3±0.08  (*p*=0.9984) | 1.35±0.13  (*p*=0.9935) | 1.57±0.15  (*p*=0.8340) | 1.74±0.39  (*p*=0.5522) |
| **DHEAS** | 0.96±0.02  (*p*>0.9999) | 0.94±0.03  (*p*>0.9999) | 0.88±0.04  (*p*>0.9999) | 0.90±0.00  (*p*>0.9999) | 0.95±0.05  (*p*>0.9999) | 1.02±0.07  (*p*>0.9999) | 1.15±0.08  (*p*>0.9999) | 1.15±0.02  (*p*>0.9999) | 1.11±0.00  (*p*>0.9999) | 1.15±0.03  (*p*>0.9999) | 1.27±0.03  (*p*=0.9993) | 1.38±0.19  (*p*=0.9875) |
| **androstenedione** | 1.00±0.03  (*p*>0.9999) | 1.02±0.04  (*p*>0.9999) | 1.01±0.03  (*p*>0.9999) | 1.08±0.01  (*p*>0.9999) | 1.08±0.04  (*p*>0.9999) | 1.20±0.08  (*p*>0.9999) | 1.40±0.09  (*p*=0.9816) | 1.39±0.05  (*p*=0.9834) | 1.46±0.05  (*p*=0.9525) | 1.49±0.05  (*p*=0.9263) | 1.67±0.05  (*p*=0.6787) | 1.76±0.15  (*p*=0.5208) |
| **testosterone** | 1.02±0.04  (*p*>0.9999) | 1.02±0.04  (*p*>0.9999) | 0.98±0.04  (*p*>0.9999) | 1.02±0.02  (*p*>0.9999) | 1.05±0.05  (*p*>0.9999) | 1.13±0.05  (*p*>0.9999) | 1.17±0.06  (*p*>0.9999) | 1.19±0.02  (*p*>0.9999) | 1.17±0.05  (*p*>0.9999) | 1.19±0.05  (*p*>0.9999) | 1.23±0.09  (*p*=0.9998) | 1.29±0.05  (*p*=0.9988) |
| **DHT** | 1.00±0.05  (*p*>0.9999) | 0.99±0.06  (*p*>0.9999) | 0.95±0.06  (*p*>0.9999) | 0.97±0.05  (*p*>0.9999) | 0.97±0.08  (*p*>0.9999) | 0.92±0.10  (*p*>0.9999) | 0.62±0.01  (*p*=0.9876) | 0.67±0.02  (*p*=0.9965) | 0.70±0.17  (*p*=0.9983) | 0.66±0.18  (*p*=0.9948) | 0.52±0.15  (*p*=0.9322) | 0.54±0.13  (*p*=0.9469) |
| **estradiol** | 0.99±0.05  (*p*>0.9999) | 1.08±0.01  (*p*>0.9999) | 1.00±0.04  (*p*>0.9999) | 1.07±0.08  (*p*>0.9999) | 1.07±0.06  (*p*>0.9999) | 1.18±0.07  (*p*>0.9999) | 1.28±0.04  (*p*=0.9992) | 1.28±0.06  (*p*=0.9990) | 1.23±0.06  (*p*=0.9998) | 1.25±0.07  (*p*=0.9997) | 1.36±0.04  (*p*=0.9925) | 1.39±0.13  (*p*=0.9842) |

| **DINCH** | **1 nM** | **50 nM** | **100 nM** | **250 nM** | **500 nM** | **1 µM** | **2.5 µM** | **5 µM** | **10 µM** | **25 µM** | **50 µM** | **100 µM** |
| --- | --- | --- | --- | --- | --- | --- | --- | --- | --- | --- | --- | --- |
| **progesterone** | 1.02±0.03  (*p*>0.9999) | 0.99±0.20  (*p*>0.9999) | 0.85±0.21  (*p*>0.9999) | 0.9±0.24  (*p*>0.9999) | 1.01±0.35  (*p*>0.9999) | 0.99±0.29  (*p*>0.9999) | 1.22±0.06  (*p*=0.9993) | 0.32±0.01  (*p*>0.9999) | 1.17±0.66  (*p*>0.9999) | 0.73±0.44  (*p*=0.9960) | 0.86±0.64  (*p*>0.9999) | 0.98±0.75  (*p*>0.9999) |
| **11-deoxycorticosterone** | 1.05±0.05  (*p*>0.9999) | 1.08±0.09  (*p*>0.9999) | 1.01±0.12  (*p*>0.9999) | 1.09±0.19  (*p*>0.9999) | 1.20±0.37  (*p*=0.9998) | 1.10±0.26  (*p*>0.9999) | 1.76±0.11  (*p*=0.2724) | 0.82±0.04  (*p*>0.9999) | 1.08±0.19  (*p*>0.9999) | 1.11±0.31  (*p*>0.9999) | 1.16±0.36  (*p*>0.9999) | 1.08±0.34  (*p*>0.9999) |
| **corticosterone** | 1.09±0.13  (*p*>0.9999) | 1.25±0.05  (*p*=0.9981) | 1.22±0.10  (*p*=0.9993) | 1.32±0.02  (*p*=0.9827) | **2.20±0.95**  **(*p*=0.0157)** | 2.01±0.73  (*p*=0.0594) | **5.63±0.39**  **(*p*<0.0001)** | **5.26±0.42**  **(*p*<0.0001)** | **2.93±1.51**  **(*p*<0.0001)** | **2.59±1.25**  **(*p*=0.0003)** | **3.02±1.51**  **(*p*<0.0001)** | **2.37±0.63**  **(*p*=0.0028)** |
| **aldosterone** | 0.87±0.06  (*p*>0.9999) | 1.07±0.13  (*p*=0.9468) | 1.1±0.13  (*p*=0.8697) | 1.23±0.15  (*p*=0.6255) | **1.97±0.49**  **(*p*=0.0034)** | **1.90±0.52**  **(*p*=0.0077)** | **4.39±0.87**  **(*p*<0.0001)** | **3.87±0.17**  **(*p*<0.0001)** | **2.76±1.08**  **(*p*<0.0001)** | **1.82±0.72**  **(*p*=0.0119)** | **2.32±0.80**  **(*p*<0.0001)** | **2.28±0.62**  **(*p*<0.0001)** |
| **17-OH-progesterone** | 1.07±0.02  (*p*>0.9999) | 0.99±0.18  (*p*>0.9999) | 0.88±0.26  (*p*>0.9999) | 0.91±0.28  (*p*>0.9999) | 0.97±0.36  (*p*>0.9999) | 0.94±0.30  (*p*>0.9999) | 1.18±0.05  (*p*>0.9999) | 0.49±0.01  (*p*>0.9999) | 0.92±0.34  (*p*>0.9999) | 0.56±0.25  (*p*=0.8631) | 0.62±0.32  (*p*=0.9440) | 0.67±0.39  (*p*=0.9808) |
| **11-deoxycortisol** | 1.07±0.05  (*p*>0.9999) | 1.07±0.09  (*p*>0.9999) | 1.05±0.06  (*p*>0.9999) | 1.07±0.10  (*p*>0.9999) | 1.05±0.12  (*p*>0.9999) | 1.04±0.15  (*p*>0.9999) | 1.28±0.08  (*p*=0.9947) | 1.06±0.04  (*p*>0.9999) | 0.99±0.03  (*p*>0.9999) | 1.02±0.05  (*p*>0.9999) | 1.05±0.04  (*p*>0.9999) | 1.03±0.00  (*p*>0.9999) |
| **21-deoxycortisol** | 1.10±0.21  (*p*>0.9999) | 1.39±0.01  (*p*=0.9367) | 1.27±0.14  (*p*=0.9955) | 1.45±0.10  (*p*=0.8613) | **3.04±1.31**  **(*p*<0.0001)** | **2.59±0.73**  **(*p*=0.0003)** | **5.86±0.38**  **(*p*<0.0001)** | **3.72±0.29**  **(*p*<0.0001)** | **3.55±1.28**  **(*p*<0.0001)** | 1.53±0.70  (*p*=0.7153) | 1.88±0.64  (*p*=0.1387) | 1.82±0.29  (*p*=0.2053) |
| **cortisol** | 1.12±0.13  (*p*>0.9999) | 1.25±0.12  (*p*=0.9980) | 1.26±0.22  (*p*=0.9972) | 1.30±0.16  (*p*=0.9902) | 1.72±0.21  (*p*=0.3360) | 1.64±0.15  (*p*=0.4818) | **2.32±0.10**  **(*p*=0.0044)** | **2.63±0.06**  **(*p*=0.0002)** | 2.00±0.84  (*p*=0.0654) | 1.72±0.74  (*p*=0.3417) | 1.93±0.74  (*p*=0.1041) | 1.68±0.33  (*p*=0.4027) |
| **cortisone** | 1.07±0.07  (*p*>0.9999) | 1.08±0.10  (*p*>0.9999) | 1.12±0.14  (*p*>0.9999) | 1.06±0.15  (*p*>0.9999) | 1.06±0.19  (*p*>0.9999) | 1.08±0.10  (*p*>0.9999) | 0.71±0.03  (*p*=0.9925) | 0.65±0.01  (*p*=0.9701) | 0.91±0.03  (*p*>0.9999) | 0.81±0.09  (*p*=0.9998) | 0.88±0.09  (*p*>0.9999) | 0.90±0.08  (*p*>0.9999) |
| **DHEA** | 1.16±0.16  (*p*>0.9999) | 1.23±0.40  (*p*=0.9990) | 1.15±0.38  (*p*>0.9999) | 1.12±0.40  (*p*>0.9999) | 1.23±0.39  (*p*=0.9991) | 1.08±0.33  (*p*>0.9999) | 1.11±0.11  (*p*>0.9999) | 1.11±0.08  (*p*>0.9999) | 1.57±0.46  (*p*=0.6163) | **2.07±1.06**  **(*p*=0.0392)** | **2.19±0.99**  **(*p*=0.0146)** | 1.79±0.81  (*p*=0.2376) |
| **DHEAS** | 1.13±0.12  (*p*>0.9999) | 1.23±0.20  (*p*=0.9989) | 1.25±0.27  (*p*=0.9978) | 1.18±0.25  (*p*=0.9999) | 1.22±0.25  (*p*=0.9993) | 1.18±0.13  (*p*>0.9999) | 1.01±0.02  (*p*>0.9999) | 1.05±0.01  (*p*>0.9999) | 1.03±0.07  (*p*>0.9999) | 1.17±0.15  (*p*>0.9999) | 1.19±0.17  (*p*=0.9999) | 1.21±0.15  (*p*=0.9997) |
| **androstenedione** | 1.14±0.12  (*p*>0.9999) | 1.08±0.22  (*p*>0.9999) | 0.99±0.21  (*p*>0.9999) | 1.00±0.23  (*p*>0.9999) | 0.97±0.24  (*p*>0.9999) | 0.93±0.19  (*p*>0.9999) | 1.11±0.04  (*p*>0.9999) | 0.99±0.04  (*p*>0.9999) | 1.02±0.08  (*p*>0.9999) | 1.14±0.19  (*p*>0.9999) | 1.12±0.16  (*p*>0.9999) | 1.02±0.11  (*p*>0.9999) |
| **testosterone** | 1.13±0.12  (*p*>0.9999) | 1.06±0.18  (*p*>0.9999) | 0.99±0.18  (*p*>0.9999) | 0.99±0.18  (*p*>0.9999) | 0.94±0.18  (*p*>0.9999) | 0.94±0.17  (*p*>0.9999) | 1.07±0.04  (*p*>0.9999) | 0.91±0.01  (*p*>0.9999) | 0.91±0.04  (*p*>0.9999) | 1.05±0.11  (*p*>0.9999) | 1.06±0.09  (*p*>0.9999) | 0.99±0.03  (*p*>0.9999) |
| **DHT** | 1.13±0.13  (*p*>0.9999) | 0.93±0.20  (*p*>0.9999) | 0.82±0.28  (*p*>0.9999) | 0.82±0.28  (*p*=0.9999) | 0.71±0.26  (*p*=0.9924) | 0.71±0.24  (*p*=0.9926) | 0.85±0.05  (*p*>0.9999) | 0.49±0.01  (*p*=0.7420) | 0.58±0.11  (*p*=0.8696) | 0.28±0.06  (*p*=0.3378) | 0.31±0.04  (*p*=0.3896) | 0.41±0.04  (*p*=0.5726) |
| **estradiol** | 1.11±0.09  (*p*>0.9999) | 1.22±0.19  (*p*=0.9994) | 1.26±0.24  (*p*=0.9970) | 1.24±0.18  (*p*=0.9985) | 1.34±0.16  (*p*=0.9717) | 1.31±0.04  (*p*=0.9858) | 1.36±0.04  (*p*=0.9610) | 1.48±0.07  (*p*=0.8019) | 1.26±0.14  (*p*=0.9965) | 1.32±0.13  (*p*=0.9819) | 1.41±0.03  (*p*=0.9143) | 1.45±0.13  (*p*=0.8548) |

| **Mixture (DEHP, DiBP,**  **DiNP, DEHA,**  **DEHT, DINCH)** | **1 nM** | **50 nM** | **100 nM** | **250 nM** | **500 nM** | **1 µM** | **2.5 µM** | **5 µM** | **10 µM** | **25 µM** | **50 µM** | **100 µM** |
| --- | --- | --- | --- | --- | --- | --- | --- | --- | --- | --- | --- | --- |
| **progesterone** | 0.90±0.07  (*p*>0.9999) | 0.91±0.06  (*p*>0.9999) | 0.89±0.08  (*p*>0.9999) | 0.92±0.07  (*p*>0.9999) | 0.86±0.07  (*p*>0.9999) | 0.54±0.09  (*p*=0.9899) | 1.10±0.04  (*p*>0.9999) | 1.2±0.10  (*p*>0.9999) | 0.82±0.10  (*p*>0.9999) | 0.69±0.21  (*p*=0.9997) | 0.63±0.29  (*p*=0.9984) | 1.14±0.08  (*p*>0.9999) |
| **11-deoxycorticosterone** | 0.94±0.06  (*p*>0.9999) | 0.94±0.09  (*p*>0.9999) | 0.98±0.09  (*p*>0.9999) | 1.06±0.14  (*p*>0.9999) | 1.08±0.16  (*p*>0.9999) | 1.06±0.12  (*p*>0.9999) | 1.78±0.07  (*p*=0.7546) | 1.66±0.04  (*p*=0.8894) | 1.07±0.02  (*p*>0.9999) | 1.08±0.06  (*p*>0.9999) | 1.04±0.15  (*p*>0.9999) | 1.20±0.22  (*p*>0.9999) |
| **corticosterone** | 0.99±0.02  (*p*>0.9999) | 0.95±0.04  (*p*>0.9999) | 0.95±0.05  (*p*>0.9999) | 1.50±0.30  (*p*=0.9803) | 1.92±0.64  (*p*=0.5637) | **2.68±1.31**  **(*p*=0.0331)** | **4.70±0.46**  **(*p*<0.0001)** | **6.46±0.28**  **(*p*<0.0001)** | **4.49±2.56**  **(*p*<0.0001)** | **4.15±2.21**  **(*p*<0.0001)** | **3.22±1.31**  **(*p*=0.0014)** | **5.79±3.09**  **(*p*<0.0001)** |
| **aldosterone** | 0.92±0.04  (*p*>0.9999) | 0.90±0.07  (*p*>0.9999) | 0.94±0.07  (*p*>0.9999) | 1.02±0.04  (*p*>0.9999) | 1.02±0.05  (*p*>0.9999) | 1.15±0.08  (*p*>0.9999) | **3.21±0.45**  **(*p*=0.0015)** | **5.03±0.52**  **(*p*<0.0001)** | 1.07±0.04  (*p*=0.0754) | 1.06±0.06  (*p*=0.9994) | 1.03±0.07  (*p*=0.8668) | **1.12±0.09**  **(*p*=0.0002)** |
| **17-OH-progesterone** | 0.90±0.08  (*p*>0.9999) | 0.88±0.10  (*p*>0.9999) | 0.88±0.12  (*p*>0.9999) | 0.91±0.01  (*p*>0.9999) | 0.85±0.06  (*p*>0.9999) | 0.81±0.03  (*p*>0.9999) | 1.10±0.02  (*p*>0.9999) | 1.08±0.04  (*p*>0.9999) | 0.78±0.17  (*p*>0.9999) | 0.64±0.24  (*p*=0.9989) | 0.55±0.30  (*p*=0.9924) | 0.92±0.14  (*p*>0.9999) |
| **11-deoxycortisol** | 0.92±0.04  (*p*>0.9999) | 0.90±0.07  (*p*>0.9999) | 0.94±0.07  (*p*>0.9999) | 1.02±0.04  (*p*>0.9999) | 1.02±0.05  (*p*>0.9999) | 1.15±0.08  (*p*>0.9999) | 1.21±0.03  (*p*>0.9999) | 1.13±0.02  (*p*>0.9999) | 1.07±0.04  (*p*>0.9999) | 1.06±0.06  (*p*>0.9999) | 1.03±0.07  (*p*>0.9999) | 1.12±0.09  (*p*>0.9999) |
| **21-deoxycortisol** | 0.90±0.09  (*p*>0.9999) | 0.86±0.09  (*p*>0.9999) | 0.85±0.16  (*p*>0.9999) | 1.24±0.22  (*p*>0.9999) | 1.56±0.40  (*p*=0.9609) | 1.73±0.52  (*p*=0.8215) | **4.17±0.36**  **(*p*<0.0001)** | **6.29±0.49**  **(*p*<0.0001)** | **4.54±2.74**  **(*p*<0.0001)** | **3.00±1.70**  **(*p*=0.0059)** | 2.10±0.64  (*p*=0.3502) | **6.37±3.97**  **(*p*<0.0001)** |
| **cortisol** | 0.92±0.03  (*p*>0.9999) | 0.89±0.06  (*p*>0.9999) | 0.88±0.09  (*p*>0.9999) | 1.08±0.20  (*p*>0.9999) | 1.24±0.28  (*p*>0.9999) | 1.33±0.23  (*p*=0.9996) | 1.98±0.11  (*p*=0.4923) | 2.50±0.07  (*p*=0.0785) | 2.15±0.87  (*p*=0.2992) | 1.94±0.71  (*p*=0.5426) | 1.73±0.35  (*p*=0.8146) | 2.45±0.75  (*p*=0.0970) |
| **cortisone** | 0.93±0.02  (*p*>0.9999) | 0.90±0.03  (*p*>0.9999) | 0.87±0.08  (*p*>0.9999) | 0.77±0.05  (*p*>0.9999) | 0.72±0.11  (*p*>0.9999) | 0.70±0.04  (*p*=0.9998) | 0.65±0.02  (*p*=0.9992) | 0.61±0.04  (*p*=0.9975) | 0.78±0.12  (*p*>0.9999) | 0.70±0.14  (*p*=0.9998) | 0.67±0.17  (*p*=0.9995) | 0.73±0.11  (*p*>0.9999) |
| **DHEA** | 1.06±0.02  (*p*>0.9999) | 1.01±0.03  (*p*>0.9999) | 1.02±0.07  (*p*>0.9999) | 1.06±0.10  (*p*>0.9999) | 1.08±0.10  (*p*>0.9999) | 1.14±0.26  (*p*>0.9999) | 1.17±0.04  (*p*>0.9999) | 1.14±0.07  (*p*>0.9999) | 1.10±0.11  (*p*>0.9999) | 1.11±0.09  (*p*>0.9999) | 0.95±0.09  (*p*>0.9999) | 1.20±0.14  (*p*>0.9999) |
| **DHEAS** | 0.91±0.03  (*p*>0.9999) | 0.89±0.03  (*p*>0.9999) | 0.89±0.03  (*p*>0.9999) | 0.87±0.03  (*p*>0.9999) | 0.90±0.08  (*p*>0.9999) | 0.95±0.20  (*p*>0.9999) | 1.01±0.05  (*p*>0.9999) | 0.98±0.02  (*p*>0.9999) | 0.91±0.01  (*p*>0.9999) | 0.87±0.05  (*p*>0.9999) | 0.86±0.08  (*p*>0.9999) | 0.87±0.04  (*p*>0.9999) |
| **androstenedione** | 0.95±0.07  (*p*>0.9999) | 0.93±0.09  (*p*>0.9999) | 0.96±0.09  (*p*>0.9999) | 1.02±0.03  (*p*>0.9999) | 1.03±0.03  (*p*>0.9999) | 1.13±0.12  (*p*>0.9999) | 1.16±0.05  (*p*>0.9999) | 1.09±0.04  (*p*>0.9999) | 1.01±0.06  (*p*>0.9999) | 1.00±0.10  (*p*>0.9999) | 0.96±0.10  (*p*>0.9999) | 1.01±0.06  (*p*>0.9999) |
| **testosterone** | 0.93±0.06  (*p*>0.9999) | 0.90±0.09  (*p*>0.9999) | 0.92±0.13  (*p*>0.9999) | 0.90±0.05  (*p*>0.9999) | 0.88±0.06  (*p*>0.9999) | 0.98±0.07  (*p*>0.9999) | 1.06±0.03  (*p*>0.9999) | 0.97±0.01  (*p*>0.9999) | 0.89±0.16  (*p*>0.9999) | 0.86±0.19  (*p*>0.9999) | 0.84±0.23  (*p*>0.9999) | 0.85±0.10  (*p*>0.9999) |
| **DHT** | 0.89±0.05  (*p*>0.9999) | 0.85±0.07  (*p*>0.9999) | 0.87±0.11  (*p*>0.9999) | 0.84±0.03  (*p*>0.9999) | 0.81±0.03  (*p*>0.9999) | 0.96±0.23  (*p*>0.9999) | 0.86±0.04  (*p*>0.9999) | 0.77±0.04  (*p*>0.9999) | 0.69±0.27  (*p*=0.9997) | 0.59±0.30  (*p*=0.9966) | 0.56±0.38  (*p*=0.9935) | 0.71±0.16  (*p*=0.9999) |
| **estradiol** | 0.96±0.06  (*p*>0.9999) | 0.99±0.07  (*p*>0.9999) | 1.00±0.05  (*p*>0.9999) | 1.06±0.04  (*p*>0.9999) | 1.07±0.05  (*p*>0.9999) | 1.23±0.08  (*p*>0.9999) | 1.24±0.12  (*p*=0.9996) | 1.37±0.10  (*p*=0.9912) | 1.25±0.20  (*p*>0.9999) | 1.29±0.22  (*p*=0.9998) | 1.23±0.21  (*p*>0.9999) | 1.24±0.16  (*p*>0.9999) |
