# Supplementary material for: Disruptive effects of phthalates and their substitutes on adrenal steroidogenesis
Source: Front Endocrinol (Lausanne). 2026 Jan 14;16:1734184. doi: 10.3389/fendo.2025.1734184 (PMC12848149; doi:10.3389/fendo.2025.1734184)
Supplement: Supplementary file 4 [file DataSheet4.docx]

**Supplementary Material S4.** Calculated substrate-product ratios after treatment, listed after the respective enzymatic step. Data are presented as fold-changes compared to vehicle-treated controls (n = 3; mean ± SD**).** Respective *p*-values are presented for each data point, while statistically significant values are marked in bold and grey background.

| **DEHP** | **calculated steroid ratio** | **1 nM** | **50 nM** | **100 nM** | **250 nM** | **500 nM** | **1 µM** | **2.5 µM** | **5 µM** | **10 µM** | **25 µM** | **50 µM** | **100 µM** |
| --- | --- | --- | --- | --- | --- | --- | --- | --- | --- | --- | --- | --- | --- |
| **CYP11B1** | 17-OH-progesterone/21-deoxycortisol | 0.96±0.10  (*p*>0.9999) | 1.07±0.18  (*p*=0.9985) | 0.88±0.00  (*p*=0.9023) | 0.80±0.10  (*p*=0.4028) | **0.66±0.14**  **(*p*=0.0123)** | **0.51±0.17**  **(*p*=0.0014)** | **0.16±0.02**  **(*p*<0.0001*)*** | **0.13±0.01**  **(*p*<0.0001*)*** | **0.43±0.18**  **(*p*=0.0001)** | **0.38±0.04**  **(*p*<0.0001*)*** | **0.40±0.01**  **(*p*<0.0001*)*** | **0.52±0.11**  **(*p*<0.0001*)*** |
| **CYP11B1** | 11-deoxycortisol/cortisol | 0.99±0.08  (*p*>0.9999) | 1.00±0.05  (*p*>0.9999) | 1.00±0.04  (*p*>0.9999) | 0.93±0.05  (*p*=0.9958) | 0.87±0.03  (*p*=0.7606) | 0.80±0.06  (*p*=0.2337) | **0.42±0.01**  **(*p*<0.0001)** | **0.36±0.02**  **(*p*<0.0001)** | **0.71±0.13**  **(*p*=0.0308)** | **0.53±0.03**  **(*p*=0.0002)** | **0.58±0.03**  **(*p*=0.0008)** | 0.86±0.14  (*p*=0.6949) |
| **CYP11B1** | 11-deoxycorticosterone/  corticosterone | 0.91±0.09  (*p*=0.9865) | 0.97±0.05  (*p*>0.9999) | 0.84±0.09  (*p*=0.7062) | 0.75±0.16  (*p*=0.2040) | **0.62±0.18**  **(*p*=0.0220)** | **0.53±0.17**  **(*p*=0.0005)** | **0.25±0.03**  **(*p*<0.0001*)*** | **0.22±0.02**  **(*p*<0.0001*)*** | **0.43±0.14**  **(*p*<0.0001*)*** | **0.36±0.07**  **(*p*<0.0001*)*** | **0.37±0.09**  **(*p*<0.0001*)*** | **0.40±0.09**  **(*p*=0.0006)** |
| **CYP11B2** | corticosterone/  aldosterone | 1.04±0.07  (*p*>0.9999) | 1.06±0.05  (*p*>0.9999) | 1.19±0.12  (*p*=0.9877) | 1.21±0.17  (*p*=0.9707) | 1.49±0.37  (*p*=0.2390) | 1.52±0.21  (*p*=0.1837) | 1.29±0.09  (*p*=0.8054) | 1.14±0.10  (*p*=0.9992) | 1.52±0.09  (*p*=0.1958) | 1.56±0.37  (*p*=0.1387) | **1.68±0.54**  **(*p*=0.0420)** | 1.14±0.06  (*p*=0.9988) |
| **CYP17A1** | progesterone/  17-OH-progesterone | 0.91±0.03  (*p*=0.9990) | 0.96±0.05  (*p*>0.9999) | 0.95±0.01  (*p*>0.9999) | 0.98±0.03  (*p*>0.9999) | 1.09±0.09  (*p*=0.9991) | 1.12±0.12  (*p*=0.9846) | 1.27±0.01  (*p*=0.4022) | 1.35±0.02  (*p*=0.1492) | 1.35±0.10  (*p*=0.1335) | 1.23±0.07  (*p*=0.5636) | 1.20±0.10  (*p*=0.7186) | 1.15±0.27  (*p*=0.9444) |
| **CYP17A1** | 17-OH-progesterone/  androstenedione | 0.99±0.07  (*p*>0.9999) | 1.02±0.06  (*p*>0.9999) | 0.99±0.01  (*p*>0.9999) | 1.04±0.13  (*p*>0.9999) | 1.02±0.17  (*p*>0.9999) | 1.02±0.23  (*p*>0.9999) | 1.12±0.06  (*p*=0.9751) | 1.11±0.05  (*p*=0.9941) | 0.93±0.27  (*p*=0.9997) | 0.62±0.08  (*p*=0.0554) | **0.49±0.09**  **(*p*=0.0050)** | **0.49±0.15**  **(*p*=0.0047)** |
| **CYP19A1** | testosterone/estradiol | 1.01±0.04  (*p*>0.9999) | 1.03±0.04  (*p*>0.9999) | 0.99±0.01  (*p*>0.9999) | 0.98±0.03  (*p*>0.9999) | 0.90±0.03  (*p*=0.9991) | 0.86±0.04  (*p*=0.9899) | 0.73±0.01  (*p*=0.5725) | 0.65±0.01  (*p*=0.2644) | 0.58±0.05  (*p*=0.1286) | 0.93±0.44  (*p*>0.9999) | 0.91±0.42  (*p*=0.9999) | **0.46±0.06**  **(*p*=0.0239)** |
| **CYP21A2** | progesterone/  11-deoxycorticosterone | 0.97±0.13  (*p*>0.9999) | 1.01±0.11  (*p*>0.9999) | 0.95±0.06  (*p*>0.9999) | 0.99±0.11  (*p*>0.9999) | 0.95±0.13  (*p*>0.9999) | 0.91±0.15  (*p*=0.9950) | 0.91±0.03  (*p*=0.9976) | 1.03±0.02  (*p*>0.9999) | 1.15±0.31  (*p*=0.8715) | 0.93±0.02  (*p*=0.9995) | 0.73±0.08  (*p*=0.2594) | 0.72±0.13  (*p*=0.2224) |
| **CYP21A2** | 17-OH-progesterone/11-deoxycortisol | 1.00±0.07  (*p*>0.9999) | 1.03±0.08  (*p*>0.9999) | 0.99±0.06  (*p*>0.9999) | 1.05±0.08  (*p*=0.9997) | 0.98±0.10  (*p*>0.9999) | 0.92±0.15  (*p*=0.9846) | 0.93±0.05  (*p*=0.9936) | 0.95±0.06  (*p*=0.9999) | 0.82±0.18  (*p*=0.3169) | **0.56±0.06**  **(*p*=0.0003)** | **0.43±0.07**  **(*p*<0.0001*)*** | **0.40±0.10**  **(*p*<0.0001*)*** |
| **CYP21A2** | 21-deoxycortisol/cortisol | 1.03±0.14  (*p*>0.9999) | 0.98±0.14  (*p*>0.9999) | 1.12±0.03  (*p*=0.9983) | 1.23±0.10  (*p*=0.7962) | 1.34±0.19  (*p*=0.3509) | **1.55±0.30**  **(*p*=0.0272)** | **2.50±0.23**  **(*p*<0.0001*)*** | **2.68±0.22**  **(*p*<0.0001*)*** | 1.50±0.28  (*p*=0.0584) | 0.80±0.10  (*p*=0.8818) | 0.63±0.08  (*p*=0.2599) | 0.67±0.13  (*p*=0.3695) |
| **HSD3B2** | DHEA/androstenedione | 1.03±0.10  (*p*>0.9999) | 1.07±0.16  (*p*=0.9957) | 1.17±0.14  (*p*=0.4977) | 1.06±0.08  (*p*=0.9984) | 1.05±0.05  (*p*=0.9997) | 1.03±0.03  (*p*>0.9999) | 0.95±0.03  (*p*=0.9997) | 0.99±0.06  (*p*>0.9999) | 1.09±0.10  (*p*=0.9710) | 1.02±0.11  (*p*>0.9999) | 1.00±0.12  (*p*>0.9999) | 0.75±0.09  (*p*=0.1106) |
| **HSD11B1/2** | cortisol/cortisone | 0.99±0.04  (*p*>0.9999) | 1.00±0.05  (*p*>0.9999) | 1.08±0.05  (*p*>0.9999) | 1.27±0.21  (*p*=0.9943) | 1.64±0.54  (*p*=0.4417) | 1.89±0.67  (*p*=0.1255) | **4.05±0.29**  **(*p*<0.0001*)*** | **4.48±0.29**  **(*p*<0.0001*)*** | **2.10±0.66**  **(*p*=0.0372)** | 1.83±0.19  (*p*=0.1821) | 1.66±0.06  (*p*=0.4072) | 1.53±0.34  (*p*=0.6738) |
| **HSD17B3** | androstenedione/testosterone | 1.01±0.03  (*p*>0.9999) | 0.98±0.02  (*p*>0.9999) | 1.00±0.02  (*p*>0.9999) | 1.03±0.02  (*p*>0.9999) | 1.05±0.08  (*p*=0.9988) | 1.05±0.09  (*p*=0.9996) | 1.03±0.01  (*p*>0.9999) | 1.06±0.02  (*p*=0.9933) | 1.13±0.10  (*p*=0.5656) | 1.07±0.01  (*p*=0.9886) | 1.07±0.03  (*p*=0.9850) | 1.07±0.14  (*p*=0.9784) |
| **SULT2A1** | DHEA/DHEAS | 1.02±0.12  (*p*>0.9999) | 1.08±0.16  (*p*=0.9970) | 1.22±0.11  (*p*=0.4274) | 1.16±0.10  (*p*=0.7716) | 1.23±0.21  (*p*=0.3897) | 1.20±0.22  (*p*=0.5626) | 0.92±0.08  (*p*=0.9969) | 0.94±0.05  (*p*=0.9998) | 1.14±0.07  (*p*=0.8806) | 0.75±0.02  (*p*=0.2626) | 0.73±0.01  (*p*=0.2043) | 0.74±0.03  (*p*=0.2393) |
| **5α-reductase** | testosterone/DHT | 1.04±0.04  (*p*>0.9999) | 1.02±0.02  (*p*>0.9999) | 1.03±0.01  (*p*>0.9999) | 1.00±0.08  (*p*>0.9999) | 1.02±0.09  (*p*>0.9999) | 1.04±0.09  (*p*>0.9999) | 1.17±0.01  (*p*>0.9999) | 1.23±0.04  (*p*=0.9998) | 1.26±0.30  (*p*=0.9994) | 1.66±0.19  (*p*=0.6656) | 1.98±0.21  (*p*=0.2245) | **2.35±0.86**  **(*p*=0.0363)** |

| **DiBP** | **calculated steroid ratio** | **1 nM** | **50 nM** | **100 nM** | **250 nM** | **500 nM** | **1 µM** | **2.5 µM** | **5 µM** | **10 µM** | **25 µM** | **50 µM** | **100 µM** |
| --- | --- | --- | --- | --- | --- | --- | --- | --- | --- | --- | --- | --- | --- |
| **CYP11B1** | 17-OH-progesterone/21-deoxycortisol | 1.12±0.11  (*p*=0.9947) | 1.06±0.03  (*p*>0.9999) | 1.04±0.04  (*p*>0.9999) | 1.08±0.08  (*p*=0.9998) | 1.24±0.29  (*p*=0.6708) | 1.24±0.37  (*p*=0.6991) | 0.96±0.06  (*p*>0.9999) | 0.95±0.04  (*p*>0.9999) | 1.11±0.29  (*p*=0.9968) | 0.78±0.05  (*p*=0.7644) | 0.76±0.03  (*p*=0.6870) | 0.76±0.12  (*p*=0.6643) |
| **CYP11B1** | 11-deoxycortisol/cortisol | 1.07±0.05  (*p*=0.9929) | 1.07±0.05  (*p*=0.9960) | 1.07±0.05  (*p*=0.9958) | 1.07±0.03  (*p*=0.9939) | 1.09±0.11  (*p*=0.9732) | 1.06±0.15  (*p*=0.9986) | 0.92±0.03  (*p*=0.9778) | 0.93±0.07  (*p*=0.9961) | 1.01±0.13  (*p*>0.9999) | 0.88±0.10  (*p*=0.8027) | 0.88±0.11  (*p*=0.8109) | 0.92±0.18  (*p*=0.9890) |
| **CYP11B1** | 11-deoxycorticosterone/corticosterone | 1.05±0.05  (*p*=0.9987) | 1.08±0.07  (*p*=0.9662) | 1.08±0.10  (*p*=0.9504) | 1.08±0.08  (*p*=0.9318) | 1.08±0.10  (*p*=0.9503) | 1.06±0.13  (*p*=0.9889) | 1.04±0.04  (*p*>0.9999) | 0.97±0.03  (*p*>0.9999) | 0.99±0.12  (*p*>0.9999) | 0.80±0.09  (*p*=0.1532) | 0.77±0.06  (*p*=0.0765) | 0.80±0.10  (*p*=0.1731) |
| **CYP11B2** | corticosterone/aldosterone | 1.04±0.02  (*p*=0.9977) | 1.01±0.05  (*p*>0.9999) | 1.02±0.03  (*p*>0.9999) | 1.04±0.05  (*p*=0.9948) | 1.07±0.05  (*p*=0.9169) | 1.09±0.09  (*p*=0.7009) | **1.22±0.07**  **(*p*=0.0104)** | **1.22±0.07**  **(*p*=0.0133)** | 1.06±0.09  (*p*=0.9557) | 0.98±0.04  (*p*>0.9999) | 1.01±0.09  (*p*>0.9999) | 1.05±0.06  (*p*=0.9760) |
| **CYP17A1** | progesterone/17-OH-progesterone | 0.96±0.05  (*p*=0.9924) | 0.94±0.06  (*p*=0.9302) | 0.95±0.05  (*p*=0.9461) | 0.94±0.05  (*p*=0.9174) | 0.93±0.01  (*p*=0.7728) | 0.96±0.02  (*p*=0.9979) | 0.99±0.01  (*p*>0.9999) | 0.99±0.01  (*p*>0.9999) | 0.98±0.07  (*p*>0.9999) | 1.03±0.05  (*p*=0.9991) | 1.08±0.06  (*p*=0.7212) | 1.08±0.13  (*p*=0.7236) |
| **CYP17A1** | 17-OH-progesterone/androstenedione | 1.07±0.07  (*p*=0.9973) | 1.00±0.09  (*p*>0.9999) | 0.98±0.09  (*p*>0.9999) | 1.00±0.08  (*p*>0.9999) | 1.08±0.09  (*p*=0.9909) | 1.08±0.08  (*p*=0.9869) | 1.01±0.05  (*p*>0.9999) | 0.96±0.04  (*p*>0.9999) | 1.00±0.07  (*p*>0.9999) | 0.98±0.08  (*p*>0.9999) | 0.91±0.14  (*p*=0.9780) | **0.70±0.24**  **(*p*=0.0496)** |
| **CYP19A1** | testosterone/estradiol | 1.10±0.06  (*p*=0.9874) | 0.99±0.07  (*p*>0.9999) | 1.01±0.07  (*p*>0.9999) | 1.06±0.03  (*p*=0.9999) | 1.18±0.17  (*p*=0.7457) | 1.14±0.24  (*p*=0.9287) | 0.99±0.03  (*p*>0.9999) | 0.98±0.02  (*p*>0.9999) | 1.11±0.16  (*p*=0.9764) | 0.99±0.03  (*p*>0.9999) | 0.97±0.09  (*p*>0.9999) | 1.18±0.30  (*p*=0.7553) |
| **CYP21A2** | progesterone/11-deoxycorticosterone | 1.06±0.08  (*p*=0.9971) | 0.99±0.03  (*p*>0.9999) | 1.01±0.01  (*p*>0.9999) | 1.01±0.05  (*p*>0.9999) | 1.10±0.12  (*p*=0.8628) | 1.12±0.16  (*p*=0.7299) | 0.94±0.02  (*p*=0.9941) | 0.91±0.05  (*p*=0.9524) | 1.07±0.13  (*p*=0.9889) | 1.01±0.07  (*p*>0.9999) | 1.05±0.10  (*p*=0.9993) | 0.93±0.14  (*p*=0.9902) |
| **CYP21A2** | 17-OH-progesterone/11-deoxycortisol | 1.14±0.08  (*p*=0.9654) | 1.12±0.06  (*p*=0.9885) | 1.11±0.08  (*p*=0.9923) | 1.13±0.06  (*p*=0.9754) | 1.30±0.15  (*p*=0.3168) | 1.31±0.19  (*p*=0.2984) | 1.13±0.08  (*p*=0.9730) | 1.08±0.11  (*p*=0.9996) | 1.24±0.16  (*p*=0.5800) | 1.06±0.10  (*p*>0.9999) | 1.04±0.21  (*p*>0.9999) | 0.91±0.33  (*p*=0.9984) |
| **CYP21A2** | 21-deoxycortisol/cortisol | 1.10±0.09  (*p*=0.9646) | 1.12±0.08  (*p*=0.8757) | 1.14±0.09  (*p*=0.7511) | 1.12±0.04  (*p*=0.8668) | 1.15±0.03  (*p*=0.6772) | 1.14±0.02  (*p*=0.7436) | 1.07±0.04  (*p*=0.9935) | 1.05±0.03  (*p*=0.9998) | 1.14±0.02  (*p*=0.7306) | 1.19±0.04  (*p*=0.4469) | 1.18±0.14  (*p*=0.5032) | 1.07±0.30  (*p*=0.9949) |
| **HSD3B2** | DHEA/androstenedione | 1.04±0.06  (*p*>0.9999) | 1.15±0.06  (*p*=0.7404) | 1.13±0.04  (*p*=0.8651) | 1.10±0.05  (*p*=0.9658) | 1.17±0.07  (*p*=0.6505) | 1.08±0.05  (*p*=0.9956) | 1.02±0.05  (*p*>0.9999) | 1.04±0.02  (*p*>0.9999) | 1.12±0.06  (*p*=0.9316) | 1.10±0.04  (*p*=0.9747) | 1.10±0.08  (*p*=0.9658) | 0.97±0.36  (*p*>0.9999) |
| **HSD11B1/2** | cortisol/cortisone | 0.97±0.01  (*p*>0.9999) | 0.95±0.07  (*p*=0.9992) | 0.93±0.09  (*p*=0.9934) | 0.95±0.05  (*p*=0.9998) | 0.98±0.01  (*p*>0.9999) | 1.00±0.03  (*p*>0.9999) | 1.06±0.02  (*p*=0.9972) | 1.19±0.05  (*p*=0.3404) | 1.08±0.04  (*p*=0.9861) | 1.25±0.13  (*p*=0.1001) | 1.25±0.13  (*p*=0.1079) | **1.30±0.26**  **(*p*=0.0388)** |
| **HSD17B3** | androstenedione/testosterone | 1.04±0.01  (*p*=0.9998) | 1.12±0.09  (*p*=0.6324) | 1.12±0.13  (*p*=0.5929) | 1.12±0.13  (*p*=0.6006) | 1.14±0.04  (*p*=0.4606) | 1.13±0.04  (*p*=0.5298) | 1.02±0.02  (*p*>0.9999) | 1.03±0.01  (*p*>0.9999) | 1.14±0.08  (*p*=0.4596) | 1.06±0.00  (*p*=0.9906) | 1.05±0.07  (*p*=0.9984) | 1.03±0.15  (*p*>0.9999) |
| **SULT2A1** | DHEA/DHEAS | 1.14±0.08  (*p*=0.9616) | 1.30±0.13  (*p*=0.3515) | 1.30±0.16  (*p*=0.3559) | 1.30±0.15  (*p*=0.3559) | 1.46±0.02  (*p*=0.3488) | 1.35±0.09  (*p*=0.0506) | 1.09±0.11  (*p*=0.9992) | 1.13±0.16  (*p*=0.9858) | 1.39±0.11  (*p*=0.1328) | 1.19±0.05  (*p*=0.8186) | 1.18±0.16  (*p*=0.8589) | 1.09±0.39  (*p*=0.9987) |
| **5α-reductase** | testosterone/DHT | 0.93±0.08  (*p*>0.9999) | 0.98±0.08  (*p*>0.9999) | 0.98±0.07  (*p*>0.9999) | 0.98±0.08  (*p*>0.9999) | 0.88±0.14  (*p*=0.9817) | 0.87±0.17  (*p*=0.9785) | 1.12±0.02  (*p*=0.9869) | 1.13±0.02  (*p*=0.9720) | 0.91±0.18  (*p*=0.9980) | 1.00±0.01  (*p*>0.9999) | 1.11±0.12  (*p*=0.9942) | **1.45±0.40**  **(*p*=0.0426)** |
|  | | | | | | | | | | | | | |
| **DiNP** | **calculated steroid ratio** | **1 nM** | **50 nM** | **100 nM** | **250 nM** | **500 nM** | **1 µM** | **2.5 µM** | **5 µM** | **10 µM** | **25 µM** | **50 µM** | **100 µM** |
| **CYP11B1** | 17-OH-progesterone/21-deoxycortisol | 1.00±0.05  (*p*>0.9999) | 1.02±0.03  (*p*>0.9999) | 0.98±0.03  (*p*>0.9999) | 0.87±0.05  (*p*=0.4795) | **0.68±0.05**  **(*p*=0.0022)** | **0.4±0.07**  **(*p*<0.0001)** | **0.16±0.02**  **(*p*<0.0001)** | **0.14±0.02**  **(*p*<0.0001)** | **0.28±0.11**  **(*p*<0.0001)** | **0.30±0.12**  **(*p*<0.0001)** | **0.29±0.11**  **(*p*<0.0001)** | **0.33±0.13**  **(*p*<0.0001)** |
| **CYP11B1** | 11-deoxycortisol/cortisol | 1.02±0.04  (*p*>0.9999) | 1.05±0.06  (*p*=0.9962) | 1.06±0.02  (*p*=0.9703) | 1.01±0.04  (*p*>0.9999) | 0.91±0.06  (*p*=0.8401) | **0.72±0.08**  **(*p*=0.0009)** | **0.44±0.02**  **(*p*<0.0001)** | **0.40±0.04**  **(*p*<0.0001)** | **0.54±0.14**  **(*p*<0.0001)** | **0.54±0.12**  **(*p*<0.0001)** | **0.55±0.09**  **(*p*<0.0001)** | **0.60±0.07**  **(*p*<0.0001)** |
| **CYP11B1** | 11-deoxycorticosterone/corticosterone | 1.00±0.05  (*p*>0.9999) | 1.03±0.04  (*p*>0.9999) | 1.05±0.05  (*p*=0.9996) | 0.98±0.02  (*p*>0.9999) | 0.82±0.11  (*p*=0.3469) | **0.57±0.14**  **(*p*=0.0048)** | **0.25±0.02**  **(*p*<0.0001)** | **0.2±0.03**  **(*p*<0.0001)** | **0.35±0.15**  **(*p*<0.0001)** | **0.32±0.13**  **(*p*<0.0001)** | **0.32±0.13**  **(*p*<0.0001)** | **0.33±0.12**  **(*p*<0.0001)** |
| **CYP11B2** | corticosterone/aldosterone | 1.05±0.02  (*p*>0.9999) | 1.01±0.04  (*p*>0.9999) | 1.00±0.05  (*p*>0.9999) | 1.03±0.01  (*p*>0.9999) | 1.14±0.34  (*p*>0.9999) | 1.23±0.68  (*p*=0.9991) | 1.32±0.04  (*p*=0.9887) | 1.33±0.07  (*p*=0.9844) | 1.15±0.52  (*p*>0.9999) | 1.13±0.48  (*p*>0.9999) | 1.38±0.84  (*p*=0.9599) | 1.28±0.58  (*p*=0.9963) |
| **CYP17A1** | progesterone/17-OH-progesterone | 1.00±0.01  (*p*>0.9999) | 0.98±0.05  (*p*>0.9999) | 0.97±0.02  (*p*>0.9999) | 0.94±0.01  (*p*=0.9926) | 1.00±0.02  (*p*>0.9999) | 1.04±0.02  (*p*=0.9997) | 1.00±0.01  (*p*>0.9999) | 0.9±0.02  (*p*=0.7535) | 0.89±0.09  (*p*=0.6978) | 0.83±0.07  (*p*=0.2196) | 0.85±0.12  (*p*=0.3015) | **0.76±0.20**  **(*p*=0.0328)** |
| **CYP17A1** | 17-OH-progesterone/androstenedione | 1.03±0.02  (*p*>0.9999) | 1.01±0.03  (*p*>0.9999) | 0.96±0.02  (*p*=0.9997) | 0.92±0.05  (*p*=0.9001) | 0.94±0.04  (*p*=0.9850) | 1.00±0.09  (*p*>0.9999) | 1.07±0.07  (*p*=0.9770) | 0.97±0.04  (*p*>0.9999) | **0.70±0.08**  **(*p*=0.0050)** | **0.61±0.08**  **(*p*=0.0002)** | **0.54±0.14**  **(*p*<0.0001)** | **0.48±0.16**  **(*p*<0.0001)** |
| **CYP19A1** | testosterone/estradiol | 0.99±0.07  (*p*>0.9999) | 0.99±0.07  (*p*>0.9999) | 0.94±0.04  (*p*=0.7210) | 0.94±0.05  (*p*=0.7880) | 0.89±0.03  (*p*=0.1105) | **0.81±0.05**  **(*p*=0.0016)** | **0.75±0.04**  **(*p*<0.0001)** | **0.70±0.01**  **(*p*<0.0001)** | **0.61±0.02**  **(*p*<0.0001)** | **0.59±0.04**  **(*p*<0.0001)** | **0.58±0.04**  **(*p*<0.0001)** | **0.60±0.05**  **(*p*<0.0001)** |
| **CYP21A2** | progesterone/11-deoxycorticosterone | 1.01±0.05  (*p*>0.9999) | 0.96±0.02  (*p*>0.9999) | 0.91±0.01  (*p*=0.9913) | 0.84±0.01  (*p*=0.7931) | 0.85±0.03  (*p*=0.8358) | 0.85±0.02  (*p*=0.8719) | 0.79±0.04  (*p*=0.5398) | 0.77±0.03  (*p*=0.4212) | 0.76±0.16  (*p*=0.3843) | 0.68±0.18  (*p*=0.1309) | 0.64±0.24  (*p*=0.0679) | **0.60±0.29**  **(*p*=0.0322)** |
| **CYP21A2** | 17-OH-progesterone/11-deoxycortisol | 1.03±0.02  (*p*>0.9999) | 0.99±0.02  (*p*>0.9999) | 0.96±0.02  (*p*>0.9999) | 0.94±0.04  (*p*=0.9957) | 0.91±0.07  (*p*=0.9233) | 0.90±0.14  (*p*=0.8724) | 0.97±0.06  (*p*>0.9999) | 0.84±0.04  (*p*=0.3732) | **0.61±0.07**  **(*p*=0.0010)** | **0.53±0.08**  **(*p*<0.0001)** | **0.47±0.14**  **(*p*<0.0001)** | **0.43±0.17**  **(*p*<0.0001)** |
| **CYP21A2** | 21-deoxycortisol/cortisol | 1.05±0.05  (*p*>0.9999) | 1.02±0.07  (*p*>0.9999) | 1.04±0.04  (*p*>0.9999) | 1.09±0.03  (*p*=0.9993) | 1.22±0.11  (*p*=0.7581) | **1.66±0.35**  **(*p*=0.0044)** | **2.60±0.28**  **(*p*<0.0001)** | **2.43±0.24**  **(*p*<0.0001)** | 1.28±0.17  (*p*=0.5082) | 1.05±0.18  (*p*>0.9999) | 0.91±0.07  (*p*=0.9996) | 0.78±0.09  (*p*=0.7892) |
| **HSD3B2** | DHEA/androstenedione | 0.95±0.00  (*p*=0.9449) | 1.02±0.05  (*p*>0.9999) | 0.98±0.08  (*p*>0.9999) | 0.99±0.08  (*p*>0.9999) | 1.01±0.10  (*p*>0.9999) | 1.10±0.07  (*p*=0.3307) | 1.02±0.02  (*p*>0.9999) | 1.07±0.03  (*p*=0.7180) | 1.06±0.03  (*p*=0.8309) | 1.05±0.06  (*p*=0.9630) | 0.99±0.03  (*p*>0.9999) | 1.03±0.03  (*p*=0.9996) |
| **HSD11B1/2** | cortisol/cortisone | 0.99±0.05  (*p*>0.9999) | 1.00±0.03  (*p*>0.9999) | 1.04±0.01  (*p*>0.9999) | 1.19±0.02  (*p*>0.9999) | 1.31±0.09  (*p*=0.9927) | 1.77±0.37  (*p*=0.4408) | **3.92±0.30**  **(*p*<0.0001)** | **4.04±0.26**  **(*p*<0.0001)** | 2.15±0.88  (*p*=0.0886) | 2.05±0.82  (*p*=0.1450) | 1.97±0.62  (*p*=0.2047) | 1.76±0.44  (*p*=0.4573) |
| **HSD17B3** | androstenedione/testosterone | 1.01±0.03  (*p*>0.9999) | 1.00±0.01  (*p*>0.9999) | 1.04±0.01  (*p*=0.9130) | 1.04±0.00  (*p*=0.8794) | 1.02±0.03  (*p*=0.9998) | 1.02±0.04  (*p*=0.9993) | 1.11±0.02  (*p*=0.0785) | 1.09±0.04  (*p*=0.2077) | 1.03±0.02  (*p*=0.9912) | 1.03±0.01  (*p*=0.9933) | 1.02±0.06  (*p*>0.9999) | 0.98±0.10  (*p*=0.9979) |
| **SULT2A1** | DHEA/DHEAS | 0.97±0.04  (*p*>0.9999) | 1.06±0.04  (*p*=0.9960) | 1.06±0.04  (*p*=0.9921) | 1.08±0.06  (*p*=0.9641) | 1.05±0.14  (*p*=0.9992) | 1.10±0.11  (*p*=0.8175) | 1.13±0.16  (*p*=0.6228) | 1.13±0.11  (*p*=0.6214) | 0.89±0.01  (*p*=0.7405) | 0.86±0.09  (*p*=0.5325) | 0.80±0.02  (*p*=0.1652) | 0.79±0.04  (*p*=0.1235) |
| **5α-reductase** | testosterone/DHT | 1.00±0.02  (*p*>0.9999) | 1.03±0.01  (*p*>0.9999) | 1.02±0.01  (*p*>0.9999) | 1.04±0.04  (*p*>0.9999) | 1.07±0.02  (*p*=0.9997) | 1.08±0.04  (*p*=0.9991) | 1.17±0.02  (*p*=0.8681) | 1.18±0.02  (*p*=0.8239) | 1.21±0.14  (*p*=0.6462) | 1.28±0.20  (*p*=0.3357) | 1.37±0.35  (*p*=0.1158) | 1.30±0.28  (*p*=0.2869) |

| **DEHA** | **calculated steroid ratio** | **1 nM** | **50 nM** | **100 nM** | **250 nM** | **500 nM** | **1 µM** | **2.5 µM** | **5 µM** | **10 µM** | **25 µM** | **50 µM** | **100 µM** |
| --- | --- | --- | --- | --- | --- | --- | --- | --- | --- | --- | --- | --- | --- |
| **CYP11B1** | 17-OH-progesterone/21-deoxycortisol | 0.99±0.04  (*p*>0.9999) | 1.11±0.12  (*p*=0.9300) | 1.11±0.14  (*p*=0.9103) | 1.03±0.19  (*p*>0.9999) | 1.02±0.11  (*p*>0.9999) | 0.96±0.13  (*p*>0.9999) | 0.98±0.07  (*p*>0.9999) | 0.99±0.03  (*p*>0.9999) | 0.91±0.03  (*p*=0.9785) | 0.77±0.10  (*p*=0.2263) | 0.72±0.11  (*p*=0.0893) | **0.68±0.10**  **(*p*=0.0373**) |
| **CYP11B1** | 11-deoxycortisol/cortisol | 1.04±0.02  (*p*=0.9949) | 1.07±0.08  (*p*=0.8376) | 1.1±0.09  (*p*=0.5462) | 1.09±0.10  (*p*=0.6546) | 1.07±0.07  (*p*=0.8912) | 1.06±0.07  (*p*=0.9215) | 1.07±0.03  (*p*=0.8540) | 1.05±0.03  (*p*=0.9906) | 1.03±0.03  (*p*=0.9999) | 0.95±0.06  (*p*=0.9699) | 0.90±0.07  (*p*=0.5666) | 0.84±0.06  (*p*=0.0980) |
| **CYP11B1** | 11-deoxycorticosterone/corticosterone | 1.01±0.01  (*p*>0.9999 | 1.03±0.06  (*p*=0.9992) | 1.04±0.06  (*p*=0.9807) | 1.02±0.08  (*p*=0.9999) | 1.02±0.06  (*p*=0.9999) | 0.99±0.06  (*p*>0.9999) | 1.04±0.03  (*p*=0.9929) | 0.99±0.02  (*p*>0.9999) | 0.90±0.02  (*p*=0.3952) | **0.81±0.05**  **(*p*=0.0112)** | **0.78±0.07**  **(*p*=0.0018)** | **0.73±0.06**  **(*p*=0.0002)** |
| **CYP11B2** | corticosterone/aldosterone | 1.03±0.05  (*p*>0.9999 | 1.09±0.09  (*p*=0.9567) | 1.09±0.10  (*p*=0.9440) | 1.13±0.15  (*p*=0.7750) | 1.11±0.11  (*p*=0.8975) | 1.10±0.07  (*p*=0.9385) | 1.11±0.14  (*p*=0.8879) | 1.08±0.06  (*p*=0.9794) | 1.15±0.02  (*p*=0.5790) | 1.17±0.07  (*p*=0.4515) | 1.17±0.13  (*p*=0.4357) | 1.14±0.11  (*p*=0.6845) |
| **CYP17A1** | progesterone/17-OH-progesterone | 0.95±0.03  (*p*=0.5305) | 0.93±0.02  (*p*=0.1248) | 0.94±0.02  (*p*=0.3351) | 0.94±0.04  (*p*=0.2208) | 0.93±0.03  (*p*=0.1244) | 0.92±0.03  (*p*=0.0683) | **0.88±0.02**  **(*p*=0.0036)** | **0.89±0.03**  **(*p*=0.0070)** | **0.90±0.03**  **(*p*=0.0146)** | 0.96±0.04  (*p*=0.7854) | 1.00±0.03  (*p*>0.9999) | 1.01±0.04  (*p*>0.9999) |
| **CYP17A1** | 17-OH-progesterone/androstenedione | 0.94±0.03  (*p*=0.7964) | 0.91±0.04  (*p*=0.2745) | 0.90±0.06  (*p*=0.2227) | 0.89±0.08  (*p*=0.1292) | 0.90±0.05  (*p*=0.2082) | 0.92±0.05  (*p*=0.4598) | 0.93±0.02  (*p*=0.6355) | 0.93±0.02  (*p*=0.6236) | 0.93±0.02  (*p*=0.5613) | 0.93±0.05  (*p*=0.6420) | 0.91±0.06  (*p*=0.3074) | 0.88±0.05  (*p*=0.0803) |
| **CYP19A1** | testosterone/estradiol | 0.99±0.07  (*p*>0.9999) | 1.03±0.06  (*p*=0.9997) | 1.03±0.06  (*p*=0.9996) | 1.00±0.10  (*p*>0.9999) | 1.05±0.07  (*p*=0.9947) | 1.06±0.10  (*p*=0.9558) | 1.01±0.02  (*p*>0.9999) | 0.99±0.02  (*p*>0.9999) | 1.03±0.03  (*p*=0.9996) | 0.98±0.06  (*p*>0.9999) | 0.97±0.09  (*p*>0.9999) | 0.91±0.06  (*p*=0.7079) |
| **CYP21A2** | progesterone/11-deoxycorticosterone | 0.94±0.02  (*p*>0.9999) | 0.95±0.01  (*p*>0.9999) | 0.93±0.02  (*p*>0.9999) | 0.90±0.01  (*p*>0.9999) | 0.89±0.02  (*p*>0.9999) | 0.88±0.02  (*p*>0.9999) | 0.87±0.01  (*p*=0.8459) | 0.87±0.01  (*p*=0.7599) | 0.87±0.02  (*p*=0.5730) | 0.85±0.02  (*p*=0.5699) | 0.84±0.01  (*p*=0.8239) | 0.81±0.02  (*p*=0.8569) |
| **CYP21A2** | 17-OH-progesterone/11-deoxycortisol | 0.99±0.02  (*p*>0.9999) | 1.04±0.03  (*p*=0.7606) | 1.02±0.03  (*p*=0.9907) | 1.01±0.05  (*p*>0.9999) | 1.01±0.03  (*p*>0.9999) | 1.00±0.01  (*p*>0.9999) | 0.97±0.04  (*p*=0.9531) | 0.97±0.04  (*p*=0.9065) | 1.04±0.02  (*p*=0.6558) | 1.03±0.02  (*p*=0.9467) | 1.02±0.04  (*p*=0.9851) | 0.98±0.03  (*p*=0.9994) |
| **CYP21A2** | 21-deoxycortisol/cortisol | 1.04±0.01  (*p*=0.9677) | 1.01±0.04  (*p*>0.9999) | 1.02±0.05  (*p*>0.9999) | 1.08±0.06  (*p*=0.4942) | 1.06±0.05  (*p*=0.7923) | 1.11±0.07  (*p*=0.2122) | 1.06±0.01  (*p*=0.7861) | 1.02±0.03  (*p*=0.9997) | **1.18±0.02**  **(*p*=0.0096)** | **1.28±0.06**  **(*p*=0.0024)** | **1.30±0.05**  **(*p*<0.0001)** | **1.23±0.09**  **(*p*=0.0005)** |
| **HSD3B2** | DHEA/androstenedione | 1.09±0.01  (*p*=0.1303) | 1.05±0.04  (*p*=0.6286) | 1.08±0.03  (*p*=0.1756) | 1.10±0.08  (*p*=0.0769) | **1.11±0.02**  **(*p*=0.0358)** | **1.12±0.05**  **(*p*=0.0243)** | 1.04±0.02  (*p*=0.8533) | 1.02±0.02  (*p*=0.9980) | **1.13±0.02**  **(*p*=0.0079)** | **1.17±0.03**  **(*p*=0.0005)** | **1.19±0.03**  **(*p*<0.0001)** | **1.26±0.03**  **(*p*<0.0001)** |
| **HSD11B1/2** | cortisol/cortisone | 1.05±0.01  (*p*=0.9986) | 1.00±0.05  (*p*>0.9999) | 1.00±0.07  (*p*>0.9999) | 1.02±0.06  (*p*>0.9999 | 1.05±0.07  (*p*=0.9992) | 1.06±0.07  (*p*=0.9942) | 1.03±0.03  (*p*>0.9999 | 1.04±0.01  (*p*=0.9997) | 1.22±0.06  (*p*=0.1015) | **1.45±0.07**  **(*p*=0.0002)** | **1.58±0.17**  **(*p*<0.0001)** | **1.70±0.18**  **(*p*<0.0001)** |
| **HSD17B3** | androstenedione/testosterone | 1.08±0.04  (*p*=0.9976) | 1.13±0.12  (*p*=0.7287) | 1.16±0.12  (*p*=0.5225) | 1.19±0.14  (*p*=0.3613) | 1.16±0.12  (*p*=0.5503) | 1.11±0.08  (*p*=0.8538) | 1.06±0.02  (*p*=0.9964) | 1.08±0.04  (*p*=0.9813) | 1.19±0.03  (*p*=0.3491) | 1.23±0.11  (*p*=0.1660) | 1.28±0.14  (*p*=0.0520) | **1.33±0.12**  **(*p*=0.0198)** |
| **SULT2A1** | DHEA/DHEAS | 1.20±0.04  (*p*=0.0555) | 1.19±0.03  (*p*=0.1796) | **1.25±0.05**  **(*p*=0.0098)** | **1.29±0.01**  **(*p*=0.0026)** | **1.29±0.05**  **(*p*=0.0024)** | **1.28±0.02**  **(*p*=0.0044)** | 1.2±0.16  (*p*=0.0639) | 1.16±0.11  (*p*=0.1812) | **1.40±0.01**  **(*p*<0.0001)** | **1.46±0.10**  **(*p*<0.0001)** | **1.50±0.07**  **(*p*<0.0001)** | **1.58±0.03**  **(*p*<0.0001)** |
| **5α-reductase** | testosterone/DHT | 1.04±0.02  (*p*=0.9742) | 1.01±0.04  (*p*>0.9999) | 1.00±0.04  (*p*>0.9999) | 1.01±0.05  (*p*>0.9999) | 1.02±0.04  (*p*=0.9987) | 1.04±0.05  (*p*=0.9733) | 1.12±0.03  (*p*=0.0569) | 1.07±0.01  (*p*=0.4196) | 1.01±0.04  (*p*>0.9999) | 0.98±0.04  (*p*=0.9998) | 0.94±0.05  (*p*=0.5702) | 0.91±0.05  (*p*=0.1706) |

| **DEHT** | **calculated steroid ratio** | **1 nM** | **50 nM** | **100 nM** | **250 nM** | **500 nM** | **1 µM** | **2.5 µM** | **5 µM** | **10 µM** | **25 µM** | **50 µM** | **100 µM** |
| --- | --- | --- | --- | --- | --- | --- | --- | --- | --- | --- | --- | --- | --- |
| **CYP11B1** | 17-OH-progesterone/21-deoxycortisol | 1.03±0.04  (*p*>0.9999) | 1.04±0.04  (*p*>0.9999) | 1.07±0.04  (*p*=0.9997) | 1.09±0.06  (*p*=0.9959) | 0.98±0.10  (*p*>0.9999) | 0.75±0.21  (*p*=0.3410) | **0.44±0.02**  **(*p*=0.0013)** | **0.46±0.04**  **(*p*=0.0020)** | **0.55±0.22**  **(*p*=0.0122)** | **0.52±0.22**  **(*p*=0.0069)** | **0.36±0.15**  **(*p*=0.0003)** | **0.39±0.14**  **(*p*=0.0005)** |
| **CYP11B1** | 11-deoxycortisol/cortisol | 0.98±0.01  (*p*>0.9999) | 1.06±0.01  (*p*=0.9994) | 1.07±0.02  (*p*=0.9957) | 1.08±0.00  (*p*=0.9887) | 1.00±0.01  (*p*>0.9999) | 0.91±0.07  (*p*=0.9754) | 0.83±0.02  (*p*=0.5171) | 0.83±0.03  (*p*=0.5233) | 0.84±0.16  (*p*=0.6192) | 0.82±0.18  (*p*=0.4920) | 0.75±0.19  (*p*=0.1357) | 0.75±0.18  (*p*=0.1433) |
| **CYP11B1** | 11-deoxycorticosterone/corticosterone | 1.07±0.10  (*p*>0.9999) | 1.04±0.01  (*p*>0.9999) | 1.06±0.01  (*p*>0.9999) | 1.08±0.05  (*p*>0.9999) | 1.05±0.02  (*p*>0.9999) | 0.89±0.22  (*p*=0.9996) | 0.67±0.01  (*p*=0.5545) | 0.66±0.07  (*p*=0.5239) | 0.84±0.34  (*p*=0.9851) | 0.83±0.35  (*p*=0.9832) | 0.72±0.33  (*p*=0.7246) | 0.71±0.33  (*p*=0.6893) |
| **CYP11B2** | corticosterone/aldosterone | 0.97±0.02  (*p*>0.9999) | 1.15±0.21  (*p*>0.9999) | 1.08±0.09  (*p*>0.9999) | 1.09±0.10  (*p*>0.9999) | 1.06±0.10  (*p*>0.9999) | 1.29±0.48  (*p*=0.9959) | 1.51±0.25  (*p*=0.8525) | 1.42±0.16  (*p*=0.9506) | 1.49±0.70  (*p*=0.8797) | 1.39±0.61  (*p*=0.9660) | 1.35±0.65  (*p*=0.9849) | 1.45±0.84  (*p*=0.9199) |
| **CYP17A1** | progesterone/17-OH-progesterone | 0.97±0.06  (*p*>0.9999) | 0.98±0.06  (*p*>0.9999) | 0.99±0.05  (*p*>0.9999) | 0.98±0.03  (*p*>0.9999) | 0.99±0.05  (*p*>0.9999) | 1.05±0.07  (*p*=0.9997) | 1.29±0.15  (*p*=0.0724) | 1.17±0.09  (*p*=0.5589) | 1.23±0.12  (*p*=0.2097) | 1.26±0.15  (*p*=0.1333) | **1.36±0.11**  **(*p*=0.0166)** | 1.12±0.21  (*p*=0.8623) |
| **CYP17A1** | 17-OH-progesterone/androstenedione | 1.03±0.02  (*p*=0.9850) | 1.01±0.01  (*p*>0.9999) | 0.96±0.01  (*p*=0.8969 | 0.91±0.01  (*p*=0.1422) | 0.93±0.02  (*p*=0.3427) | **0.86±0.04**  **(*p*=0.0068)** | **0.68±0.02**  **(*p*<0.0001)** | **0.71±0.03**  **(*p*<0.0001)** | **0.64±0.05**  **(*p*<0.0001)** | **0.60±0.07**  **(*p*<0.0001)** | **0.43±0.03**  **(*p*<0.0001)** | **0.36±0.06**  **(*p*<0.0001)** |
| **CYP19A1** | testosterone/estradiol | 1.04±0.08  (*p*=0.9986) | 0.94±0.03  (*p*=0.9767) | 0.98±0.08  (*p*>0.9999) | 0.96±0.09  (*p*=0.9988) | 0.98±0.09  (*p*>0.9999) | 0.96±0.05  (*p*=0.9986) | 0.92±0.02  (*p*=0.8537) | 0.93±0.04  (*p*=0.9066) | 0.95±0.08  (*p*=0.9964) | 0.96±0.09  (*p*=0.9974) | 0.91±0.09  (*p*=0.8062) | 0.93±0.05  (*p*=0.9412) |
| **CYP21A2** | progesterone/11-deoxycorticosterone | 0.98±0.01  (*p*>0.9999) | 0.96±0.02  (*p*=0.8870) | 0.94±0.04  (*p*=0.5682) | 0.90±0.05  (*p*=0.0921) | 0.91±0.03  (*p*=0.1620) | **0.81±0.07**  **(*p*=0.0004)** | **0.58±0.04**  **(*p*<0.0001)** | **0.57±0.04**  **(*p*<0.0001)** | **0.66±0.01**  **(*p*<0.0001)** | **0.63±0.04**  **(*p*<0.0001)** | **0.50±0.03**  **(*p*<0.0001)** | **0.41±0.06**  **(*p*<0.0001)** |
| **CYP21A2** | 17-OH-progesterone/11-deoxycortisol | 1.03±0.01  (*p*=0.9736) | 1.00±0.03  (*p*>0.9999) | 0.99±0.02  (*p*>0.9999) | 0.97±0.01  (*p*=0.9932) | 0.99±0.01  (*p*>0.9999) | 0.94±0.02  (*p*=0.6399) | **0.75±0.01**  **(*p*<0.0001)** | **0.76±0.03**  **(*p*<0.0001)** | **0.78±0.06**  **(*p*<0.0001)** | **0.74±0.08**  **(*p*<0.0001)** | **0.56±0.05**  **(*p*<0.0001)** | **0.51±0.06**  **(*p*<0.0001)** |
| **CYP21A2** | 21-deoxycortisol/cortisol | 0.99±0.04  (*p*>0.9999) | 1.01±0.03  (*p*>0.9999) | 0.99±0.03  (*p*>0.9999) | 0.97±0.05  (*p*>0.9999) | 1.02±0.11  (*p*>0.9999) | 1.24±0.35  (*p*=0.9740) | 1.42±0.05  (*p*=0.6327) | 1.39±0.01  (*p*=0.7309) | 1.41±0.55  (*p*=0.6624) | 1.39±0.55  (*p*=0.7246) | 1.34±0.46  (*p*=0.8452) | 1.04±0.16  (*p*>0.9999) |
| **HSD3B2** | DHEA/androstenedione | 0.95±0.01  (*p*=0.9701) | 0.96±0.03  (*p*=0.9967) | 0.92±0.00  (*p*=0.6651) | 0.87±0.03  (*p*=0.1673) | 0.86±0.03  (*p*=0.0968) | 0.89±0.02  (*p*=0.2772) | 0.96±0.02  (*p*=0.9932) | 0.98±0.02  (*p*>0.9999) | 0.89±0.04  (*p*=0.3030) | 0.90±0.07  (*p*=0.4544) | 0.94±0.08  (*p*=0.8928) | 0.98±0.14  (*p*>0.9999) |
| **HSD11B1/2** | cortisol/cortisone | 1.03±0.01  (*p*>0.9999) | 0.99±0.02  (*p*>0.9999) | 0.99±0.01  (*p*>0.9999) | 1.04±0.03  (*p*>0.9999) | 1.11±0.04  (*p*>0.9999) | 1.32±0.26  (*p*=0.9666) | 1.88±0.04  (*p*=0.1330) | 1.87±0.17  (*p*=0.1409) | 1.67±0.59  (*p*=0.3762) | 1.80±0.68  (*p*=0.2016) | **2.18±0.70**  **(*p*=0.0201)** | 1.95±0.44  (*p*=0.0892) |
| **HSD17B3** | androstenedione/testosterone | 0.98±0.02  (*p*>0.9999) | 1.00±0.01  (*p*>0.9999) | 1.03±0.02  (*p*=0.9997) | 1.06±0.01  (*p*=0.9048) | 1.03±0.01  (*p*=0.9983) | 1.06±0.04  (*p*=0.8253) | **1.19±0.01**  **(*p*=0.0077)** | **1.17±0.04**  **(*p*=0.0171)** | **1.24±0.09**  **(*p*=0.0005)** | **1.25±0.09**  **(*p*=0.0003)** | **1.36±0.10**  **(*p*<0.0001)** | **1.37±0.06**  **(*p*<0.0001)** |
| **SULT2A1** | DHEA/DHEAS | 1.00±0.03  (*p*>0.9999) | 1.05±0.01  (*p*=0.9804) | 1.05±0.01  (*p*=0.9644) | 1.04±0.04  (*p*=0.9906) | 0.98±0.00  (*p*>0.9999) | 1.05±0.05  (*p*=0.9837) | **1.17±0.03**  **(*p*=0.0363)** | **1.18±0.04**  **(*p*=0.0241)** | **1.17±0.07**  **(*p*=0.0474)** | **1.18±0.09**  **(*p*=0.0330)** | **1.23±0.09**  **(*p*=0.0027)** | **1.25±0.10**  **(*p*=0.0012)** |
| **5α-reductase** | testosterone/DHT | 1.03±0.01  (*p*>0.9999) | 1.03±0.03  (*p*>0.9999) | 1.03±0.02  (*p*>0.9999) | 1.05±0.03  (*p*>0.9999) | 1.08±0.04  (*p*>0.9999) | 1.24±0.14  (*p*=0.9962) | 1.89±0.09  (*p*=0.1440) | 1.76±0.06  (*p*=0.2741) | 1.79±0.47  (*p*=0.2330) | 1.94±0.52  (*p*=0.1058) | **2.60±0.74**  **(*p*=0.0014)** | **2.59±0.78**  **(*p*=0.0015)** |

| **DINCH** | **calculated steroid ratio** | **1 nM** | **50 nM** | **100 nM** | **250 nM** | **500 nM** | **1 µM** | **2.5 µM** | **5 µM** | **10 µM** | **25 µM** | **50 µM** | **100 µM** |
| --- | --- | --- | --- | --- | --- | --- | --- | --- | --- | --- | --- | --- | --- |
| **CYP11B1** | 17-OH-progesterone/21-deoxycortisol | 1.01±0.19  (*p*>0.9999) | 0.71±0.14  (*p*=0.5564) | 0.72±0.26  (*p*=0.5709) | 0.63±0.19  (*p*=0.2805) | **0.33±0.07**  **(*p*=0.0063)** | **0.36±0.04**  **(*p*=0.0097)** | **0.20±0.01**  **(*p*=0.0010)** | **0.13±0.01**  **(*p*=0.0004)** | **0.32±0.18**  **(*p*=0.0059)** | 0.52±0.33  (*p*=0.0766) | **0.40±0.24**  **(*p*=0.0184)** | **0.36±0.18**  **(*p*=0.0097)** |
| **CYP11B1** | 11-deoxycortisol/cortisol | 0.97±0.08  (*p*>0.9999) | 0.87±0.10  (*p*=0.9488) | 0.87±0.19  (*p*=0.9550) | 0.84±0.16  (*p*=0.8576) | 0.62±0.06  (*p*=0.0647) | 0.63±0.04  (*p*=0.0883) | **0.55±0.01**  **(*p*=0.0216)** | **0.40±0.01**  **(*p*=0.0015)** | **0.57±0.18**  **(*p*=0.0323)** | 0.70±0.26  (*p*=0.1114) | 0.62±0.21  (*p*=0.0530) | 0.64±0.11  (*p*=0.0525) |
| **CYP11B1** | 11-deoxycorticosterone/corticosterone | 0.98±0.03  (*p*>0.9999) | 0.91±0.11  (*p*=0.9893) | 0.83±0.12  (*p*=0.9496) | 0.82±0.11  (*p*=0.9220) | 0.84±0.15  (*p*=0.1127) | 0.88±0.12  (*p*=0.1120) | **0.31±0.01**  **(*p*=0.0029)** | **0.16±0.01**  **(*p*=0.0003)** | **0.52±0.21**  **(*p*=0.0399)** | 0.58±0.29  (*p*=0.2321) | 0.63±0.37  (*p*=0.0750) | 0.75±0.49  (*p*=0.0908) |
| **CYP11B2** | corticosterone/aldosterone | 1.02±0.17  (*p*>0.9999) | 0.98±0.25  (*p*>0.9999) | 0.93±0.25  (*p*>0.9999) | 0.90±0.22  (*p*>0.9999) | 0.94±0.41  (*p*>0.9999) | 0.91±0.39  (*p*>0.9999) | 1.33±0.23  (*p*=0.8559) | 1.36±0.08  (*p*=0.7796) | 0.88±0.30  (*p*>0.9999) | 1.16±0.33  (*p*=0.9990) | 1.06±0.36  (*p*>0.9999) | 0.88±0.23  (*p*>0.9999) |
| **CYP17A1** | progesterone/17-OH-progesterone | 0.95±0.05  (*p*>0.9999) | 1.01±0.14  (*p*>0.9999) | 0.98±0.09  (*p*>0.9999) | 1.01±0.07  (*p*>0.9999) | 1.06±0.03  (*p*>0.9999) | 1.06±0.06  (*p*>0.9999) | 1.03±0.04  (*p*>0.9999) | 0.66±0.02  (*p*=0.7937) | 1.14±0.37  (*p*=0.9995) | 1.16±0.45  (*p*=0.9987) | 1.14±0.48  (*p*=0.9995) | 1.16±0.51  (*p*=0.9987) |
| **CYP17A1** | 17-OH-progesterone/androstenedione | 0.95±0.08  (*p*>0.9999) | 0.92±0.12  (*p*>0.9999) | 0.88±0.11  (*p*=0.9933) | 0.89±0.10  (*p*=0.9980) | 0.96±0.15  (*p*>0.9999) | 0.99±0.14  (*p*>0.9999) | 1.06±0.03  (*p*>0.9999) | 0.49±0.01  (*p*=0.0509) | 0.90±0.33  (*p*=0.9987) | **0.46±0.16**  **(*p*=0.0336)** | 0.52±0.23  (*p*=0.0736) | 0.63±0.33  (*p*=0.2397) |
| **CYP19A1** | testosterone/estradiol | 1.02±0.09  (*p*>0.9999) | 0.90±0.21  (*p*=0.9953) | 0.84±0.26  (*p*=0.8954) | 0.83±0.23  (*p*=0.8598) | 0.72±0.21  (*p*=0.3916) | 0.72±0.15  (*p*=0.3743) | 0.79±0.05  (*p*=0.6818) | 0.61±0.03  (*p*=0.1032) | 0.73±0.08  (*p*=0.4215) | 0.81±0.15  (*p*=0.7963) | 0.75±0.08  (*p*=0.5216) | 0.68±0.07  (*p*=0.2580) |
| **CYP21A2** | progesterone/11-deoxycorticosterone | 0.98±0.03  (*p*>0.9999) | 0.91±0.11  (*p*>0.9999) | 0.83±0.12  (*p*=0.9938) | 0.82±0.11  (*p*=0.9906) | 0.84±0.15  (*p*=0.9962) | 0.88±0.12  (*p*=0.9999) | 0.69±0.05  (*p*=0.8142) | 0.40±0.02  (*p*=0.1350) | 1.00±0.48  (*p*>0.9999) | 0.58±0.29  (*p*=0.4969) | 0.63±0.37  (*p*=0.6320) | 0.75±0.49  (*p*=0.9357) |
| **CYP21A2** | 17-OH-progesterone/11-deoxycortisol | 1.01±0.05  (*p*>0.9999) | 0.92±0.15  (*p*>0.9999) | 0.83±0.21  (*p*=0.9871) | 0.84±0.20  (*p*=0.9934) | 0.89±0.26  (*p*=0.9998) | 0.88±0.19  (*p*=0.9994) | 0.93±0.02  (*p*>0.9999) | 0.46±0.00  (*p*=0.1421) | 0.94±0.36  (*p*>0.9999) | 0.54±0.23  (*p*=0.2734) | 0.58±0.29  (*p*=0.3708) | 0.65±0.38  (*p*=0.5911) |
| **CYP21A2** | 21-deoxycortisol/cortisol | 0.98±0.08  (*p*>0.9999) | 1.12±0.11  (*p*=0.9996) | 1.02±0.09  (*p*>0.9999) | 1.13±0.12  (*p*=0.9994) | **1.72±0.55**  **(*p*=0.0357)** | 1.56±0.32  (*p*=0.1526) | **2.52±0.06**  **(*p*<0.0001)** | 1.41±0.08  (*p*=0.4400) | **1.83±0.32**  **(*p*=0.0114)** | 0.88±0.12  (*p*=0.9995) | 0.99±0.09  (*p*>0.9999) | 1.13±0.31  (*p*=0.9993) |
| **HSD3B2** | DHEA/androstenedione | 1.01±0.04  (*p*>0.9999) | 1.11±0.14  (*p*>0.9999) | 1.14±0.16  (*p*>0.9999) | 1.10±0.19  (*p*>0.9999) | 1.27±0.24  (*p*=0.9934) | 1.17±0.26  (*p*=0.9999) | 1.00±0.08  (*p*>0.9999) | 1.12±0.04  (*p*>0.9999) | 1.53±0.38  (*p*=0.6957) | 1.71±0.68  (*p*=0.3690) | 1.85±0.68  (*p*=0.1868) | 1.68±0.67  (*p*=0.4076) |
| **HSD11B1/2** | cortisol/cortisone | 1.04±0.08  (*p*>0.9999) | 1.15±0.02  (*p*>0.9999) | 1.11±0.07  (*p*>0.9999) | 1.23±0.05  (*p*>0.9999) | 1.72±0.54  (*p*=0.7992) | 1.55±0.30  (*p*=0.9476) | **3.27±0.20**  **(*p*=0.0038)** | **4.02±0.11**  **(*p*=0.0001)** | 2.23±1.01  (*p*=0.2338) | 2.16±1.00  (*p*=0.2967) | 2.31±1.13  (*p*=0.1846) | 1.91±0.55  (*p*=0.5615) |
| **HSD17B3** | androstenedione/testosterone | 1.01±0.01  (*p*>0.9999) | 1.02±0.04  (*p*>0.9999) | 0.99±0.05  (*p*>0.9999) | 1.01±0.07  (*p*>0.9999) | 1.02±0.07  (*p*>0.9999) | 0.98±0.03  (*p*>0.9999) | 1.04±0.04  (*p*=0.9972) | 1.09±0.04  (*p*=0.6910) | 1.12±0.09  (*p*=0.4266) | 1.07±0.08  (*p*=0.8739) | 1.06±0.08  (*p*=0.9724) | 1.04±0.12  (*p*=0.9992) |
| **SULT2A1** | DHEA/DHEAS | 1.02±0.08  (*p*>0.9999) | 1.02±0.33  (*p*>0.9999) | 0.98±0.38  (*p*>0.9999) | 1.00±0.38  (*p*>0.9999) | 1.09±0.45  (*p*>0.9999) | 0.95±0.34  (*p*>0.9999) | 1.10±0.12  (*p*>0.9999) | 1.06±0.08  (*p*>0.9999) | 1.53±0.46  (*p*=0.8198) | 1.69±0.71  (*p*=0.5731) | 1.77±0.68  (*p*=0.4405) | 1.48±0.66  (*p*=0.8936) |
| **5α-reductase** | testosterone/DHT | 1.00±0.03  (*p*>0.9999) | 1.15±0.13  (*p*>0.9999) | 1.32±0.32  (*p*=0.9595) | 1.32±0.32  (*p*=0.9609) | 1.45±0.36  (*p*=0.7698) | 1.41±0.29  (*p*=0.8373) | 1.25±0.05  (*p*=0.9923) | 1.86±0.05  (*p*=0.1220) | 1.65±0.39  (*p*=0.3718) | **3.92±0.90**  **(*p*<0.0001)** | **3.41±0.23**  **(*p*<0.0001)** | **2.44±0.15**  **(*p*=0.0022)** |

| **Mixture**  **(DEHP, DiBP,**  **DiNP, DEHA,**  **DEHT, DINCH)** | **calculated steroid ratio** | **1 nM** | **50 nM** | **100 nM** | **250 nM** | **500 nM** | **1 µM** | **2.5 µM** | **5 µM** | **10 µM** | **25 µM** | **50 µM** | **100 µM** |
| --- | --- | --- | --- | --- | --- | --- | --- | --- | --- | --- | --- | --- | --- |
| **CYP11B1** | 17-OH-progesterone/21-deoxycortisol | 1.00±0.02  (*p*>0.9999) | 1.01±0.01  (*p*>0.9999) | 1.04±0.05  (*p*>0.9999) | 0.75±0.13  (*p*=0.8281) | 0.59±0.17  (*p*=0.2936) | 0.51±0.13  (*p*=0.1373) | **0.27±0.02**  **(*p*=0.0091)** | **0.17±0.01**  **(*p*=0.0028)** | 0.42±0.44  (*p*=0.0543) | **0.39±0.38**  **(*p*=0.0416)** | **0.33±0.26**  **(*p*=0.0194)** | **0.25±0.20**  **(*p*=0.0075)** |
| **CYP11B1** | 11-deoxycortisol/cortisol | 0.99±0.03  (*p*>0.9999) | 1.01±0.02  (*p*>0.9999) | 1.07±0.05  (*p*>0.9999) | 0.97±0.14  (*p*>0.9999) | 0.85±0.16  (*p*=0.9396) | 0.88±0.09  (*p*=0.9846) | 0.61±0.03  (*p*=0.0995) | **0.45±0.01**  **(*p*=0.0049)** | 0.62±0.31  (*p*=0.1099) | 0.64±0.26  (*p*=0.1392) | 0.63±0.18  (*p*=0.1264) | **0.49±0.14**  **(*p*=0.0159)** |
| **CYP11B1** | 11-deoxycorticosterone/corticosterone | 0.95±0.08  (*p*>0.9999) | 0.98±0.07  (*p*>0.9999) | 1.02±0.07  (*p*>0.9999) | 0.73±0.15  (*p*=0.8226) | 0.60±0.16  (*p*=0.4359) | 0.49±0.21  (*p*=0.1838) | 0.38±0.02  (*p*=0.0681) | **0.26±0.01**  **(*p*=0.0190)** | 0.49±0.45  (*p*=0.1886) | 0.48±0.41  (*p*=0.1649) | 0.45±0.32  (*p*=0.1293) | **0.30±0.19**  **(*p*=0.0311)** |
| **CYP11B2** | corticosterone/aldosterone | 1.01±0.06  (*p*>0.9999) | 0.94±0.05  (*p*>0.9999) | 1.07±0.09  (*p*>0.9999) | 1.77±0.59  (*p*=0.7360) | 1.78±0.65  (*p*=0.7257) | 2.28±0.96  (*p*=0.1954) | 1.49±0.22  (*p*=0.9738) | 1.30±0.18  (*p*=0.9995) | 1.73±0.60  (*p*=0.7807) | 1.87±0.76  (*p*=0.6074) | 1.97±0.88  (*p*=0.4880) | 1.69±0.71  (*p*=0.8231) |
| **CYP17A1** | progesterone/17-OH-progesterone | 0.99±0.04  (*p*>0.9999) | 1.04±0.06  (*p*=0.9997) | 1.01±0.05  (*p*>0.9999) | 1.01±0.07  (*p*>0.9999) | 1.01±0.04  (*p*>0.9999) | **0.67±0.12**  **(*p*=0.0031)** | 1.00±0.06  (*p*>0.9999) | 1.11±0.05  (*p*=0.7441) | 1.08±0.11  (*p*=0.9302) | 1.10±0.09  (*p*=0.8037) | 1.18±0.12  (*p*=0.2344) | **1.26±0.13**  **(*p*=0.0255)** |
| **CYP17A1** | 17-OH-progesterone/androstenedione | 0.95±0.02  (*p*=0.9997) | 0.94±0.02  (*p*=0.9989) | 0.91±0.04  (*p*=0.9737) | 0.89±0.04  (*p*=0.9187) | 0.83±0.07  (*p*=0.5225) | 0.72±0.06  (*p*=0.0904) | 0.95±0.02  (*p*>0.9999) | 0.99±0.08  (*p*>0.9999) | 0.76±0.12  (*p*=0.2039) | **0.63±0.18**  **(*p*=0.0114)** | **0.55±0.24**  **(*p*=0.0018)** | 0.91±0.13  (*p*=0.9764) |
| **CYP19A1** | testosterone/estradiol | 0.97±0.11  (*p*>0.9999) | 0.92±0.16  (*p*=0.9999) | 0.94±0.18  (*p*>0.9999) | 0.85±0.03  (*p*=0.9742) | 0.82±0.02  (*p*=0.9397) | 0.80±0.01  (*p*=0.8692) | 0.87±0.10  (*p*=0.9904) | 0.71±0.05  (*p*=0.5354) | 0.76±0.28  (*p*=0.7418) | 0.72±0.29  (*p*=0.5711) | 0.74±0.36  (*p*=0.6788) | 0.70±0.15  (*p*=0.4960) |
| **CYP21A2** | progesterone/11-deoxycorticosterone | 0.95±0.03  (*p*>0.9999) | 0.97±0.04  (*p*>0.9999) | 0.91±0.03  (*p*=0.9853) | 0.87±0.07  (*p*=0.8957) | 0.82±0.18  (*p*=0.6052) | **0.51±0.09**  **(*p*=0.0018)** | **0.62±0.04**  **(*p*=0.0198)** | 0.73±0.08  (*p*=0.1574) | 0.77±0.08  (*p*=0.3074) | **0.63±0.15**  **(*p*=0.0258)** | **0.58±0.19**  **(*p*=0.0091)** | 0.98±0.20  (*p*>0.9999) |
| **CYP21A2** | 17-OH-progesterone/11-deoxycortisol | 0.98±0.06  (*p*>0.9999) | 0.98±0.03  (*p*>0.9999) | 0.93±0.06  (*p*=0.9987) | 0.89±0.05  (*p*=0.9489) | 0.84±0.02  (*p*=0.7068) | 0.71±0.03  (*p*=0.1245) | 0.91±0.01  (*p*=0.9870) | 0.96±0.02  (*p*>0.9999) | 0.73±0.18  (*p*=0.1818) | **0.60±0.23**  **(*p*=0.0173)** | **0.52±0.25**  **(*p*=0.0029)** | 0.82±0.12  (*p*=0.6256) |
| **CYP21A2** | 21-deoxycortisol/cortisol | 0.97±0.07  (*p*>0.9999) | 0.97±0.04  (*p*>0.9999) | 0.96±0.08  (*p*>0.9999) | 1.15±0.08  (*p*>0.9999) | 1.24±0.17  (*p*=0.9925) | 1.27±0.16  (*p*=0.9828) | **2.11±0.10**  **(*p*=0.0213)** | **2.51±0.13**  **(*p*=0.0010)** | 1.86±0.63  (*p*=0.1116) | 1.42±0.35  (*p*=0.8139) | 1.19±0.14  (*p*=0.9991) | **2.33±0.88**  **(*p*=0.0041)** |
| **HSD3B2** | DHEA/androstenedione | 1.12±0.09  (*p*=0.6526) | 1.09±0.10  (*p*=0.8802) | 1.06±0.05  (*p*=0.9860) | 1.04±0.07  (*p*>0.9999) | 1.04±0.08  (*p*=0.9992) | 0.99±0.14  (*p*>0.9999) | 1.01±0.02  (*p*>0.9999) | 1.05±0.03  (*p*=0.9990) | 1.09±0.07  (*p*=0.8934) | 1.12±0.09  (*p*=0.6333) | 0.99±0.04  (*p*>0.9999) | 1.19±0.10  (*p*=0.1663) |
| **HSD11B1/2** | cortisol/cortisone | 0.99±0.04  (*p*>0.9999) | 0.99±0.07  (*p*>0.9999) | 1.02±0.04  (*p*>0.9999) | 1.41±0.19  (*p*=0.9993) | 1.72±0.27  (*p*=0.9442) | 1.89±0.32  (*p*=0.8374) | 3.03±0.17  (*p*=0.0728) | **4.12±0.16**  **(*p*=0.0020)** | 2.98±1.43  (*p*=0.0838) | 2.99±1.32  (*p*=0.0816) | 2.85±1.04  (*p*=0.1227) | **3.51±1.30**  **(*p*=0.0167)** |
| **HSD17B3** | androstenedione/testosterone | 1.03±0.01  (*p*>0.9999) | 1.04±0.01  (*p*>0.9999) | 1.05±0.05  (*p*=0.9997) | 1.14±0.07  (*p*=0.6622) | 1.17±0.11  (*p*=0.3831) | 1.15±0.03  (*p*=0.5733) | 1.09±0.02  (*p*=0.9387) | 1.13±0.05  (*p*=0.6977) | 1.15±0.13  (*p*=0.5231) | 1.19±0.14  (*p*=0.3208) | 1.19±0.18  (*p*=0.3014) | 1.20±0.13  (*p*=0.2459) |
| **SULT2A1** | DHEA/DHEAS | 1.16±0.06  (*p*=0.4515) | 1.13±0.07  (*p*=0.6811) | 1.15±0.06  (*p*=0.5523) | 1.22±0.13  (*p*=0.1550) | 1.22±0.18  (*p*=0.1712) | 1.20±0.03  (*p*=0.2501) | 1.16±0.02  (*p*=0.4694) | 1.17±0.06  (*p*=0.4266) | 1.21±0.13  (*p*=0.2069) | **1.28±0.05**  **(*p*=0.0402)** | 1.11±0.02  (*p*=0.8356) | **1.37±0.15**  **(*p*=0.0037)** |
| **5α-reductase** | testosterone/DHT | 1.05±0.01  (*p*>0.9999) | 1.05±0.02  (*p*>0.9999) | 1.07±0.01  (*p*>0.9999) | 1.07±0.05  (*p*>0.9999) | 1.09±0.06  (*p*>0.9999) | 1.08±0.22  (*p*>0.9999) | 1.23±0.05  (*p*=0.9239) | 1.26±0.07  (*p*=0.8761) | 1.40±0.25  (*p*=0.4539) | 1.64±0.38  (*p*=0.0595) | **1.91±0.60**  **(*p*=0.0036)** | 1.22±0.12  (*p*=0.9487) |
