# Supplementary material for: Disruptive effects of phthalates and their substitutes on adrenal steroidogenesis
Source: Front Endocrinol (Lausanne). 2026 Jan 14;16:1734184. doi: 10.3389/fendo.2025.1734184 (PMC12848149; doi:10.3389/fendo.2025.1734184)
Supplement: Supplementary file 5 [file DataSheet5.docx]

**Supplementary Material S5.** Gene expression of various steroidogenic genes, measured by real time quantitative polymerase chain reaction. Data are presented as fold-changes compared to vehicle-treated controls (n = 3; mean ± SD**).** Respective *p*-values are presented for each data point, while statistically significant values are marked in bold and grey background.

| **Mixture**  **(DEHP, DiBP, DiNP, DEHA, DEHT, DINCH)** | **250 nM** | **500 nM** | **1 µM** | **2.5 µM** | **5 µM** | **10 µM** | **25 µM** |
| --- | --- | --- | --- | --- | --- | --- | --- |
| ***STAR*** | 1.06±0.33  (*p*>0.9999) | 0.98±0.19  (*p*>0.9999) | 1.36±0.60  (*p*=0.8891) | 1.53±0.30  (*p*=0.6134) | 1.54±0.75  (*p*=0.6036) | 1.60±0.80  (*p*=0.4912) | 1.62±0.75  (*p*=0.4546) |
| ***CYP11B1*** | 0.94±0.33  (*p*>0.9999) | 1.49±0.60  (*p*=0.9998) | 1.07±0.52  (*p*>0.9999) | **6.65±5.46**  **(*p*=0.0053)** | **7.25±2.49**  **(*p*=0.0017)** | **11.82±4.40**  **(*p*<0.0001)** | **8.09±1.65**  **(*p*=0.0002)** |
| ***CYP11B2*** | 2.1±0.49  (*p*=0.9998) | 2.76±0.72  (*p*=0.9961) | 2.43±0.78  (*p*=0.9989) | **23.85±11.97**  **(*p*<0.0001)** | **35.39±13.30**  **(*p*<0.0001)** | **44.12±9.77**  **(*p*<0.0001)** | **30.22±2.63**  **(*p*<0.0001)** |
| ***CYP17A1*** | **1.49±0.24**  **(*p*=0.0019)** | **1.63±0.24**  **(*p*<0.0001)** | **1.48±0.24**  **(*p*=0.0025)** | **1.88±0.41**  **(*p*<0.0001)** | **1.86±0.36**  **(*p*<0.0001)** | **2.02±0.17**  **(*p*<0.0001)** | **2.10±0.18**  **(*p*<0.0001)** |
| ***CYP21A2*** | 1.28±0.09  (*p*=0.5147) | 1.33±0.15  (*p*=0.3490) | 1.32±0.12  (*p*=0.3765) | **2.03±0.74**  **(*p*<0.0001)** | **2.28±0.42**  **(*p*<0.0001)** | **1.65±0.42**  **(*p*=0.0028)** | 1.34±0.28  (*p*=0.2398) |
| ***HSD3B2*** | 1.20±0.31  (*p*=0.9890) | 1.54±0.52  (*p*=0.4712) | 1.38±0.40  (*p*=0.7915) | **2.72±0.95**  **(*p*<0.0001)** | **3.80±0.83**  **(*p*<0.0001)** | **4.26±0.87**  **(*p*<0.0001)** | **3.57±1.28**  **(*p*<0.0001)** |
| ***AGTR1*** | 1.08±0.07  (*p*=0.9533) | 1.08±0.09  (*p*=0.9509) | 1.11±0.08  (*p*=0.7559) | **2.08±0.30**  **(*p*<0.0001)** | **1.86±0.38**  **(*p*<0.0001)** | 0.94±0.14  (*p*>0.9999) | 1.28±0.18  (*p*=0.0731) |
| ***MC2R*** | 1.24±0.17  (*p*=0.9997) | 1.53±0.29  (*p*=0.9640) | 1.25±0.24  (*p*=0.9996) | **7.03±2.24**  **(*p*<0.0001)** | **7.04±2.53**  **(*p*<0.0001)** | **6.57±0.53**  **(*p*<0.0001)** | **7.06±0.48**  **(*p*<0.0001)** |
| ***SF-1*** | 0.88±0.30  (*p*=0.9991) | 0.90±0.35  (*p*=0.9997) | 1.05±0.49  (*p*>0.9999) | 1.10±0.37  (*p*=0.9997) | 1.73±1.05  (*p*=0.1079) | 0.98±0.19  (*p*>0.9999) | 1.00±0.27  (*p*>0.9999) |
