# Supplementary material for: Disruptive effects of phthalates and their substitutes on adrenal steroidogenesis
Source: Front Endocrinol (Lausanne). 2026 Jan 14;16:1734184. doi: 10.3389/fendo.2025.1734184 (PMC12848149; doi:10.3389/fendo.2025.1734184)
Supplement: Supplementary file 6 [file DataSheet6.docx]

| **DEHP_1**  **steroid**  **concentration** | aldosterone | androstenedione | cortisol | cortisone | corticosterone | 11-deoxycorticosterone | 11-deoxycortisol | 21-deoxycortisol | DHEA | DHEAS | DHT | estradiol | 17-OH-P | progesterone | testosterone |
| --- | --- | --- | --- | --- | --- | --- | --- | --- | --- | --- | --- | --- | --- | --- | --- |
| 1% DMSO | 0.034 | 41.0 | 13.9 | 1.000 | 0.79 | 1.48 | 68.6 | 0.285 | 10.6 | 140.0 | 0.399 | 0.090 | 36.3 | 1.020 | 2.07 |
| 1 nM | 0.034 | 43.9 | 14.2 | 1.020 | 0.89 | 1.66 | 74.1 | 0.306 | 12.3 | 150.0 | 0.387 | 0.097 | 35.2 | 0.934 | 2.11 |
| 50 nM | 0.032 | 42.6 | 14.1 | 1.060 | 0.84 | 1.62 | 74.1 | 0.312 | 12.5 | 145.0 | 0.399 | 0.094 | 36.4 | 0.953 | 2.13 |
| 100 nM | 0.036 | 42.7 | 14.6 | 1.020 | 0.97 | 1.67 | 75.4 | 0.331 | 12.0 | 143.0 | 0.400 | 0.094 | 37.2 | 1.000 | 2.15 |
| 250 nM | 0.037 | 42.8 | 14.9 | 0.912 | 1.04 | 1.68 | 73.0 | 0.343 | 13.0 | 138.0 | 0.379 | 0.097 | 36.7 | 1.010 | 2.09 |
| 500 nM | 0.042 | 43.6 | 17.7 | 0.985 | 1.21 | 1.69 | 78.3 | 0.415 | 12.5 | 150.0 | 0.402 | 0.104 | 37.3 | 1.020 | 2.22 |
| 1 µM | 0.045 | 42.1 | 18.8 | 0.935 | 1.44 | 1.79 | 79.5 | 0.499 | 11.5 | 143.0 | 0.386 | 0.106 | 37.3 | 1.050 | 2.13 |
| 2.5 µM | 0.178 | 40.8 | 43.0 | 0.754 | 5.42 | 2.27 | 84.5 | 2.138 | 9.9 | 152.4 | 0.338 | 0.121 | 38.8 | 1.384 | 2.05 |
| 5 µM | 0.230 | 41.8 | 48.8 | 0.728 | 6.67 | 2.42 | 86.5 | 2.833 | 11.2 | 141.8 | 0.286 | 0.140 | 37.4 | 1.361 | 1.93 |
| 10 µM | 0.036 | 36.9 | 16.3 | 0.757 | 1.21 | 1.33 | 70.9 | 0.393 | 11.5 | 131.0 | 0.308 | 0.122 | 30.9 | 1.050 | 1.77 |
| 25 µM | 0.040 | 26.1 | 18.0 | 0.829 | 0.97 | 0.83 | 50.5 | 0.319 | 7.9 | 138.2 | 0.123 | 0.140 | 16.7 | 0.534 | 1.23 |
| 50 µM | 0.037 | 26.5 | 18.4 | 0.829 | 0.88 | 0.83 | 52.9 | 0.276 | 8.7 | 143.8 | 0.094 | 0.154 | 12.8 | 0.371 | 1.15 |
| 100 µM | 0.030 | 30.1 | 11.9 | 0.691 | 0.76 | 0.69 | 61.1 | 0.167 | 6.1 | 115.0 | 0.175 | 0.134 | 13.7 | 0.333 | 1.61 |

**Supplementary Material S6.** Absolute concentrations of all LCMS/MS measurements in the three independent biological replicates are reported in µg/L.

| **DEHP_2**  **steroid**  **concentration** | aldosterone | androstenedione | cortisol | cortisone | corticosterone | 11-deoxycorticosterone | 11-deoxycortisol | 21-deoxycortisol | DHEA | DHEAS | DHT | estradiol | 17-OH-P | progesterone | testosterone |
| --- | --- | --- | --- | --- | --- | --- | --- | --- | --- | --- | --- | --- | --- | --- | --- |
| 1% DMSO | 0.033 | 90.3 | 20.4 | 1.840 | 0.54 | 1.52 | 153.0 | 0.372 | 14.5 | 291.0 | 0.632 | 0.264 | 68.8 | 0.936 | 4.63 |
| 1 nM | 0.034 | 86.0 | 19.9 | 1.900 | 0.58 | 1.50 | 154.0 | 0.433 | 15.3 | 274.0 | 0.613 | 0.241 | 70.6 | 0.869 | 4.42 |
| 50 nM | 0.032 | 86.1 | 20.4 | 1.900 | 0.56 | 1.54 | 151.0 | 0.401 | 17.0 | 275.0 | 0.635 | 0.253 | 72.0 | 1.010 | 4.58 |
| 100 nM | 0.036 | 92.4 | 22.0 | 1.860 | 0.62 | 1.54 | 163.0 | 0.436 | 20.2 | 297.0 | 0.629 | 0.282 | 70.9 | 0.905 | 4.84 |
| 250 nM | 0.037 | 80.3 | 20.7 | 1.750 | 0.62 | 1.49 | 146.0 | 0.451 | 12.7 | 250.0 | 0.640 | 0.235 | 75.1 | 1.040 | 4.15 |
| 500 nM | 0.038 | 77.0 | 21.5 | 1.580 | 0.75 | 1.56 | 145.0 | 0.498 | 12.2 | 233.0 | 0.606 | 0.250 | 72.9 | 1.090 | 3.95 |
| 1 µM | 0.041 | 77.1 | 25.0 | 1.620 | 0.92 | 1.63 | 153.0 | 0.630 | 12.2 | 240.0 | 0.603 | 0.255 | 76.9 | 1.120 | 4.06 |
| 2.5 µM | 0.136 | 86.8 | 52.6 | 1.296 | 2.62 | 2.12 | 171.3 | 2.172 | 12.8 | 296.2 | 0.498 | 0.342 | 71.8 | 1.244 | 4.31 |
| 5 µM | 0.168 | 82.4 | 56.7 | 1.321 | 2.93 | 1.91 | 167.8 | 2.967 | 13.9 | 288.5 | 0.432 | 0.333 | 70.6 | 1.167 | 4.01 |
| 10 µM | 0.040 | 64.9 | 26.3 | 1.370 | 0.95 | 1.25 | 133.0 | 0.701 | 11.5 | 220.0 | 0.427 | 0.292 | 61.6 | 1.180 | 3.13 |
| 25 µM | 0.033 | 63.3 | 29.1 | 1.369 | 0.94 | 0.81 | 117.7 | 0.348 | 9.1 | 252.0 | 0.263 | 0.382 | 25.1 | 0.446 | 3.01 |
| 50 µM | 0.027 | 60.8 | 24.2 | 1.318 | 0.86 | 0.75 | 112.3 | 0.229 | 8.7 | 245.7 | 0.207 | 0.376 | 17.6 | 0.283 | 3.01 |
| 100 µM | 0.032 | 53.5 | 18.8 | 1.260 | 0.58 | 0.71 | 119.0 | 0.279 | 7.3 | 193.0 | 0.215 | 0.321 | 26.9 | 0.388 | 2.71 |

| **DEHP_3**  **steroid**  **concentration** | aldosterone | androstenedione | cortisol | cortisone | corticosterone | 11-deoxycorticosterone | 11-deoxycortisol | 21-deoxycortisol | DHEA | DHEAS | DHT | estradiol | 17-OH-P | progesterone | testosterone |
| --- | --- | --- | --- | --- | --- | --- | --- | --- | --- | --- | --- | --- | --- | --- | --- |
| 1% DMSO | 0.041 | 78.0 | 30.3 | 3.430 | 0.76 | 8.08 | 338.0 | 0.079 | 3.3 | 145.0 | 0.419 | 0.311 | 57.5 | 2.500 | 3.83 |
| 1 nM | 0.042 | 78.8 | 32.1 | 3.510 | 0.74 | 6.17 | 311.0 | 0.072 | 3.0 | 155.0 | 0.400 | 0.314 | 56.9 | 2.180 | 3.98 |
| 50 nM | 0.038 | 80.5 | 30.0 | 3.200 | 0.72 | 6.86 | 313.0 | 0.062 | 2.9 | 145.0 | 0.425 | 0.303 | 59.4 | 2.340 | 4.04 |
| 100 nM | 0.041 | 82.6 | 30.8 | 3.010 | 1.01 | 7.72 | 327.0 | 0.093 | 3.7 | 136.0 | 0.423 | 0.316 | 59.4 | 2.440 | 3.93 |
| 250 nM | 0.056 | 88.0 | 35.0 | 2.530 | 1.48 | 8.30 | 337.0 | 0.125 | 3.8 | 138.0 | 0.426 | 0.338 | 60.7 | 2.480 | 4.11 |
| 500 nM | 0.072 | 83.1 | 36.0 | 1.690 | 2.71 | 10.40 | 331.0 | 0.151 | 3.8 | 108.0 | 0.348 | 0.332 | 51.7 | 2.680 | 3.51 |
| 1 µM | 0.108 | 83.3 | 44.1 | 1.760 | 3.69 | 11.10 | 353.0 | 0.228 | 3.7 | 109.0 | 0.339 | 0.351 | 45.9 | 2.580 | 3.51 |
| 2.5 µM | 0.215 | 77.0 | 87.7 | 2.272 | 5.61 | 13.41 | 405.1 | 0.646 | 3.2 | 137.8 | 0.347 | 0.405 | 68.4 | 3.740 | 3.64 |
| 5 µM | 0.224 | 74.9 | 93.0 | 2.238 | 5.07 | 8.79 | 330.7 | 0.611 | 3.0 | 160.2 | 0.297 | 0.424 | 62.6 | 3.171 | 3.47 |
| 10 µM | 0.095 | 67.6 | 44.7 | 1.670 | 2.92 | 7.67 | 286.0 | 0.216 | 2.7 | 99.4 | 0.169 | 0.415 | 29.2 | 1.830 | 2.60 |
| 25 µM | 0.043 | 58.9 | 49.2 | 2.781 | 1.52 | 4.90 | 269.5 | 0.112 | 2.5 | 141.6 | 0.201 | 0.452 | 26.6 | 1.445 | 2.76 |
| 50 µM | 0.036 | 55.5 | 40.8 | 2.658 | 1.45 | 4.75 | 264.7 | 0.072 | 2.2 | 130.3 | 0.164 | 0.439 | 20.0 | 1.159 | 2.58 |
| 100 µM | 0.056 | 46.9 | 27.4 | 1.550 | 1.28 | 3.65 | 217.0 | 0.036 | 1.3 | 72.3 | 0.056 | 0.393 | 9.9 | 0.650 | 1.83 |

| **DiBP_1**  **steroid**  **concentration** | aldosterone | androstenedione | cortisol | cortisone | corticosterone | 11-deoxycorticosterone | 11-deoxycortisol | 21-deoxycortisol | DHEA | DHEAS | DHT | estradiol | 17-OH-P | progesterone | testosterone |
| --- | --- | --- | --- | --- | --- | --- | --- | --- | --- | --- | --- | --- | --- | --- | --- |
| 1% DMSO | 5.370 | 22.0 | 160.0 | 1.030 | 58.50 | 36.20 | 271.0 | 1.990 | 2.6 | 44.1 | 0.271 | 0.130 | 25.5 | 7.48 | 2.91 |
| 1 nM | 4.950 | 24.5 | 150.0 | 1.010 | 57.60 | 39.90 | 284.0 | 1.930 | 2.8 | 42.8 | 0.284 | 0.125 | 31.4 | 9.46 | 3.17 |
| 50 nM | 5.800 | 22.9 | 162.0 | 1.050 | 60.40 | 38.10 | 274.0 | 2.020 | 2.9 | 43.8 | 0.274 | 0.133 | 26.6 | 7.97 | 2.92 |
| 100 nM | 5.690 | 22.9 | 165.0 | 1.080 | 61.30 | 37.90 | 281.0 | 2.090 | 2.9 | 44.7 | 0.281 | 0.131 | 26.6 | 7.92 | 2.98 |
| 250 nM | 5.260 | 24.7 | 157.0 | 1.040 | 59.60 | 40.90 | 292.0 | 2.090 | 3.0 | 42.8 | 0.292 | 0.129 | 30.7 | 9.09 | 3.19 |
| 500 nM | 4.570 | 29.7 | 137.0 | 0.916 | 56.30 | 42.40 | 283.0 | 1.920 | 3.7 | 44.2 | 0.283 | 0.107 | 40.4 | 11.10 | 3.39 |
| 1 µM | 4.330 | 30.4 | 134.0 | 0.894 | 56.90 | 43.70 | 286.0 | 1.860 | 3.6 | 41.8 | 0.286 | 0.104 | 41.9 | 12.10 | 3.41 |
| 2.5 µM | 5.354 | 23.2 | 176.8 | 1.045 | 66.04 | 39.87 | 264.5 | 2.259 | 2.9 | 40.2 | 0.264 | 0.142 | 25.9 | 7.48 | 3.10 |
| 5 µM | 5.233 | 25.9 | 156.0 | 0.864 | 68.36 | 44.32 | 261.3 | 2.086 | 3.2 | 35.8 | 0.261 | 0.130 | 30.4 | 8.95 | 3.29 |
| 10 µM | 5.290 | 30.8 | 146.0 | 0.878 | 67.30 | 48.10 | 294.0 | 2.020 | 3.7 | 42.8 | 0.294 | 0.117 | 39.2 | 12.40 | 3.53 |
| 25 µM | 6.690 | 23.5 | 165.0 | 0.921 | 71.40 | 38.30 | 265.0 | 2.310 | 2.9 | 42.4 | 0.265 | 0.131 | 24.9 | 8.08 | 2.94 |
| 50 µM | 7.080 | 23.2 | 178.0 | 0.993 | 72.30 | 34.30 | 264.0 | 2.190 | 2.8 | 49.5 | 0.264 | 0.146 | 20.5 | 6.92 | 3.16 |
| 100 µM | 6.110 | 28.8 | 175.0 | 1.010 | 64.80 | 31.60 | 288.0 | 1.610 | 2.4 | 58.0 | 0.288 | 0.163 | 15.0 | 4.91 | 4.29 |

| **DiBP_2**  **steroid concentration** | aldosterone | androstenedione | cortisol | cortisone | corticosterone | 11-deoxycorticosterone | 11-deoxycortisol | 21-deoxycortisol | DHEA | DHEAS | DHT | estradiol | 17-OH-P | progesterone | testosterone |
| --- | --- | --- | --- | --- | --- | --- | --- | --- | --- | --- | --- | --- | --- | --- | --- |
| 1% DMSO | 5.740 | 24.0 | 163.0 | 1.020 | 63.30 | 40.40 | 280.0 | 1.810 | 2.6 | 45.3 | 0.280 | 0.133 | 27.6 | 9.48 | 3.05 |
| 1 nM | 5.830 | 27.5 | 159.0 | 1.010 | 66.50 | 44.00 | 299.0 | 2.150 | 3.3 | 46.1 | 0.299 | 0.126 | 35.6 | 11.10 | 3.36 |
| 50 nM | 6.010 | 26.7 | 158.0 | 0.995 | 67.00 | 44.00 | 294.0 | 2.110 | 3.5 | 43.8 | 0.294 | 0.127 | 34.1 | 10.50 | 3.15 |
| 100 nM | 5.750 | 25.5 | 153.0 | 0.955 | 65.00 | 42.60 | 283.0 | 2.010 | 3.3 | 43.3 | 0.283 | 0.123 | 31.9 | 10.10 | 3.10 |
| 250 nM | 6.100 | 25.1 | 163.0 | 1.020 | 66.00 | 41.30 | 286.0 | 2.050 | 3.1 | 44.3 | 0.286 | 0.130 | 30.1 | 9.56 | 3.06 |
| 500 nM | 5.670 | 26.8 | 151.0 | 0.948 | 65.30 | 43.20 | 283.0 | 2.010 | 3.6 | 42.6 | 0.283 | 0.124 | 33.7 | 10.70 | 3.13 |
| 1 µM | 6.270 | 25.3 | 159.0 | 0.971 | 68.70 | 43.80 | 278.0 | 2.030 | 3.1 | 42.8 | 0.278 | 0.127 | 30.1 | 9.99 | 2.99 |
| 2.5 µM | 4.716 | 24.5 | 153.8 | 0.919 | 63.77 | 43.88 | 254.9 | 1.920 | 2.8 | 45.7 | 0.255 | 0.128 | 27.9 | 9.61 | 2.99 |
| 5 µM | 5.249 | 29.1 | 161.0 | 0.832 | 74.36 | 47.54 | 314.6 | 2.215 | 3.6 | 49.8 | 0.315 | 0.131 | 35.7 | 11.18 | 3.41 |
| 10 µM | 7.170 | 24.7 | 169.0 | 0.935 | 74.80 | 41.20 | 273.0 | 2.180 | 3.0 | 42.9 | 0.273 | 0.133 | 27.5 | 9.11 | 3.04 |
| 25 µM | 7.640 | 24.9 | 166.0 | 0.891 | 78.20 | 42.70 | 271.0 | 2.240 | 3.1 | 41.8 | 0.271 | 0.129 | 26.6 | 9.22 | 3.00 |
| 50 µM | 7.520 | 25.9 | 157.0 | 0.842 | 79.00 | 42.40 | 272.0 | 2.270 | 3.0 | 41.2 | 0.272 | 0.129 | 26.2 | 9.77 | 3.21 |
| 100 µM | 6.050 | 33.4 | 151.0 | 0.846 | 73.00 | 43.70 | 290.0 | 1.700 | 2.6 | 48.4 | 0.290 | 0.122 | 23.7 | 9.87 | 4.33 |

| **DiBP_3**  **steroid concentration** | aldosterone | androstenedione | cortisol | cortisone | corticosterone | 11-deoxycorticosterone | 11-deoxycortisol | 21-deoxycortisol | DHEA | DHEAS | DHT | estradiol | 17-OH-P | progesterone | testosterone |
| --- | --- | --- | --- | --- | --- | --- | --- | --- | --- | --- | --- | --- | --- | --- | --- |
| 1% DMSO | 0.073 | 16.0 | 12.2 | 0.548 | 2.28 | 3.25 | 55.0 | 0.227 | 14.4 | 43.9 | 0.055 | 0.018 | 15.4 | 1.060 | 1.25 |
| 1 nM | 0.069 | 16.0 | 11.5 | 0.527 | 2.20 | 3.11 | 51.9 | 0.221 | 14.6 | 42.1 | 0.052 | 0.017 | 14.9 | 0.966 | 1.19 |
| 50 nM | 0.048 | 15.6 | 8.4 | 0.444 | 1.62 | 2.72 | 42.3 | 0.180 | 16.8 | 36.2 | 0.042 | 0.016 | 13.5 | 0.846 | 0.98 |
| 100 nM | 0.040 | 14.7 | 7.3 | 0.409 | 1.34 | 2.32 | 36.9 | 0.166 | 15.1 | 30.8 | 0.037 | 0.014 | 12.2 | 0.755 | 0.88 |
| 250 nM | 0.046 | 15.7 | 8.2 | 0.412 | 1.59 | 2.64 | 40.1 | 0.176 | 16.3 | 32.7 | 0.040 | 0.013 | 13.5 | 0.826 | 0.94 |
| 500 nM | 0.062 | 15.4 | 10.2 | 0.467 | 1.99 | 2.80 | 43.5 | 0.214 | 16.3 | 34.4 | 0.044 | 0.015 | 14.3 | 0.904 | 1.03 |
| 1 µM | 0.065 | 16.0 | 11.0 | 0.482 | 2.18 | 2.96 | 44.5 | 0.237 | 15.6 | 36.8 | 0.045 | 0.017 | 15.7 | 1.020 | 1.10 |
| 2.5 µM | 0.057 | 16.0 | 11.8 | 0.504 | 2.33 | 3.52 | 48.0 | 0.236 | 13.7 | 43.5 | 0.048 | 0.019 | 16.7 | 1.113 | 1.23 |
| 5 µM | 0.058 | 16.7 | 11.2 | 0.457 | 2.39 | 3.37 | 44.5 | 0.236 | 15.5 | 43.9 | 0.044 | 0.018 | 15.7 | 1.006 | 1.21 |
| 10 µM | 0.061 | 16.2 | 10.0 | 0.436 | 2.03 | 2.72 | 40.4 | 0.215 | 17.1 | 36.3 | 0.040 | 0.015 | 14.4 | 0.902 | 1.03 |
| 25 µM | 0.103 | 16.1 | 15.3 | 0.476 | 3.35 | 3.23 | 50.3 | 0.346 | 16.2 | 42.9 | 0.050 | 0.018 | 16.9 | 1.140 | 1.18 |
| 50 µM | 0.084 | 16.3 | 13.8 | 0.433 | 3.02 | 3.04 | 46.5 | 0.318 | 17.8 | 41.2 | 0.047 | 0.019 | 17.2 | 1.180 | 1.11 |
| 100 µM | 0.080 | 15.4 | 13.1 | 0.355 | 2.73 | 2.68 | 40.4 | 0.357 | 20.5 | 38.4 | 0.040 | 0.017 | 15.2 | 0.946 | 0.97 |

| **DiNP_1**  **steroid concentration** | aldosterone | androstenedione | cortisol | cortisone | corticosterone | 11-deoxycorticosterone | 11-deoxycortisol | 21-deoxycortisol | DHEA | DHEAS | DHT | estradiol | 17-OH-P | progesterone | testosterone |
| --- | --- | --- | --- | --- | --- | --- | --- | --- | --- | --- | --- | --- | --- | --- | --- |
| 1% DMSO | 0.627 | 32.3 | 57.4 | 0.713 | 16.20 | 13.90 | 177.0 | 0.488 | 4.3 | 31.8 | 0.177 | 0.049 | 24.6 | 1.790 | 2.33 |
| 1 nM | 0.591 | 32.6 | 53.7 | 0.670 | 16.30 | 14.90 | 178.0 | 0.503 | 4.1 | 29.9 | 0.178 | 0.047 | 26.2 | 1.870 | 2.27 |
| 50 nM | 0.609 | 32.7 | 52.5 | 0.680 | 16.60 | 15.10 | 183.0 | 0.492 | 4.1 | 29.6 | 0.183 | 0.048 | 26.0 | 1.850 | 2.30 |
| 100 nM | 0.606 | 33.3 | 53.4 | 0.628 | 16.40 | 14.70 | 179.0 | 0.497 | 4.0 | 28.8 | 0.179 | 0.049 | 24.7 | 1.730 | 2.27 |
| 250 nM | 0.661 | 34.8 | 56.3 | 0.569 | 17.40 | 15.00 | 181.0 | 0.509 | 4.1 | 30.9 | 0.181 | 0.052 | 24.0 | 1.640 | 2.41 |
| 500 nM | 0.931 | 32.1 | 65.5 | 0.627 | 20.20 | 15.20 | 187.0 | 0.641 | 3.8 | 32.5 | 0.187 | 0.057 | 22.9 | 1.670 | 2.34 |
| 1 µM | 1.410 | 31.1 | 83.4 | 0.657 | 26.80 | 15.40 | 199.0 | 1.020 | 4.2 | 32.8 | 0.199 | 0.062 | 23.2 | 1.730 | 2.26 |
| 2.5 µM | 3.022 | 33.6 | 163.4 | 0.484 | 100.68 | 19.88 | 208.2 | 3.528 | 4.6 | 25.5 | 0.208 | 0.064 | 26.6 | 1.901 | 2.24 |
| 5 µM | 2.955 | 31.4 | 178.0 | 0.519 | 104.00 | 16.46 | 207.9 | 4.044 | 4.3 | 24.6 | 0.208 | 0.064 | 25.0 | 1.624 | 2.12 |
| 10 µM | 1.240 | 26.5 | 85.5 | 0.671 | 24.00 | 9.44 | 164.0 | 0.883 | 3.8 | 32.3 | 0.164 | 0.066 | 16.2 | 1.120 | 1.82 |
| 25 µM | 1.070 | 26.2 | 83.6 | 0.730 | 22.40 | 7.32 | 161.0 | 0.581 | 3.3 | 34.0 | 0.161 | 0.070 | 12.3 | 0.753 | 1.81 |
| 50 µM | 1.220 | 25.7 | 85.2 | 0.687 | 23.70 | 8.53 | 159.0 | 0.738 | 3.3 | 31.7 | 0.159 | 0.066 | 13.4 | 0.934 | 1.71 |
| 100 µM | 1.120 | 27.3 | 84.7 | 0.705 | 22.90 | 8.34 | 159.0 | 0.653 | 3.6 | 33.5 | 0.159 | 0.068 | 13.2 | 0.890 | 1.82 |

| **DiNP _2**  **steroid concentration** | aldosterone | androstenedione | cortisol | cortisone | corticosterone | 11-deoxycorticosterone | 11-deoxycortisol | 21-deoxycortisol | DHEA | DHEAS | DHT | estradiol | 17-OH-P | progesterone | testosterone |
| --- | --- | --- | --- | --- | --- | --- | --- | --- | --- | --- | --- | --- | --- | --- | --- |
| 1% DMSO | 0.586 | 33.2 | 55.7 | 0.702 | 15.00 | 13.80 | 176.0 | 0.490 | 4.2 | 36.5 | 0.176 | 0.049 | 28.7 | 2.010 | 2.54 |
| 1 nM | 0.579 | 34.8 | 58.0 | 0.781 | 15.20 | 13.80 | 184.0 | 0.504 | 4.1 | 39.2 | 0.184 | 0.049 | 30.5 | 2.170 | 2.73 |
| 50 nM | 0.611 | 36.6 | 58.9 | 0.727 | 15.90 | 14.30 | 191.0 | 0.535 | 5.0 | 38.8 | 0.191 | 0.051 | 30.4 | 1.960 | 2.82 |
| 100 nM | 0.617 | 35.1 | 56.4 | 0.683 | 16.00 | 14.60 | 185.0 | 0.519 | 4.2 | 35.8 | 0.185 | 0.052 | 28.5 | 1.910 | 2.61 |
| 250 nM | 0.604 | 35.2 | 55.2 | 0.589 | 15.90 | 14.40 | 180.0 | 0.523 | 4.4 | 34.6 | 0.180 | 0.050 | 26.2 | 1.750 | 2.57 |
| 500 nM | 0.763 | 35.6 | 64.2 | 0.670 | 18.70 | 15.80 | 198.0 | 0.645 | 4.6 | 36.6 | 0.198 | 0.055 | 27.3 | 1.860 | 2.69 |
| 1 µM | 1.200 | 31.9 | 78.6 | 0.684 | 23.80 | 14.70 | 194.0 | 0.960 | 4.8 | 36.2 | 0.194 | 0.060 | 24.6 | 1.770 | 2.44 |
| 2.5 µM | 1.957 | 33.0 | 127.5 | 0.458 | 64.38 | 16.71 | 189.1 | 2.562 | 4.2 | 36.2 | 0.189 | 0.060 | 28.6 | 2.024 | 2.24 |
| 5 µM | 1.862 | 32.4 | 137.5 | 0.504 | 63.13 | 13.61 | 196.4 | 2.558 | 4.0 | 36.8 | 0.196 | 0.058 | 26.1 | 1.694 | 2.28 |
| 10 µM | 1.080 | 28.2 | 83.2 | 0.712 | 22.80 | 9.39 | 172.0 | 0.817 | 3.9 | 37.5 | 0.172 | 0.064 | 16.7 | 1.130 | 2.12 |
| 25 µM | 1.170 | 28.5 | 87.8 | 0.733 | 22.80 | 9.21 | 170.0 | 0.838 | 4.0 | 37.7 | 0.170 | 0.063 | 17.1 | 1.100 | 2.12 |
| 50 µM | 1.000 | 27.4 | 83.7 | 0.697 | 21.00 | 7.76 | 163.0 | 0.627 | 3.6 | 37.7 | 0.163 | 0.063 | 13.8 | 0.877 | 2.05 |
| 100 µM | 0.829 | 29.0 | 78.2 | 0.704 | 19.90 | 7.37 | 168.0 | 0.519 | 3.9 | 40.2 | 0.168 | 0.063 | 13.6 | 0.838 | 2.20 |

| **DiNP_3**  **steroid concentration** | aldosterone | androstenedione | cortisol | cortisone | corticosterone | 11-deoxycorticosterone | 11-deoxycortisol | 21-deoxycortisol | DHEA | DHEAS | DHT | estradiol | 17-OH-P | progesterone | testosterone |
| --- | --- | --- | --- | --- | --- | --- | --- | --- | --- | --- | --- | --- | --- | --- | --- |
| 1% DMSO | 0.018 | 80.0 | 9.4 | 1.520 | 0.20 | 2.51 | 182.0 | 0.105 | 9.3 | 146.0 | 0.182 | 0.253 | 67.5 | 0.811 | 5.76 |
| 1 nM | 0.018 | 77.6 | 9.5 | 1.460 | 0.21 | 2.49 | 178.0 | 0.111 | 8.5 | 139.0 | 0.178 | 0.265 | 66.3 | 0.788 | 5.46 |
| 50 nM | 0.018 | 74.0 | 9.2 | 1.470 | 0.19 | 2.49 | 176.0 | 0.095 | 8.8 | 137.0 | 0.176 | 0.263 | 63.4 | 0.788 | 5.35 |
| 100 nM | 0.018 | 77.8 | 8.8 | 1.380 | 0.19 | 2.63 | 181.0 | 0.097 | 9.8 | 138.0 | 0.181 | 0.268 | 63.7 | 0.768 | 5.40 |
| 250 nM | 0.019 | 76.5 | 9.5 | 1.300 | 0.22 | 2.67 | 174.0 | 0.120 | 9.6 | 134.0 | 0.174 | 0.267 | 63.2 | 0.715 | 5.28 |
| 500 nM | 0.020 | 83.4 | 11.5 | 1.290 | 0.36 | 3.00 | 186.0 | 0.176 | 10.8 | 143.0 | 0.186 | 0.289 | 69.6 | 0.850 | 5.69 |
| 1 µM | 0.034 | 89.9 | 18.0 | 1.270 | 0.82 | 3.87 | 209.0 | 0.432 | 11.6 | 150.0 | 0.209 | 0.303 | 84.6 | 1.080 | 6.04 |
| 2.5 µM | 0.083 | 81.7 | 24.5 | 0.967 | 1.27 | 3.70 | 203.7 | 0.807 | 9.4 | 146.0 | 0.204 | 0.287 | 79.8 | 0.958 | 5.31 |
| 5 µM | 0.082 | 75.8 | 26.9 | 0.986 | 1.37 | 2.93 | 195.0 | 0.851 | 9.1 | 137.8 | 0.195 | 0.329 | 65.2 | 0.677 | 4.78 |
| 10 µM | 0.055 | 69.8 | 27.0 | 1.280 | 1.14 | 1.88 | 179.0 | 0.455 | 8.3 | 148.0 | 0.179 | 0.349 | 35.6 | 0.326 | 4.93 |
| 25 µM | 0.051 | 64.5 | 24.3 | 1.220 | 1.01 | 1.75 | 173.0 | 0.336 | 8.0 | 136.0 | 0.173 | 0.340 | 27.7 | 0.246 | 4.59 |
| 50 µM | 0.029 | 61.9 | 21.6 | 1.220 | 0.83 | 1.51 | 177.0 | 0.209 | 7.0 | 138.0 | 0.177 | 0.366 | 18.5 | 0.150 | 4.73 |
| 100 µM | 0.024 | 57.6 | 17.9 | 1.210 | 0.56 | 1.18 | 179.0 | 0.138 | 7.0 | 149.0 | 0.179 | 0.372 | 12.9 | 0.075 | 4.96 |

| **DEHA_1**  **steroid concentration** | aldosterone | androstenedione | cortisol | cortisone | corticosterone | 11-deoxycorticosterone | 11-deoxycortisol | 21-deoxycortisol | DHEA | DHEAS | DHT | estradiol | 17-OH-P | progesterone | testosterone |
| --- | --- | --- | --- | --- | --- | --- | --- | --- | --- | --- | --- | --- | --- | --- | --- |
| 1% DMSO | 0.279 | 53.8 | 48.2 | 1.210 | 8.76 | 14.60 | 235.0 | 0.511 | 11.4 | 157.0 | 0.235 | 0.051 | 67.5 | 6.07 | 5.21 |
| 1 nM | 0.277 | 55.7 | 48.5 | 1.160 | 9.06 | 15.00 | 246.0 | 0.532 | 12.7 | 152.0 | 0.246 | 0.049 | 68.7 | 6.00 | 5.22 |
| 50 nM | 0.187 | 57.3 | 34.6 | 0.933 | 7.10 | 13.20 | 198.0 | 0.363 | 12.2 | 137.0 | 0.198 | 0.038 | 61.0 | 5.26 | 4.36 |
| 100 nM | 0.178 | 56.9 | 32.6 | 0.891 | 6.94 | 13.10 | 194.0 | 0.337 | 12.6 | 132.0 | 0.194 | 0.036 | 58.6 | 5.00 | 4.16 |
| 250 nM | 0.174 | 57.9 | 32.6 | 0.868 | 7.00 | 13.20 | 191.0 | 0.349 | 12.2 | 131.0 | 0.191 | 0.036 | 57.7 | 4.95 | 4.11 |
| 500 nM | 0.190 | 57.7 | 35.4 | 0.909 | 7.51 | 13.80 | 201.0 | 0.389 | 13.2 | 134.0 | 0.201 | 0.036 | 60.2 | 5.08 | 4.22 |
| 1 µM | 0.210 | 57.0 | 38.1 | 0.971 | 7.86 | 13.80 | 213.0 | 0.416 | 12.9 | 141.0 | 0.213 | 0.038 | 62.0 | 5.14 | 4.57 |
| 2.5 µM | 0.303 | 55.5 | 48.6 | 1.184 | 8.67 | 14.65 | 246.3 | 0.542 | 12.5 | 120.7 | 0.246 | 0.051 | 66.1 | 5.36 | 5.20 |
| 5 µM | 0.239 | 57.7 | 49.3 | 1.152 | 8.90 | 14.34 | 251.9 | 0.539 | 13.5 | 123.0 | 0.252 | 0.051 | 66.6 | 5.02 | 5.26 |
| 10 µM | 0.273 | 60.5 | 44.6 | 0.937 | 10.10 | 15.30 | 230.0 | 0.548 | 14.3 | 142.0 | 0.230 | 0.043 | 68.9 | 5.62 | 4.74 |
| 25 µM | 0.280 | 60.2 | 43.1 | 0.795 | 11.10 | 16.40 | 216.0 | 0.546 | 15.5 | 133.0 | 0.216 | 0.038 | 65.7 | 5.79 | 4.20 |
| 50 µM | 0.278 | 62.0 | 43.0 | 0.777 | 11.70 | 17.00 | 210.0 | 0.562 | 15.2 | 134.0 | 0.210 | 0.036 | 64.8 | 5.94 | 4.04 |
| 100 µM | 0.324 | 64.3 | 49.8 | 0.852 | 13.10 | 17.60 | 224.0 | 0.618 | 16.6 | 144.0 | 0.224 | 0.041 | 65.9 | 5.98 | 4.18 |

| **DEHA_2**  **steroid concentration** | aldosterone | androstenedione | cortisol | cortisone | corticosterone | 11-deoxycorticosterone | 11-deoxycortisol | 21-deoxycortisol | DHEA | DHEAS | DHT | estradiol | 17-OH-P | progesterone | testosterone |
| --- | --- | --- | --- | --- | --- | --- | --- | --- | --- | --- | --- | --- | --- | --- | --- |
| 1% DMSO | 0.200 | 52.4 | 37.7 | 1.120 | 7.20 | 13.90 | 213.0 | 0.353 | 10.3 | 131.0 | 0.213 | 0.035 | 62.2 | 6.06 | 4.79 |
| 1 nM | 0.178 | 56.3 | 34.0 | 0.967 | 6.97 | 13.70 | 206.0 | 0.334 | 12.0 | 126.0 | 0.206 | 0.033 | 61.6 | 5.49 | 4.66 |
| 50 nM | 0.178 | 56.0 | 33.4 | 0.937 | 6.94 | 13.40 | 201.0 | 0.332 | 11.7 | 125.0 | 0.201 | 0.033 | 61.7 | 5.48 | 4.48 |
| 100 nM | 0.190 | 55.4 | 34.1 | 0.937 | 7.07 | 13.60 | 202.0 | 0.345 | 12.2 | 124.0 | 0.202 | 0.033 | 60.9 | 5.43 | 4.53 |
| 250 nM | 0.167 | 58.1 | 32.9 | 0.945 | 7.01 | 13.60 | 205.0 | 0.336 | 12.9 | 127.0 | 0.205 | 0.033 | 61.6 | 5.25 | 4.51 |
| 500 nM | 0.218 | 58.4 | 39.0 | 1.020 | 8.00 | 14.90 | 222.0 | 0.415 | 12.9 | 134.0 | 0.222 | 0.036 | 65.8 | 5.68 | 4.98 |
| 1 µM | 0.231 | 56.3 | 40.9 | 1.050 | 8.52 | 15.00 | 227.0 | 0.458 | 13.2 | 132.0 | 0.227 | 0.039 | 65.4 | 5.61 | 5.04 |
| 2.5 µM | 0.162 | 57.6 | 36.8 | 1.025 | 7.00 | 13.86 | 220.9 | 0.371 | 11.9 | 140.3 | 0.221 | 0.036 | 61.6 | 5.31 | 4.94 |
| 5 µM | 0.153 | 58.3 | 32.1 | 0.865 | 6.66 | 13.32 | 206.0 | 0.317 | 13.0 | 124.9 | 0.206 | 0.030 | 57.5 | 4.74 | 4.37 |
| 10 µM | 0.229 | 61.9 | 39.9 | 0.906 | 9.47 | 15.90 | 224.0 | 0.450 | 14.2 | 127.0 | 0.224 | 0.035 | 69.6 | 5.87 | 4.86 |
| 25 µM | 0.291 | 61.6 | 46.0 | 0.886 | 11.90 | 17.40 | 237.0 | 0.578 | 14.1 | 130.0 | 0.237 | 0.037 | 70.9 | 6.31 | 4.84 |
| 50 µM | 0.385 | 65.5 | 54.3 | 0.892 | 14.30 | 19.50 | 255.0 | 0.693 | 15.3 | 138.0 | 0.255 | 0.042 | 74.5 | 6.98 | 5.16 |
| 100 µM | 0.425 | 67.2 | 59.5 | 0.926 | 15.70 | 20.00 | 264.0 | 0.760 | 16.9 | 139.0 | 0.264 | 0.044 | 74.3 | 6.90 | 5.14 |

| **DEHA_3**  **steroid concentration** | aldosterone | androstenedione | cortisol | cortisone | corticosterone | 11-deoxycorticosterone | 11-deoxycortisol | 21-deoxycortisol | DHEA | DHEAS | DHT | estradiol | 17-OH-P | progesterone | testosterone |
| --- | --- | --- | --- | --- | --- | --- | --- | --- | --- | --- | --- | --- | --- | --- | --- |
| 1% DMSO | 0.221 | 53.2 | 38.7 | 1.080 | 7.91 | 14.30 | 217.0 | 0.399 | 10.2 | 138.0 | 0.217 | 0.037 | 63.4 | 6.11 | 4.72 |
| 1 nM | 0.222 | 54.7 | 36.9 | 0.971 | 7.69 | 14.20 | 210.0 | 0.399 | 11.6 | 126.0 | 0.210 | 0.038 | 60.3 | 5.64 | 4.37 |
| 50 nM | 0.232 | 58.6 | 41.2 | 1.130 | 8.13 | 14.30 | 227.0 | 0.417 | 12.3 | 144.0 | 0.227 | 0.043 | 66.0 | 5.80 | 5.27 |
| 100 nM | 0.219 | 55.3 | 38.1 | 1.060 | 7.90 | 14.30 | 220.0 | 0.390 | 11.5 | 130.0 | 0.220 | 0.038 | 63.2 | 5.88 | 4.70 |
| 250 nM | 0.268 | 53.5 | 41.7 | 1.070 | 8.92 | 15.10 | 226.0 | 0.495 | 12.0 | 125.0 | 0.226 | 0.041 | 62.5 | 5.90 | 4.66 |
| 500 nM | 0.218 | 56.9 | 37.7 | 1.020 | 8.10 | 14.70 | 218.0 | 0.395 | 12.3 | 128.0 | 0.218 | 0.037 | 62.4 | 5.76 | 4.68 |
| 1 µM | 0.211 | 56.1 | 35.6 | 0.955 | 8.09 | 14.70 | 212.0 | 0.405 | 11.7 | 122.0 | 0.212 | 0.033 | 61.5 | 5.66 | 4.46 |
| 2.5 µM | 0.167 | 54.7 | 33.1 | 0.921 | 7.23 | 14.06 | 206.7 | 0.362 | 10.6 | 132.3 | 0.207 | 0.033 | 61.9 | 5.15 | 4.44 |
| 5 µM | 0.237 | 57.0 | 33.7 | 0.842 | 8.16 | 14.68 | 205.0 | 0.390 | 12.1 | 120.8 | 0.205 | 0.036 | 60.3 | 4.98 | 4.10 |
| 10 µM | 0.225 | 58.9 | 37.6 | 0.898 | 9.10 | 15.30 | 218.0 | 0.455 | 12.6 | 122.0 | 0.218 | 0.035 | 65.3 | 5.83 | 4.48 |
| 25 µM | 0.309 | 60.7 | 47.1 | 0.911 | 12.30 | 17.70 | 237.0 | 0.626 | 13.2 | 128.0 | 0.237 | 0.040 | 69.5 | 6.66 | 4.73 |
| 50 µM | 0.339 | 62.7 | 49.2 | 0.890 | 13.90 | 18.80 | 241.0 | 0.657 | 14.9 | 131.0 | 0.241 | 0.040 | 70.2 | 6.84 | 4.61 |
| 100 µM | 0.368 | 62.7 | 51.9 | 0.837 | 14.50 | 19.00 | 236.0 | 0.626 | 15.4 | 129.0 | 0.236 | 0.039 | 66.4 | 6.74 | 4.31 |

| **DEHT_1**  **steroid concentration** | aldosterone | androstenedione | cortisol | cortisone | corticosterone | 11-deoxycorticosterone | 11-deoxycortisol | 21-deoxycortisol | DHEA | DHEAS | DHT | estradiol | 17-OH-P | progesterone | testosterone |
| --- | --- | --- | --- | --- | --- | --- | --- | --- | --- | --- | --- | --- | --- | --- | --- |
| 1% DMSO | 0.191 | 33.0 | 31.8 | 0.715 | 7.81 | 11.70 | 174.0 | 0.249 | 4.3 | 29.9 | 0.174 | 0.036 | 31.6 | 1.890 | 2.59 |
| 1 nM | 0.190 | 33.0 | 31.6 | 0.697 | 7.63 | 11.60 | 170.0 | 0.235 | 4.1 | 28.3 | 0.170 | 0.033 | 31.7 | 1.860 | 2.70 |
| 50 nM | 0.183 | 33.1 | 30.2 | 0.666 | 7.63 | 12.10 | 174.0 | 0.231 | 4.1 | 27.0 | 0.174 | 0.039 | 31.8 | 1.900 | 2.62 |
| 100 nM | 0.164 | 33.3 | 28.2 | 0.639 | 6.86 | 10.90 | 165.0 | 0.212 | 4.0 | 26.4 | 0.165 | 0.035 | 30.0 | 1.720 | 2.59 |
| 250 nM | 0.164 | 35.8 | 29.2 | 0.639 | 6.98 | 11.80 | 172.0 | 0.211 | 4.0 | 26.9 | 0.172 | 0.035 | 30.7 | 1.830 | 2.71 |
| 500 nM | 0.193 | 35.7 | 31.8 | 0.653 | 7.75 | 12.40 | 173.0 | 0.228 | 3.8 | 26.8 | 0.173 | 0.038 | 31.3 | 1.900 | 2.75 |
| 1 µM | 0.220 | 35.8 | 33.1 | 0.664 | 8.35 | 13.60 | 177.0 | 0.252 | 4.0 | 27.8 | 0.177 | 0.040 | 30.1 | 1.960 | 2.78 |
| 2.5 µM | 0.402 | 50.1 | 50.9 | 0.611 | 25.42 | 25.13 | 231.6 | 0.579 | 6.3 | 37.5 | 0.232 | 0.048 | 31.2 | 2.115 | 3.26 |
| 5 µM | 0.397 | 48.1 | 48.2 | 0.558 | 22.57 | 23.16 | 221.4 | 0.490 | 5.8 | 32.6 | 0.221 | 0.040 | 30.7 | 1.893 | 3.21 |
| 10 µM | 0.291 | 47.8 | 39.6 | 0.667 | 11.30 | 17.70 | 203.0 | 0.312 | 5.2 | 33.1 | 0.203 | 0.044 | 26.0 | 1.870 | 3.19 |
| 25 µM | 0.313 | 49.2 | 40.9 | 0.635 | 11.90 | 19.40 | 208.0 | 0.309 | 5.3 | 33.3 | 0.208 | 0.045 | 23.9 | 1.800 | 3.19 |
| 50 µM | 0.423 | 53.2 | 45.8 | 0.602 | 14.90 | 20.80 | 214.0 | 0.342 | 6.1 | 36.8 | 0.214 | 0.050 | 20.0 | 1.620 | 3.27 |
| 100 µM | 0.479 | 56.7 | 49.1 | 0.607 | 15.80 | 20.20 | 207.0 | 0.331 | 6.5 | 38.4 | 0.207 | 0.049 | 16.4 | 1.360 | 3.28 |

| **DEHT_2**  **steroid concentration** | aldosterone | androstenedione | cortisol | cortisone | corticosterone | 11-deoxycorticosterone | 11-deoxycortisol | 21-deoxycortisol | DHEA | DHEAS | DHT | estradiol | 17-OH-P | progesterone | testosterone |
| --- | --- | --- | --- | --- | --- | --- | --- | --- | --- | --- | --- | --- | --- | --- | --- |
| 1% DMSO | 0.152 | 32.3 | 27.4 | 0.703 | 6.35 | 10.80 | 166.0 | 0.197 | 3.9 | 26.7 | 0.166 | 0.036 | 30.9 | 1.89 | 2.54 |
| 1 nM | 0.160 | 33.7 | 28.5 | 0.701 | 6.36 | 10.70 | 169.0 | 0.201 | 3.9 | 26.2 | 0.169 | 0.036 | 33.0 | 1.82 | 2.69 |
| 50 nM | 0.157 | 34.6 | 28.1 | 0.736 | 6.43 | 11.40 | 178.0 | 0.205 | 3.9 | 26.2 | 0.178 | 0.039 | 33.8 | 1.86 | 2.71 |
| 100 nM | 0.141 | 33.7 | 26.4 | 0.692 | 5.98 | 10.90 | 167.0 | 0.187 | 3.7 | 24.8 | 0.167 | 0.036 | 31.2 | 1.81 | 2.61 |
| 250 nM | 0.147 | 35.1 | 25.9 | 0.661 | 6.12 | 11.50 | 170.0 | 0.176 | 3.8 | 24.1 | 0.170 | 0.040 | 30.7 | 1.78 | 2.61 |
| 500 nM | 0.158 | 36.2 | 28.1 | 0.674 | 6.56 | 11.50 | 171.0 | 0.199 | 3.8 | 27.3 | 0.171 | 0.037 | 31.6 | 1.79 | 2.78 |
| 1 µM | 0.182 | 40.8 | 32.0 | 0.712 | 7.45 | 12.80 | 183.0 | 0.234 | 4.4 | 29.6 | 0.183 | 0.042 | 31.5 | 1.83 | 3.03 |
| 2.5 µM | 0.292 | 44.2 | 42.8 | 0.567 | 22.02 | 24.75 | 207.8 | 0.450 | 5.0 | 29.9 | 0.208 | 0.046 | 29.4 | 2.67 | 2.94 |
| 5 µM | 0.256 | 45.4 | 40.5 | 0.605 | 16.44 | 20.64 | 204.3 | 0.396 | 5.2 | 29.5 | 0.204 | 0.046 | 32.3 | 2.14 | 3.11 |
| 10 µM | 0.199 | 45.2 | 32.6 | 0.707 | 8.71 | 16.40 | 192.0 | 0.243 | 5.0 | 29.7 | 0.192 | 0.042 | 28.6 | 1.93 | 2.99 |
| 25 µM | 0.205 | 46.2 | 33.3 | 0.711 | 8.57 | 15.70 | 196.0 | 0.246 | 5.0 | 30.6 | 0.196 | 0.042 | 28.3 | 1.87 | 3.09 |
| 50 µM | 0.325 | 55.8 | 40.2 | 0.618 | 12.40 | 20.40 | 221.0 | 0.308 | 6.0 | 34.3 | 0.221 | 0.046 | 22.1 | 1.66 | 3.37 |
| 100 µM | 0.273 | 52.0 | 34.8 | 0.603 | 10.40 | 18.10 | 202.0 | 0.254 | 5.6 | 32.2 | 0.202 | 0.045 | 22.2 | 1.50 | 3.16 |

| **DEHT_3**  **steroid concentration** | aldosterone | androstenedione | cortisol | cortisone | corticosterone | 11-deoxycorticosterone | 11-deoxycortisol | 21-deoxycortisol | DHEA | DHEAS | DHT | estradiol | 17-OH-P | progesterone | testosterone |
| --- | --- | --- | --- | --- | --- | --- | --- | --- | --- | --- | --- | --- | --- | --- | --- |
| 1% DMSO | 0.022 | 75.0 | 12.1 | 1.960 | 0.22 | 2.34 | 182.0 | 0.144 | 9.8 | 175.0 | 0.182 | 0.266 | 73.5 | 0.919 | 5.75 |
| 1 nM | 0.020 | 72.4 | 12.2 | 1.940 | 0.20 | 2.53 | 182.0 | 0.152 | 8.9 | 165.0 | 0.182 | 0.273 | 75.2 | 0.979 | 5.56 |
| 50 nM | 0.016 | 73.8 | 11.5 | 1.920 | 0.23 | 2.47 | 185.0 | 0.143 | 9.7 | 163.0 | 0.185 | 0.285 | 71.8 | 0.938 | 5.60 |
| 100 nM | 0.020 | 73.0 | 10.9 | 1.770 | 0.24 | 2.66 | 180.0 | 0.134 | 8.8 | 147.0 | 0.180 | 0.281 | 69.6 | 0.930 | 5.31 |
| 250 nM | 0.021 | 79.4 | 11.4 | 1.720 | 0.26 | 2.72 | 185.0 | 0.141 | 8.8 | 158.0 | 0.185 | 0.303 | 72.1 | 0.908 | 5.68 |
| 500 nM | 0.020 | 77.6 | 12.1 | 1.680 | 0.25 | 2.73 | 184.0 | 0.168 | 8.9 | 162.0 | 0.184 | 0.307 | 73.1 | 0.953 | 5.66 |
| 1 µM | 0.033 | 94.6 | 17.6 | 1.690 | 0.67 | 4.10 | 215.0 | 0.365 | 11.3 | 180.0 | 0.215 | 0.337 | 84.1 | 1.180 | 6.48 |
| 2.5 µM | 0.051 | 98.2 | 17.3 | 1.538 | 0.61 | 4.46 | 221.5 | 0.280 | 12.7 | 186.3 | 0.222 | 0.325 | 66.6 | 1.043 | 6.35 |
| 5 µM | 0.061 | 99.4 | 21.6 | 1.671 | 0.75 | 5.38 | 251.1 | 0.376 | 11.8 | 192.9 | 0.251 | 0.371 | 76.0 | 1.243 | 6.74 |
| 10 µM | 0.055 | 114.0 | 24.2 | 1.570 | 1.38 | 5.19 | 225.0 | 0.632 | 13.7 | 195.0 | 0.225 | 0.350 | 76.2 | 1.330 | 6.39 |
| 25 µM | 0.066 | 116.0 | 26.7 | 1.570 | 1.52 | 5.38 | 229.0 | 0.690 | 15.1 | 206.0 | 0.229 | 0.357 | 74.6 | 1.340 | 6.47 |
| 50 µM | 0.080 | 125.0 | 31.7 | 1.620 | 1.85 | 5.02 | 227.0 | 0.750 | 17.3 | 227.0 | 0.227 | 0.373 | 57.5 | 1.070 | 6.41 |
| 100 µM | 0.057 | 147.0 | 31.1 | 1.980 | 1.54 | 4.05 | 242.0 | 0.462 | 22.6 | 288.0 | 0.242 | 0.417 | 49.3 | 0.539 | 7.80 |

| **DINCH_1**  **steroid concentration** | aldosterone | androstenedione | cortisol | cortisone | corticosterone | 11-deoxycorticosterone | 11-deoxycortisol | 21-deoxycortisol | DHEA | DHEAS | DHT | estradiol | 17-OH-P | progesterone | testosterone |
| --- | --- | --- | --- | --- | --- | --- | --- | --- | --- | --- | --- | --- | --- | --- | --- |
| 1% DMSO | 3.680 | 39.3 | 141.0 | 1.020 | 47.10 | 39.70 | 277.0 | 2.24 | 6.7 | 1190.0 | 0.277 | 0.119 | 55.4 | 14.50 | 4.33 |
| 1 nM | 5.910 | 43.9 | 183.0 | 1.150 | 59.70 | 43.50 | 309.0 | 3.14 | 7.7 | 1460.0 | 0.309 | 0.146 | 58.3 | 14.90 | 4.86 |
| 50 nM | 7.070 | 33.4 | 196.0 | 1.210 | 61.70 | 38.20 | 280.0 | 3.14 | 6.1 | 1740.0 | 0.280 | 0.174 | 40.4 | 11.40 | 3.81 |
| 100 nM | 7.860 | 27.6 | 218.0 | 1.300 | 64.10 | 34.00 | 268.0 | 3.13 | 5.0 | 1900.0 | 0.268 | 0.190 | 28.7 | 8.16 | 3.31 |
| 250 nM | 7.850 | 26.9 | 210.0 | 1.220 | 62.90 | 33.40 | 258.0 | 3.22 | 4.7 | 1770.0 | 0.258 | 0.177 | 29.0 | 8.26 | 3.27 |
| 500 nM | 8.950 | 24.9 | 227.0 | 1.310 | 67.40 | 30.60 | 248.0 | 3.69 | 5.0 | 1870.0 | 0.248 | 0.187 | 26.1 | 7.53 | 2.96 |
| 1 µM | 8.410 | 25.8 | 210.0 | 1.190 | 64.20 | 30.20 | 238.0 | 3.71 | 5.1 | 1620.0 | 0.238 | 0.162 | 28.9 | 8.44 | 3.04 |
| 2.5 µM | 16.279 | 45.7 | 340.2 | 0.695 | 289.68 | 74.93 | 364.7 | 13.88 | 8.3 | 1198.6 | 0.365 | 0.158 | 66.6 | 17.09 | 4.64 |
| 5 µM | 23.925 | 45.7 | 495.2 | 0.746 | 349.42 | 38.05 | 344.6 | 12.97 | 9.4 | 1523.5 | 0.345 | 0.204 | 29.5 | 4.83 | 4.42 |
| 10 µM | 9.000 | 44.5 | 187.0 | 0.939 | 80.20 | 46.80 | 275.0 | 4.67 | 13.4 | 1310.0 | 0.275 | 0.131 | 59.4 | 19.40 | 3.98 |
| 25 µM | 9.190 | 51.9 | 194.0 | 0.948 | 87.00 | 49.80 | 302.0 | 3.14 | 22.2 | 1620.0 | 0.302 | 0.162 | 43.8 | 14.00 | 4.88 |
| 50 µM | 9.410 | 48.4 | 202.0 | 1.000 | 85.70 | 46.80 | 300.0 | 2.97 | 20.3 | 1670.0 | 0.300 | 0.167 | 36.2 | 11.50 | 4.69 |
| 100 µM | 9.870 | 42.3 | 202.0 | 1.000 | 83.60 | 45.60 | 288.0 | 3.51 | 16.3 | 1680.0 | 0.288 | 0.168 | 40.0 | 13.80 | 4.10 |

| **DINCH_2**  **steroid concentration** | aldosterone | androstenedione | cortisol | cortisone | corticosterone | 11-deoxycorticosterone | 11-deoxycortisol | 21-deoxycortisol | DHEA | DHEAS | DHT | estradiol | 17-OH-P | progesterone | testosterone |
| --- | --- | --- | --- | --- | --- | --- | --- | --- | --- | --- | --- | --- | --- | --- | --- |
| 1% DMSO | 1.990 | 55.1 | 97.1 | 0.855 | 34.10 | 30.20 | 267.0 | 1.270 | 9.9 | 82.0 | 0.267 | 0.069 | 63.3 | 11.00 | 4.60 |
| 1 nM | 1.890 | 71.3 | 103.0 | 0.946 | 34.20 | 29.30 | 293.0 | 1.180 | 13.3 | 98.7 | 0.293 | 0.077 | 70.1 | 10.80 | 5.85 |
| 50 nM | 2.500 | 76.3 | 123.0 | 0.961 | 40.60 | 33.50 | 319.0 | 1.750 | 17.7 | 103.0 | 0.319 | 0.082 | 72.1 | 10.20 | 5.99 |
| 100 nM | 2.480 | 67.0 | 119.0 | 0.979 | 38.90 | 31.10 | 299.0 | 1.710 | 16.4 | 99.9 | 0.299 | 0.078 | 67.9 | 10.20 | 5.49 |
| 250 nM | 2.870 | 67.9 | 128.0 | 0.975 | 44.20 | 33.70 | 311.0 | 1.990 | 16.4 | 97.9 | 0.311 | 0.081 | 71.5 | 11.50 | 5.47 |
| 500 nM | 4.550 | 59.1 | 149.0 | 0.922 | 55.30 | 34.70 | 288.0 | 3.400 | 16.8 | 89.8 | 0.288 | 0.087 | 72.3 | 13.10 | 4.73 |
| 1 µM | 4.860 | 56.2 | 154.0 | 0.963 | 56.10 | 34.60 | 282.0 | 3.420 | 15.1 | 90.8 | 0.282 | 0.089 | 69.5 | 13.20 | 4.64 |
| 2.5 µM | 6.599 | 58.3 | 211.7 | 0.607 | 177.89 | 49.16 | 311.2 | 6.762 | 10.9 | 80.9 | 0.311 | 0.098 | 70.3 | 13.02 | 4.64 |
| 5 µM | 6.895 | 68.5 | 263.1 | 0.607 | 172.37 | 22.76 | 299.3 | 4.081 | 14.0 | 101.5 | 0.299 | 0.120 | 33.3 | 3.57 | 5.23 |
| 10 µM | 6.190 | 53.4 | 144.0 | 0.796 | 69.00 | 38.10 | 255.0 | 4.290 | 17.4 | 76.6 | 0.255 | 0.086 | 78.1 | 20.80 | 3.99 |
| 25 µM | 2.600 | 66.9 | 100.0 | 0.623 | 53.70 | 41.90 | 265.0 | 0.942 | 20.9 | 93.0 | 0.265 | 0.079 | 42.0 | 12.20 | 5.20 |
| 50 µM | 4.600 | 68.7 | 134.0 | 0.758 | 71.30 | 47.70 | 289.0 | 1.960 | 26.8 | 95.6 | 0.289 | 0.094 | 62.7 | 18.40 | 5.30 |
| 100 µM | 5.430 | 61.8 | 142.0 | 0.788 | 71.70 | 44.10 | 275.0 | 2.830 | 22.4 | 87.5 | 0.275 | 0.091 | 71.5 | 21.00 | 4.69 |

| **DINCH_3**  **steroid concentration** | aldosterone | androstenedione | cortisol | cortisone | corticosterone | 11-deoxycorticosterone | 11-deoxycortisol | 21-deoxycortisol | DHEA | DHEAS | DHT | estradiol | 17-OH-P | progesterone | testosterone |
| --- | --- | --- | --- | --- | --- | --- | --- | --- | --- | --- | --- | --- | --- | --- | --- |
| 1% DMSO | 6.430 | 40.0 | 206.0 | 1.520 | 54.20 | 30.00 | 234.0 | 2.40 | 6.1 | 94.6 | 0.234 | 0.172 | 50.0 | 10.00 | 3.00 |
| 1 nM | 2.320 | 49.6 | 106.0 | 0.882 | 36.80 | 35.00 | 276.0 | 1.68 | 8.7 | 83.8 | 0.276 | 0.086 | 63.7 | 13.00 | 4.70 |
| 50 nM | 4.140 | 38.9 | 143.0 | 0.963 | 47.50 | 34.80 | 263.0 | 2.25 | 7.2 | 83.4 | 0.263 | 0.118 | 49.0 | 10.90 | 3.80 |
| 100 nM | 5.440 | 28.2 | 169.0 | 1.050 | 56.30 | 34.80 | 267.0 | 3.03 | 5.1 | 88.0 | 0.267 | 0.150 | 36.8 | 10.30 | 3.64 |
| 250 nM | 6.030 | 23.2 | 184.0 | 1.140 | 56.90 | 28.70 | 245.0 | 2.94 | 4.3 | 90.3 | 0.245 | 0.162 | 25.8 | 7.58 | 3.07 |
| 500 nM | 8.500 | 19.8 | 217.0 | 1.190 | 63.30 | 24.80 | 223.0 | 3.40 | 5.2 | 92.4 | 0.223 | 0.180 | 19.1 | 5.27 | 2.52 |
| 1 µM | 8.720 | 15.5 | 225.0 | 1.550 | 58.80 | 19.40 | 180.0 | 3.05 | 7.0 | 103.0 | 0.180 | 0.182 | 14.5 | 3.78 | 1.81 |
| 2.5 µM | 34.993 | 44.4 | 489.8 | 1.129 | 299.17 | 53.21 | 315.6 | 14.57 | 6.0 | 98.4 | 0.316 | 0.229 | 61.6 | 12.98 | 3.35 |
| 5 µM | 9.075 | 48.0 | 278.0 | 0.592 | 180.38 | 28.36 | 291.0 | 6.01 | 9.2 | 89.2 | 0.291 | 0.127 | 30.9 | 4.10 | 4.30 |
| 10 µM | 9.520 | 29.1 | 216.0 | 1.070 | 74.40 | 34.80 | 258.0 | 3.84 | 8.9 | 97.9 | 0.258 | 0.171 | 27.3 | 8.84 | 3.13 |
| 25 µM | 11.800 | 36.6 | 256.0 | 1.210 | 84.00 | 32.60 | 284.0 | 2.93 | 20.6 | 127.0 | 0.284 | 0.214 | 16.7 | 4.52 | 3.53 |
| 50 µM | 11.500 | 38.2 | 261.0 | 1.260 | 81.30 | 28.30 | 273.0 | 2.46 | 21.9 | 142.0 | 0.273 | 0.216 | 13.8 | 3.52 | 3.42 |
| 100 µM | 11.300 | 25.4 | 260.0 | 1.280 | 76.80 | 25.00 | 235.0 | 3.42 | 11.7 | 113.0 | 0.235 | 0.200 | 15.0 | 3.82 | 2.39 |

| **mixture_1**  **steroid concentration** | aldosterone | androstenedione | cortisol | cortisone | corticosterone | 11-deoxycorticosterone | 11-deoxycortisol | 21-deoxycortisol | DHEA | DHEAS | DHT | estradiol | 17-OH-P | progesterone | testosterone |
| --- | --- | --- | --- | --- | --- | --- | --- | --- | --- | --- | --- | --- | --- | --- | --- |
| 1% DMSO | 0.076 | 32.0 | 13.1 | 0.284 | 3.91 | 5.64 | 79.9 | 0.168 | 11.7 | 22.8 | 0.080 | 0.040 | 15.3 | 0.729 | 1.22 |
| 1 nM | 0.073 | 32.4 | 12.6 | 0.263 | 3.85 | 5.67 | 74.0 | 0.168 | 12.6 | 19.8 | 0.074 | 0.035 | 15.1 | 0.722 | 1.21 |
| 50 nM | 0.072 | 33.1 | 12.3 | 0.265 | 3.71 | 5.68 | 75.5 | 0.160 | 12.2 | 19.4 | 0.076 | 0.036 | 14.7 | 0.678 | 1.23 |
| 100 nM | 0.075 | 34.0 | 13.1 | 0.278 | 3.99 | 6.01 | 80.0 | 0.177 | 13.1 | 20.7 | 0.080 | 0.037 | 15.6 | 0.722 | 1.33 |
| 250 nM | 0.098 | 32.0 | 17.7 | 0.237 | 5.42 | 4.86 | 86.3 | 0.250 | 11.2 | 20.6 | 0.086 | 0.045 | 13.8 | 0.606 | 1.18 |
| 500 nM | 0.123 | 31.9 | 21.0 | 0.247 | 6.45 | 4.98 | 86.4 | 0.315 | 11.3 | 22.9 | 0.086 | 0.046 | 14.2 | 0.696 | 1.19 |
| 1 µM | 0.076 | 30.9 | 13.8 | 0.186 | 4.88 | 5.45 | 83.0 | 0.195 | 9.1 | 15.2 | 0.083 | 0.045 | 11.8 | 0.451 | 1.07 |
| 2.5 µM | 0.272 | 39.0 | 28.0 | 0.186 | 20.80 | 10.59 | 97.1 | 0.783 | 14.0 | 24.3 | 0.097 | 0.052 | 17.1 | 0.764 | 1.32 |
| 5 µM | 0.330 | 37.4 | 30.5 | 0.146 | 25.55 | 9.75 | 82.0 | 0.986 | 15.6 | 19.7 | 0.082 | 0.047 | 15.5 | 0.769 | 1.16 |
| 10 µM | 0.066 | 34.7 | 12.7 | 0.270 | 3.74 | 6.11 | 81.7 | 0.164 | 13.4 | 20.3 | 0.082 | 0.039 | 15.5 | 0.699 | 1.36 |
| 25 µM | 0.077 | 35.6 | 13.8 | 0.255 | 4.30 | 6.59 | 84.7 | 0.178 | 13.0 | 20.7 | 0.085 | 0.040 | 15.0 | 0.721 | 1.37 |
| 50 µM | 0.100 | 35.4 | 16.8 | 0.259 | 5.35 | 6.90 | 90.3 | 0.237 | 12.6 | 22.0 | 0.090 | 0.038 | 15.0 | 0.760 | 1.43 |
| 100 µM | 0.123 | 31.5 | 20.6 | 0.252 | 6.39 | 5.23 | 86.4 | 0.335 | 12.0 | 20.1 | 0.086 | 0.043 | 16.3 | 0.854 | 1.19 |

| **mixture_2**  **steroid concentration** | aldosterone | androstenedione | cortisol | cortison | corticosterone | 11-deoxycorticosterone | 11-deoxycortisol | 21-deoxycortisol | DHEA | DHEAS | DHT | estradiol | 17-OH-P | progesterone | testosterone |
| --- | --- | --- | --- | --- | --- | --- | --- | --- | --- | --- | --- | --- | --- | --- | --- |
| 1% DMSO | 0.0323 | 80.1 | 11.9 | 2.07 | 0.203 | 2.21 | 195 | 0.141 | 10.5 | 167 | 0.195 | 0.234 | 74.9 | 0.872 | 6.4 |
| 1 nM | 0.0304 | 68.7 | 10.6 | 1.98 | 0.207 | 1.89 | 170 | 0.110 | 11.2 | 155 | 0.170 | 0.231 | 60.1 | 0.722 | 5.43 |
| 50 nM | 0.0313 | 64.8 | 9.6 | 1.86 | 0.184 | 1.79 | 154 | 0.104 | 10.5 | 154 | 0.154 | 0.249 | 55.6 | 0.721 | 4.97 |
| 100 nM | 0.0249 | 67.9 | 9.3 | 1.67 | 0.187 | 1.88 | 164 | 0.094 | 9.99 | 142 | 0.164 | 0.245 | 54.6 | 0.689 | 5.03 |
| 250 nM | 0.0247 | 80.1 | 12.0 | 1.45 | 0.389 | 2.63 | 191 | 0.180 | 10.7 | 140 | 0.191 | 0.245 | 69.4 | 0.878 | 5.35 |
| 500 nM | 0.0350 | 84.3 | 14.5 | 1.28 | 0.571 | 2.8 | 194 | 0.254 | 12.8 | 149 | 0.194 | 0.242 | 60.2 | 0.728 | 5.34 |
| 1 µM | 0.0400 | 97.5 | 19.3 | 1.44 | 0.895 | 2.73 | 243 | 0.341 | 14.7 | 189 | 0.243 | 0.305 | 63.8 | 0.504 | 6.66 |
| 2.5 µM | 0.0832 | 88.1 | 22.3 | 1.31 | 0.925 | 3.87 | 229 | 0.573 | 11.6 | 158 | 0.229 | 0.314 | 79.9 | 1.007 | 6.48 |
| 5 µM | 0.1465 | 72.3 | 27.4 | 1.28 | 1.382 | 3.11 | 196 | 0.768 | 12.5 | 155 | 0.196 | 0.348 | 68.0 | 0.950 | 5.30 |
| 10 µM | 0.0707 | 74.4 | 29.3 | 1.43 | 1.130 | 2.30 | 209 | 0.706 | 9.96 | 154 | 0.209 | 0.322 | 47.3 | 0.669 | 4.87 |
| 25 µM | 0.0565 | 70.4 | 23.4 | 1.15 | 1.030 | 2.28 | 193 | 0.385 | 10.6 | 133 | 0.193 | 0.317 | 32.6 | 0.467 | 4.42 |
| 50 µM | 0.0426 | 72.1 | 21.2 | 1.11 | 0.842 | 2.26 | 193 | 0.273 | 8.97 | 131 | 0.193 | 0.336 | 25.1 | 0.391 | 4.51 |
| 100 µM | 0.0808 | 75.4 | 28.4 | 1.29 | 1.360 | 2.67 | 200 | 0.776 | 12.5 | 138 | 0.200 | 0.279 | 54.8 | 0.903 | 4.65 |

| **mixture_3**  **steroid concentration** | aldosterone | androstenedione | cortisol | cortison | corticosterone | 11-deoxycorticosterone | 11-deoxycortisol | 21-deoxycortisol | DHEA | DHEAS | DHT | estradiol | 17-OH-P | progesterone | testosterone |
| --- | --- | --- | --- | --- | --- | --- | --- | --- | --- | --- | --- | --- | --- | --- | --- |
| 1% DMSO | 0.0164 | 78.9 | 10.6 | 2.04 | 0.199 | 2.27 | 203 | 0.098 | 9.25 | 165 | 0.203 | 0.227 | 76.7 | 0.926 | 6.74 |
| 1 nM | 0.0168 | 77.9 | 9.77 | 1.87 | 0.191 | 2.18 | 195 | 0.091 | 9.54 | 155 | 0.195 | 0.23 | 70.5 | 0.806 | 6.37 |
| 50 nM | 0.0187 | 75.1 | 9.73 | 1.76 | 0.198 | 2.25 | 193 | 0.088 | 9.02 | 148 | 0.193 | 0.227 | 70.9 | 0.893 | 6.07 |
| 100 nM | 0.0154 | 77,0 | 9.26 | 1.68 | 0.183 | 2.29 | 198 | 0.082 | 9.14 | 149 | 0.198 | 0.231 | 67.9 | 0.81 | 6.04 |
| 250 nM | 0.0115 | 83.7 | 9.45 | 1.57 | 0.241 | 2.58 | 206 | 0.094 | 11.1 | 143 | 0.206 | 0.233 | 68.5 | 0.854 | 6.06 |
| 500 nM | 0.0125 | 82.7 | 9.67 | 1.38 | 0.261 | 2.45 | 198 | 0.097 | 9.75 | 132 | 0.198 | 0.235 | 62.9 | 0.728 | 5.69 |
| 1 µM | 0.0192 | 95.5 | 13.8 | 1.54 | 0.477 | 2.25 | 235 | 0.157 | 11.4 | 172 | 0.235 | 0.289 | 61.7 | 0.385 | 6.95 |
| 2.5 µM | 0.0569 | 90.7 | 20.4 | 1.38 | 0.841 | 3.89 | 253 | 0.372 | 11.0 | 166 | 0.253 | 0.243 | 85.6 | 1.011 | 7.26 |
| 5 µM | 0.0966 | 83.9 | 24.4 | 1.16 | 1.156 | 3.52 | 221 | 0.544 | 10.3 | 148 | 0.221 | 0.290 | 76.3 | 0.989 | 6.15 |
| 10 µM | 0.0735 | 79.9 | 32,0 | 1.41 | 1.380 | 2.48 | 229 | 0.750 | 11.1 | 152 | 0.229 | 0.316 | 52.7 | 0.693 | 5.37 |
| 25 µM | 0.0634 | 79.6 | 29.7 | 1.34 | 1.250 | 2.34 | 231 | 0.511 | 11.3 | 150 | 0.231 | 0.348 | 38.9 | 0.503 | 5.26 |
| 50 µM | 0.0398 | 69.3 | 22.6 | 1.14 | 0.820 | 1.95 | 198 | 0.290 | 8.54 | 135 | 0.198 | 0.301 | 26.4 | 0.361 | 4.40 |
| 100 µM | 0.1060 | 86.4 | 36.0 | 1.41 | 1.800 | 3.35 | 252 | 1.140 | 12.7 | 151 | 0.252 | 0.332 | 73.2 | 1.130 | 5.70 |
