# Supplementary material for: Disruptive effects of phthalates and their substitutes on adrenal steroidogenesis
Source: Front Endocrinol (Lausanne). 2026 Jan 14;16:1734184. doi: 10.3389/fendo.2025.1734184 (PMC12848149; doi:10.3389/fendo.2025.1734184)
Supplement: Supplementary file 7 [file DataSheet7.docx]

**Supplementary Material S7.** The table below summarizes the limits of detection (LOD) determined for all quantified steroids using our validated LC-MS/MS method..

| **Limit of detection (LOD)** | **[µg/L]** |
| --- | --- |
| aldosterone | 0.010 |
| androstenedione | 0.022 |
| corticosterone | 0.175 |
| cortisol | 1.520 |
| cortisone | 0.148 |
| 21-deoxycorticosterone | 0.023 |
| 11-deoxycortisol | 0.030 |
| 21-deoxycortisol | 0.027 |
| DHEA | 0.229 |
| DHEAS | 14.400 |
| DHT | 0.042 |
| estradiol | 0.030 |
| 17-OHP | 0.040 |
| progesterone | 0.030 |
| testosterone | 0.005 |
